# Membrane directed expression in *Escherichia coli* of BBA57 and other virulence factors from the Lyme disease agent *Borrelia burgdorferi*

**DOI:** 10.1038/s41598-019-53830-x

**Published:** 2019-11-26

**Authors:** Karie E. Robertson, Chloe D. Truong, Felicia M. Craciunescu, Po-Lin Chiu, Petra Fromme, Debra T. Hansen

**Affiliations:** 1 Biodesign Center for Applied Structural Discovery, Arizona State University, 1001 S. McAllister Avenue, Tempe, AZ 85287 USA; 2 School of Molecular Sciences, Arizona State University, 551 East University Drive, Tempe, AZ 85287 USA; 3 Biodesign Center for Innovations in Medicine, Arizona State University, 1001 S. McAllister Avenue, Tempe, AZ 85287 USA

**Keywords:** Membrane proteins, Protein purification, Bacterial pathogenesis

## Abstract

Membrane-embedded proteins are critical to the establishment, survival and persistence in the host of the Lyme disease bacterium *Borrelia burgdorferi* (*Bb*), but to date, there are no solved structures of transmembrane proteins representing these attractive therapeutic targets. All available structures from the genus *Borrelia* represent proteins expressed without a membrane-targeting signal peptide, thus avoiding conserved pathways that modify, fold and assemble membrane protein complexes. Towards elucidating structure and function of these critical proteins, we directed translocation of eleven expression-optimized *Bb* virulence factors, including the signal sequence, to the *Escherichia coli* membrane, of which five, BBA57, HtrA, BB0238, BB0323, and DipA, were expressed with C-terminal His-tags. P66 was also expressed using the PelB signal sequence fused to maltose binding protein. Membrane-associated BBA57 lipoprotein was solubilized by non-ionic and zwitterionic detergents. We show BBA57 translocation to the outer membrane, purification at a level sufficient for structural studies, and evidence for an α-helical multimer. Previous studies showed multiple critical roles of BBA57 in transmission, joint arthritis, carditis, weakening immune responses, and regulating other *Bb* outer surface proteins. In describing the first purification of membrane-translocated BBA57, this work will support subsequent studies that reveal the precise mechanisms of this important Lyme disease virulence factor.

## Introduction

Lyme borreliosis is the most prevalent tick-borne disease in the Northern Hemisphere^1^. The disease is caused by certain spirochetal species within *Borrelia burgdorferi* sensu lato. In the U.S., Lyme borreliosis is diagnosed in over 300,000 people annually^2^, and the primary cause is the species *Borrelia burgdorferi* sensu stricto (*Bb*)^3^. In Europe, the major agents include *Borrelia afzelii* and *Borrelia garinii*^3^. Transmission of the spirochete to mammals, including humans, occurs from ticks in the genus *Ixodes*. Infection at the skin often leads to erythema migrans. Dissemination of the spirochete in mammalian hosts leads to infection of the heart, joints, brain, and nervous system. Critical to resolving the disease is proper diagnosis, which includes clinical manifestations and may include laboratory tests, followed by treatment with the appropriate antibiotic(s)^4^.

*Bb* is a spirochete that possesses a double-membrane^5^ and proliferates extracellularly in its hosts by modulating its outer surface proteins. *Bb* is rich in outer surface lipoproteins that change in response to environmental triggers (pH; temperature; nutrient density) and that allow evasion of host immune responses via active immune suppression, antigenic variation, and physical seclusion^6,7^. Because *Bb’s* reduced genome lacks the ability to synthesize amino acids, lipids, nucleotides, and cofactors^8^, *Bb*’s survival is completely dependent on membrane-integral proteins to transport nutrients across its inner and outer membranes. Passage of ions, nutrients, as well as many drugs across biological membranes requires recognition and transport by membrane-spanning proteins.

Several membrane-translocated proteins are important for *Bb* transmission and persistence. In ticks, OspA is essential for colonization and survival^9^ and Lmp1 facilitates dissemination^10^. BB0405 is necessary for transmission from tick to mouse and for establishing infection in mice^11^. In mammals, infection requires OspC^12^, P66^13^ and BesC^14^. P66 also plays a role in dissemination^15,16^. DbpA and DbpB are critical for colonizing all tissues^17^. Lmp1 is required for *Bb* persistence^18^ and contributes to complement-independent serum resistance^18^. Some of these proteins also function as adhesins. In ticks, OspA binds the TROSPA receptor^19^, and OspC binds the saliva protein Salp15^20^. In mammals, OspC also binds plasminogen^21^, Lmp1 binds mammalian glycosaminoglycan^22^, and P66 binds β_3_-chain integrins^23,24^. DbpA and DbpB bind decorin and glycosaminoglycans that are important in the host extracellular matrix and connective tissues^25^. Some of these proteins also have demonstrated porin activity, including BB0405^26^, P66^27^ and BesC^14^, although it is unknown what are the *in vivo* roles of the porin activity^15,28^ and what are the pore substrate(s)^29^. BesC has been modeled as a trimeric β-barrel and is a homolog of the *Escherichia coli* TolC multidrug efflux pump^14^. Thus, membrane-translocated proteins are critical to the establishment, survival and persistence of *Bb* in the host.

The *Bb* genome encodes ≥20% proteins that are targeted to the membrane by N-terminal signal peptide sequences^8^. These signal peptide containing proteins are directed from the translating ribosome in the cytoplasm to the inner and outer membranes (OM). About 14% of *Bb* genes are lipoproteins^30^. These lipoproteins are also directed to the inner membrane and, after signal peptide removal, are covalently lipidated, which anchors the protein to the membrane. Some translocated (lipo)proteins are further directed to the OM, where folding and assembly may be aided by periplasmic chaperones and the spirochetal β-barrel assembly machine (BAM) complex^31,32^. In this paper, we used the term membrane-translocated protein to refer to proteins that are directed from the cytoplasm to the inner membrane by a signal peptide sequence. This term does not specify whether the protein stays in the inner membrane, is further directed to the periplasm or to the outer membrane, or is secreted.

While membrane-translocated proteins are very important for the pathogenesis of *Bb*, information on their structures is lacking, which represents a critical barrier in understanding disease pathogenesis. None of the genus *Borrelia* protein structures available in the Protein Data Bank^33^ include transmembrane domains^34^, even though transmembrane activities have been shown for P66, P13, BesC, BamA, BB0405, BB0406, DipA and Oms28^6,26,32,35,36^. The lack of such structures mainly reflects technical challenges. Furthermore, it has been suggested that transmembrane domains exist in BB0017, BB0164, BB0202, BB0412, BB0473, BB0631, and ChbB^37^. Many of the important outer surface virulence proteins in *Bb*, including OspA, OspB, OspC, OspD, P13, P66 and Lp6.6, are common components of OM multiprotein complexes^38^ that function in pathogenesis, but the structural arrangement of these complexes is unknown. There exist many structures (Supplementary Table S1) for the soluble domains of outer surface proteins (Osps), primarily lipoproteins, which are from the Lyme disease agents *B. burgdorferi* sensu stricto, *B. garinii*, *Borrelia bavariensis* and *Borrelia spielmanii*^39^ and the tick-borne relapsing fever agent *Borrelia turicatae*^40^ (Supplementary Table S1, column 3). However, each of these proteins was expressed in the cytoplasm of *E. coli* (Supplementary Table S1, column 5).

In other bacteria, several exciting structures demonstrate the necessity of membrane translocation for determining the pathogenic function of an OM protein. In these structures, small lipoproteins assemble to form large homo-multimeric channels, but only when translocated to the membrane. In the enterobacteria, these lipoproteins include the 42 kDa Wza, which assembles into a 340 kDa octamer that secretes the polysaccharides that form an immune-evading bacterial capsule^41^, and the 31 kDa CsgG, which assembles into a 250 kDa nonameric amyloid secretion channel that causes biofilm formation in the host^42^. For both Wza and CsgG, multimerization requires the lipid modification^42,43^. Additionally, the 28 kDa MlaA lipoprotein from *Klebsiella pneumoniae* forms multiprotein complexes with OmpF and OmpC/K and functions as a phospholipid translocation channel, but only when MlaA is expressed with its signal peptide^44^. Other proteins that form homo-multimeric OM channels only upon membrane translocation include the *Haemophilus influenzae* Hia adhesin^45^ and the *E. coli* TolC multidrug efflux pump^46^. Despite these important examples, to date, none of the available structures of *Bb* proteins that contain signal peptides was derived from protein that was directed to the cell membrane (Supplementary Table S1).

The aim of this study is to identify membrane-translocation upon heterologous expression in *E. coli* within eleven OM target proteins from *Bb*: BBA57, RevA, P13, HtrA, Lmp1, BB0238, BB0323, BB0405, BB0406, DipA and P66, which have roles in pathogenesis (Supplementary Table S2, shaded column), in order to support future structural studies of their membrane directed forms. Here we show, for the first time, translocation of the BBA57 lipoprotein to the *E. coli* OM, purification of this form, and evidence for its multimerization in a primarily α-helical form. BBA57 is a surface exposed^47^, immunogenic^48,49^ virulence determinant and an inflammatory agent in Lyme arthritis and carditis^47,50^. BBA57 is necessary for *Bb* transmission from tick to humans^50^ and to mice^47^ and is upregulated by temperature and by cultivation in mice^51,52^. BBA57 is encoded by the linear plasmid lp54 and is not a highly abundant protein^53^. BBA57 has homologues (>61% identity across 99% of the protein sequence) in the other Lyme disease pathogens *B. afzelii*, *Borrelia mayonii, B. garinii*, *B. bavariensis* and *B. spielmanii*. In potential pathogens, the presence of BBA57 is mixed, with one homolog in each *Borrelia valaisiana* (67% identity) and *Borrelia bissettii* (73% identity), but none in *Borrelia lusitaniae*. BBA57 homologs are absent in the tick-borne relapsing fever bacteria. Mutational analyses showed that BBA57 is upregulated 4- to 11-fold by the components of the Rrp2/RpoN/RpoS infection regulatory cascade, a major transcriptional response to the mammalian host environment and one that controls OM proteins, transporters and other proteins important for infection, colonization and transmission^52^. The Pal group recently demonstrated multiple critical roles for BBA57 in weakening host complement, neutrophil and interferon responses; in decreasing transcription of antimicrobial peptides and interferon genes; and in regulating expression of the *Bb* outer surface lipoproteins OspC, ErpP and ErpB^50^. Despite these multiple important roles, no structure has been determined for BBA57. These diverse and complex roles do not point to an obvious, discrete function for BBA57 that can be easily assayed. We expect that structures will reveal its mechanisms in pathogenesis.

## Results

### Positive expression in *E. coli* of *B. burgdorferi* BBA57, HtrA, BB0238, BB0323, and DipA proteins with C-terminal His_12_-tags

**Figure 1:**
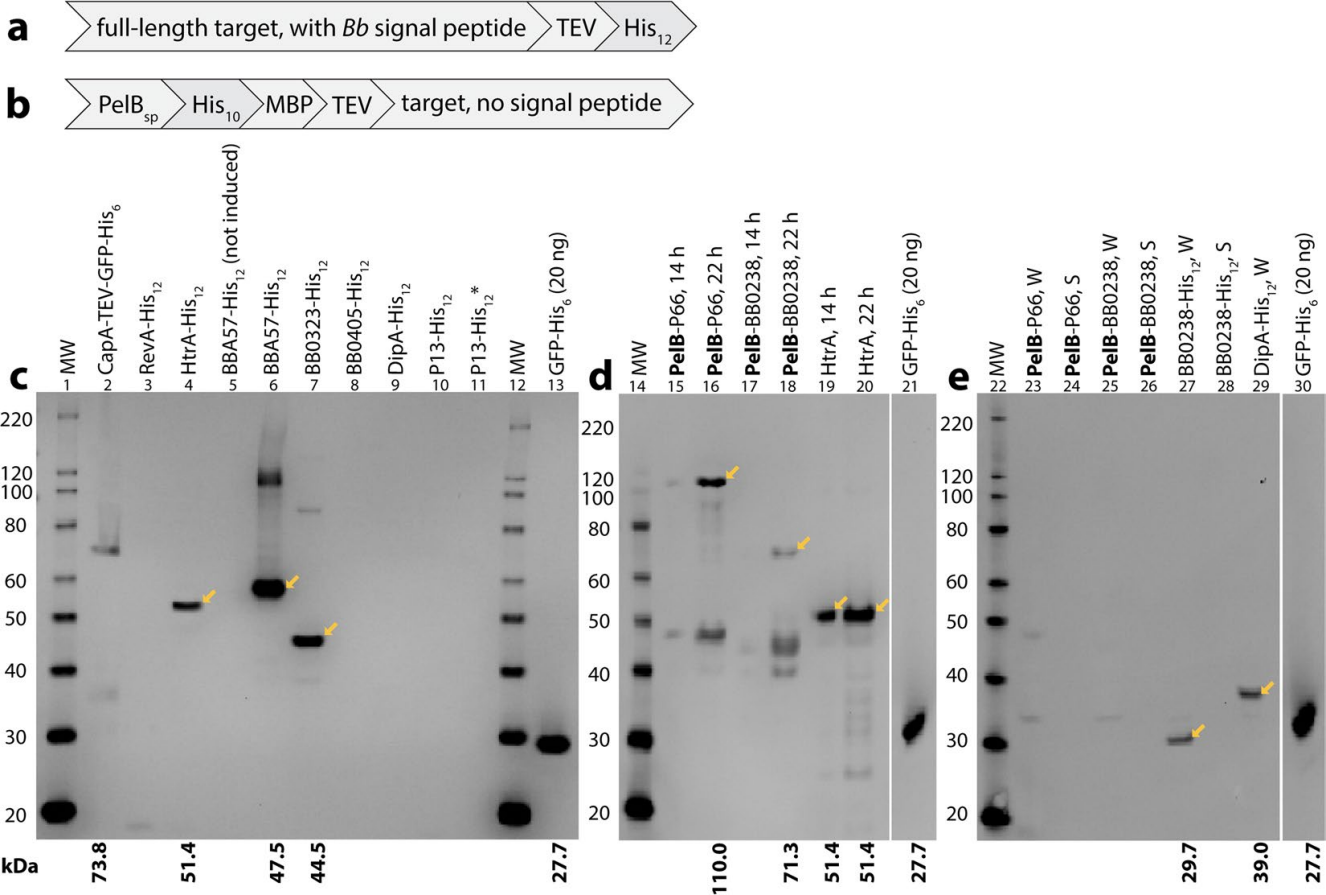
Expression of *Bb* BBA57, HtrA, BB0238, BB0323, DipA and P66 membrane proteins in *E. coli*. (**a,b**) Schematic of the expression constructs. (**a**) The full-length target (pre-protein) with a TEV protease cleavable C-terminal His_12_-tag. (**b**) The mature target protein with its predicted *Bb* signal peptide substituted with the PelB signal peptide (PelB_sp_), a His_10_ tag, maltose binding protein (MBP), and TEV protease cleavage site. (**c**) Expression in BL21-AI cultures grown at 37 °C to OD_600_ 0.5, induced with 0.2% arabinose, and harvested after 3 hr. Shown are Western blots (anti-His) of total cellular protein. Arrows indicate the possible target protein based on the expected MW, listed below each blot and lacking the cleaved signal peptide. The asterisk (*) indicates KTD101(DE3); all other cultures are BL21-AI. Per lane are cells from 10 µL of culture. Lane 2 contains a positive control for expression and purification, CapA from *Francisella tularensis*^107^, and lane 14 contains purified GFP-His_6_^107^. (**d**) Positive expression of PelB-P66, PelB-BB0238, and HtrA-His_12_ in *E. coli* BL21-AI grown at 37 °C in M9 minimal media to OD_600_ 0.8–0.9, induced with 0.1% arabinose and decreased temperature to 25 °C, and harvested after 14 h or 22 h. Per lane is 6.25 µL of culture. (**e**) Positive expression of BB0238-His_12_ and DipA-His_12_ in *E. coli* BL21-AI grown at 37 °C to OD_600_ 0.5, induced with 0.2% arabinose with a temperature change to 18 °C, and harvested after 18–20 h. Per lane is 7.5 µL of culture either before harvesting the cells by centrifugation (whole culture, W) or after centrifugation and removal of the cells (culture supernatant, S). Full gel images for (**d,e**) are in Supplementary Fig. S6. Abbreviations: MW, molecular weight.

In contrast to the existing available structures for soluble domains of membrane-anchored proteins (Supplementary Table S1), the expression clones in this paper retained an N-terminal membrane localization sequence, so that the full-length targets could be translocated to the membrane for proper folding. Each of the 11 targets contained the C-terminal sequence ENLYFQGHHHHHHHHHHHH (Fig. 1a). In this paper, these proteins are referred to as “Target-His_12_”. Expression results in this paper are summarized in Table 1. The *Bb* genes were also optimized for expression in *E. coli*. Schematics and DNA sequences are in Supplementary Figs. S1a,b and S2a,b, respectively. Sequences for the His_12_ and PelB constructs are in Supplementary Figs. S3a,b and S4a,b, respectively. The His_12_ constructs were transformed into the *E. coli* expression strain BL21(DE3), as well as into four strains (Table 2) that are typically used to overcome problems of membrane protein unfolding and toxicity by modulating expression levels through various mechanisms. For P66-His_12_, no colonies could be obtained upon transformation into any of the five strains (Table 1), even upon increasing the amount of plasmid DNA transformed from 20 to 400 ng. BB0323 colonies were obtained in C43(DE3) only upon increasing the DNA added to competent cells from 70 to 800 ng. For the other targets that failed to yield colonies in some of the strains, such as RevA, HtrA, BB0238, and DipA (Table 1), we did not systematically pursue transforming with higher amounts of DNA.

**Table 1:** Summary of expression of targets in total protein of *E. coli* as determined by anti-His Western. Abbreviations: −, not expressed; +, expressed; NoC, no colonies obtained upon transforming the strain; nd, not done.

Protein	Strain				
	C43 (DE3)	BL21 (DE3)	KTD101 (DE3)	BL21-AI	Lemo21 (DE3)
BBA57-His_12_	−	+	+	+	−
RevA-His_12_	−	NoC	NoC	−	nd
P13-His_12_	−	−	−	−	−
BbHtrA-His_12_	−	NoC	−	+	nd
Lmp1-His_12_	Could not make this clone				
BB0238-His_12_	−	NoC	NoC	+	nd
BB0323-His_12_	−	NoC	NoC	+	NoC
BB0405-His_12_	−	−	−	−	−
BB0406-His_12_	Could not make this clone				
DipA-His_12_	−	−	NoC	+	−
P66-His_12_	NoC	NoC	NoC	NoC	NoC
PelB-BB0238	nd	nd	nd	+	nd
PelB-P66	nd	nd	nd	+	nd

**Table 2:** *E. coli* expression strains used in this study.

Strain	Genotype	Source/Reference
BL21(DE3)	F^−^ *ompT hsdS*_*B*_ (r_B_^−^ m_B_^−^) *gal dcm* λDE3[P_*lacUV5*_- *T7RNAP*]	Invitrogen #C6000-03
C43(DE3)	BL21(DE3); uncharacterized mutations described in^102^	Lucigen #60446-1
Lemo21(DE3)	BL21(DE3); pLemo[pACYC184-P_*rhaBAD*_-*lysY*, chloramphenicol^R^]	New England Biolabs #C2528H
KTD101	Δ*tig100* Δ(*araD*-*araB*)*567* Δ*lacZ4787*(::*rrnB-3*) *lacI*^*P*^*-4000*(*lacI*^*Q*^ or *lacI*^+^ ^108^) *rph-1* Δ(*rhaD*-*rhaB*)*568*, *hsdR514*	^104^
KTD101(DE3)	KTD101; λDE3[P_*lacUV5*_- *T7RNAP*]	This work
BL21-AI	F^−^ *ompT hsdS*_*B*_ (r_B_^−^ m_B_^−^) *gal dcm araB*::*T7RNAP*-*tetA* (tetracycline^R^)	Invitrogen #C6070-03

Positive expression for the His_12_ constructs targets was observed primarily in BL21-AI, although BBA57-His_12_ was expressed in several strains (Table 1). Expression in BL21-AI was obtained for five targets: HtrA-His_12_ (Fig. 1c, lane 4; and Fig. 1d, lanes 19 and 20; expected 51.4 kDa, observed ~52 kDa), BBA57-His_12_ (Fig. 1c, lane 6; expected 47.5 kDa, observed ~55 kDa), BB0323-His_12_ (Fig. 1c, lane 7; expected 44.5 kDa, observed ~45 kDa), BB0238-His_12_ (Fig. 1e, lane 27; expected 29.7 kDa, observed ~31 kDa) and DipA-His_12_ (Fig. 1e, lane 29; expected 39.0 kDa, observed ~37 kDa). Of these, BBA57-His_12_ ran farthest from its expected size. The expected size of mature BBA57-His_12_ (i.e., BBA57-His_12_ lacking the signal peptide due to processing at the inner membrane) is 47.5 kDa. Here BBA57-His_12_ migrated at ~55 kDa (Fig. 1c, lane 6), which importantly reproduces the results done in *Bb* in a previous study^54^ that observed BBA57-His_6_ migrating in a Western blot at 56 kDa. The finding that membrane proteins migrate differently from the expected size is not unusual due to incomplete denaturation by detergents^55^. For BBA57-His_12_, we also observed a minor band at ~110 kDa (Fig. 1c, lane 6), which suggests a possible dimer that is partially stable in sodium dodecyl sulfate (SDS). That both bands in Fig. 1c, lane 6, represent BBA57 was supported by absence of these bands in lane 5 (Fig. 1c), which contains a duplicate culture but lacked the inducer arabinose.

Negative expression results are shown in Supplementary Figs. S5a–c and S6a,b, and S7. No expression was apparent at any time for the three targets RevA, BB0405 and P13. RevA-His_12_ lacked visible expression (Fig. 1c, lane 3; Supplementary Fig. S5b, lanes 15 and 16). BB0405-His_12_ was not visible (Fig. 1c, lane 8; Supplementary Fig. S5b, lanes 5–13; Supplementary Fig. S6a, lanes 10–11; Supplementary Fig. S6b, lane 12). Also, P13-His_12_ was not visible (Fig. 1c, lanes 10 and 11; Supplementary Fig. S5a, lanes 2–8; Supplementary Fig. S6a, lanes 8–9; Supplementary Fig. S6b, lane 10). Expression in Lemo(DE3) did not yield visible targets, as was tested only for BB0405-His_12_, DipA-His_12_ and BBA57-His_12_ (Supplementary Fig. S7, lanes 2–10). Also, there was no evidence of secretion into the culture supernatant for those samples that were tested (Fig. 1e, lanes 24, 26 and 28; and Supplementary Fig S6b, lanes 9, 11, 13, and 15). Of these, where the target was visible in the unharvested culture (BB0238-His_12_ in Fig. 1e, lane 27; and DipA-His_12_ in Fig. 1e, lane 29), these results show that these targets were associated with the cells and were not secreted (compare to Fig. 1e, lane 28; and Supplementary Fig. S6b, lane 9).

Expression of some targets was obtained only upon analyzing various expression conditions. Interestingly, the four targets HtrA-His_12_, BB0238-His_12_, BB0323-His_12_, and DipA-His_12_ could only be expressed in BL21-AI. Negative results in other strains are in Fig. 1c, lane 11; Supplementary Figure S5a, lanes 9–14; Supplementary Figure S5b, lane 2; and Supplementary Fig. S5c, lanes 10–16. BBA57-His_12_ was the most widely expressed target, and was the only target that could be expressed in strains other than BL21-AI (Table 1). BBA57-His_12_ was observed in BL21(DE3) (Supplementary Fig. S5c, lanes 5–6) and KTD101(DE3) (Supplementary Fig. S5c, lanes 7–8). Even in BL21-AI, varying the induction conditions could eliminate expression: DipA-His_12_ was visible only at 18 °C (Fig. 1e, lane 29) but not 37 °C (Supplementary Fig. S5b, lanes 3–4). When minimal medium was used, neither BBA57-His_12_ nor DipA-His_12_ were visible in BL21-AI (Supplementary Fig. S6a, lanes 12–15; this condition includes a shift from 37 °C to 25 °C at induction, plus incubation for 14 h or 22 h), while HtrA-His_12_ continued to be expressed at this condition (Fig. 1d, lanes 19–20).

### Expression in *E. coli* of *B. burgdorferi* BB0238 and P66 was achieved using the N-terminal PelB signal peptide and maltose binding protein tag

Because several targets lacked visible expression in *E. coli* (Table 1), we pursued expression driven by an alternative N-terminal membrane targeting sequence (Fig. 1b) containing the powerful PelB signal peptide from *Erwinia carotovora*^56^ plus the stabilizing MBP sequence^57^. In this paper, these proteins are referred to as “PelB-target”. A similar PelB-MBP expression construct supported crystal structure determination of cyanobacterial and *Erwinia* ion channels by successfully directing high levels of expression to the *E. coli* membrane^58^. The PelB signal peptide also successfully directed other spirochetal OM proteins to the *E. coli* OM, like the *Treponema* β-barrel porins TprC, TprI, and MOSP^59^. With the PelB-MBP sequence, we observed expression in *E. coli* of a sixth *Bb* target, PelB-P66 (Fig. 1d, lanes 15–16; expected 110 kDa, observed ~115 kDa), in addition to expression of PelB-BB0238 (Fig. 1d, lanes 17–18; expected 71.3 kDa, observed ~68 kDa). Both targets had higher protein levels at 22 h post-induction (Fig. 1d, lanes 16 and 18) compared to 14 h (Fig. 1d, lanes 15 and 17). Expression of PelB-P66 also depended on expression conditions, since PelB-P66 and PelB-BB0238 were not visible in BL21-AI after overnight growth in LB at 18 °C (Fig. 1e, lanes 23–26).

### BBA57-His_12_ is translocated to the *E. coli* membrane from which it is soluble in non-ionic and zwitterionic detergents

We focused on BBA57-His_12_ after we identified this protein in the detergent soluble fraction (1% DDM) that was derived from total cellular protein of BL21-AI (Supplementary Fig. S8, lane 2) when we applied a rapid screen (Supplementary Methods) to the small scale expression cultures from Fig. 1c and Supplementary Fig. S5c. In contrast to this positive result in the BL21-AI strain (Supplementary Fig. S8, lane 2), strains KTD101(DE3) and BL21(DE3) did not yield visible detergent soluble BBA57-His_12_ (Supplementary Fig. S8, lanes 3 and 4). From this rapid screen, BBA57-His_12_ was also the only expressed target from Fig. 1c that was detergent soluble. For example, HtrA-His_12_ and BB0323-His_12_ that were visible in total protein (Fig. 1c, lanes 4 and 7) were not visible in the 1% DDM fraction (Supplementary Fig. S8, lanes 5 and 6).

**Figure 2:**
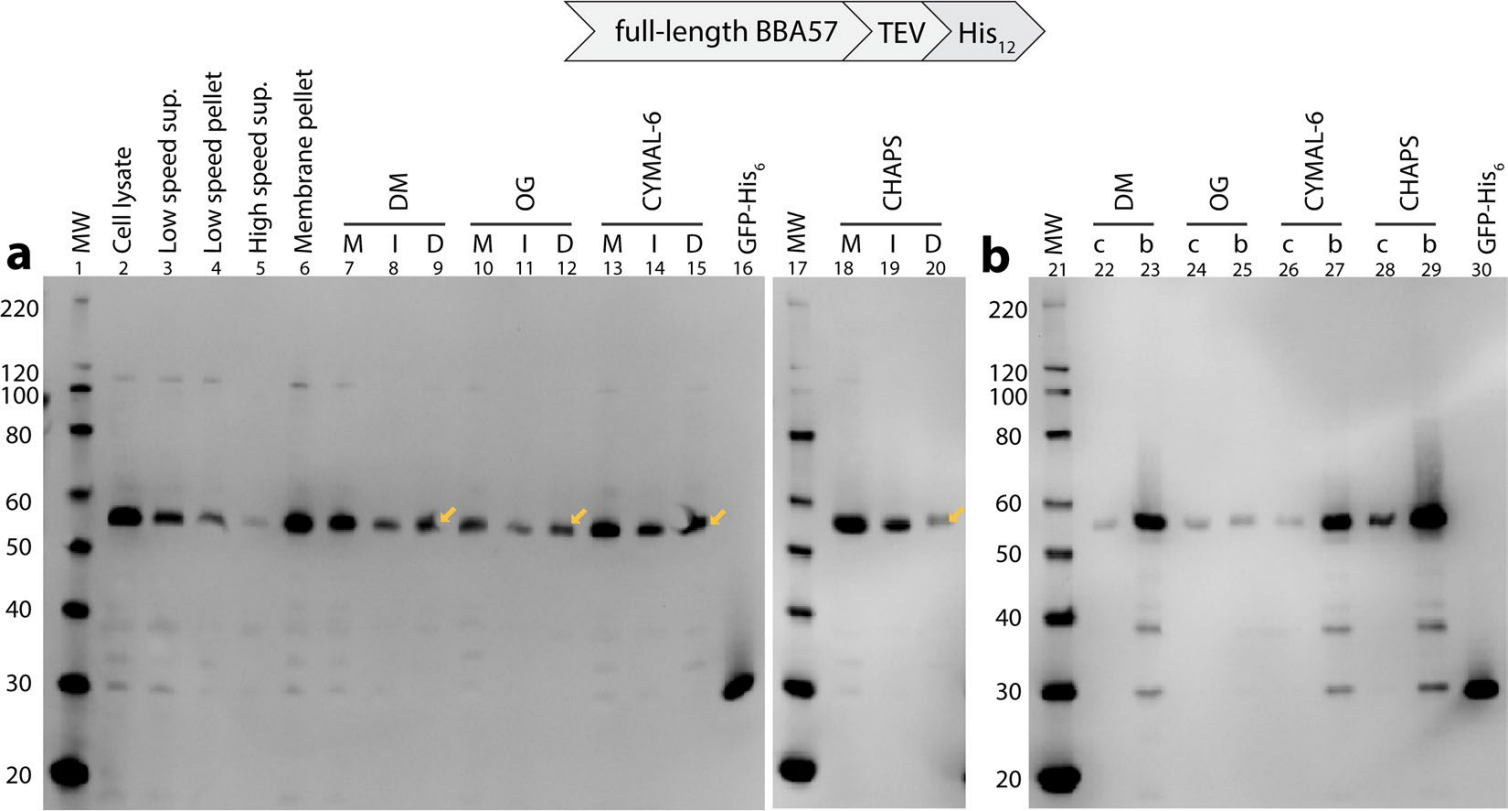
Full-length, C-terminally His-tagged BBA57 is solubilized by detergents from a cell membrane fraction of *E. coli* and can be purified by nickel affinity. Shown are Western immunoblots (anti-His). (**a**) Fractions from a cell membrane preparation, and 2% detergent extraction with BBA57-solubilizing detergents. M, membrane pellet fraction, which represents one suspended aliquot from lane 6. I, detergent-insoluble portion of the membrane fraction. D, detergent-soluble portion of the membrane fraction (yellow arrows). Lanes M, I, and D all contain 47 µL culture equivalent per lane. (**b**) Purification by nickel affinity of the detergent-soluble portions of the membrane fraction by two methods. Lanes c, Ni-NTA silica spin columns (93 µL culture equivalent per lane). Lanes b, nickel sepharose magnetic beads (469 µL). Purified GFP-His_6_ (lanes 16, 30) was used as a Western positive control. Abbreviations: MW, molecular weight; sup., supernatant.

Importantly, we verified that BBA57-His_12_ was in the cellular membrane fraction of *E. coli* by performing detergent extractions of the membrane fraction isolated as described here. Figure 2a shows the results of detergent extraction experiments that are detailed in the Methods. The full detergent screen for Fig. 2 is shown in Supplementary Fig. S9. We were able to partially solubilize BBA57-His_12_ from the *E. coli* membrane using four detergents. An anti-His Western blot identified that BBA57-His_12_ was present mainly in the cellular membrane fraction (Fig. 2a, lane 6). Approximately half of the BBA57-His_12_ from the membrane fraction was solubilized in the detergents DM (Fig. 2a, lane 9), OG (Fig. 2a, lane 12), and CYMAL-6 (Fig. 2a, lane 15), and partially solubilized in CHAPS (Fig. 2a, lane 20).

### BBA57-His_12_ was isolated by metal affinity chromatography from a detergent-solubilized membrane fraction

To verify metal-binding, the detergent-solubilized protein fractions in Fig. 2a were tested for binding to two different nickel affinity formats. BBA57 that was solubilized in DM, OG, CYMAL-6, and CHAPS bound to both nickel silica spin columns (Fig. 2b, lanes 22, 24, 26 and 28) and nickel sepharose magnetic beads (Fig. 2b, lanes 23, 25, 27 and 29). Comparing detergents, the highest yield was obtained in DM, CYMAL-6 and CHAPS (Fig. 2b, lanes 23, 27 and 29) compared to OG (lane 25). Also, we noted that the ~110 kDa ‘dimer’ of BBA57-His_12_ (Fig. 2a) diminished throughout the membrane/detergent purification, and was no longer visible throughout in the nickel purification samples (Fig. 2b). Despite any differences in yields, it will be important to also determine the quality and the membrane localization of the protein prior to structure determination. Nevertheless, nickel affinity will be a useful first purification step.

### PelB-P66 is not translocated to the *E. coli* membrane and is not detergent-soluble

**Figure 3:**
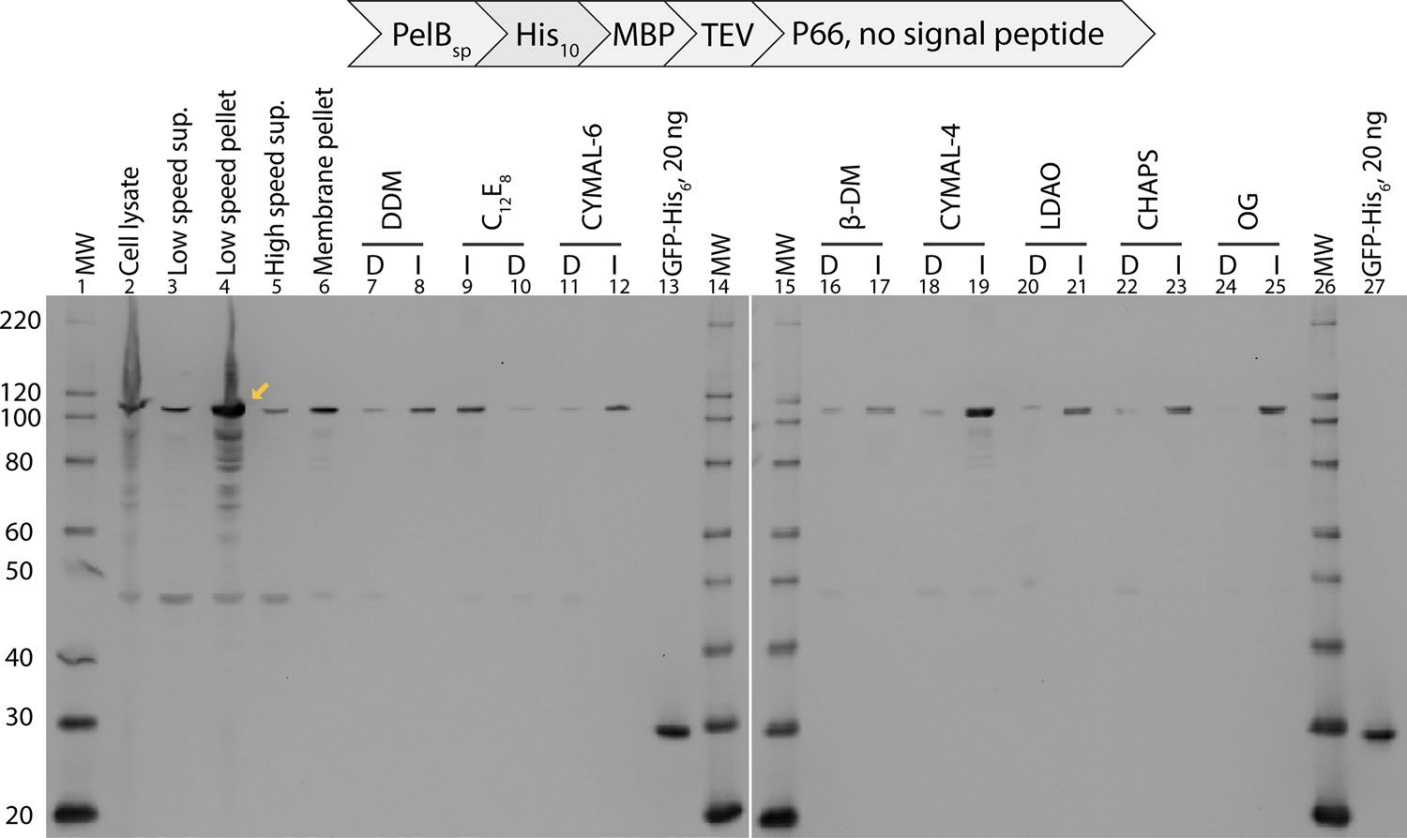
PelB-P66 is not solubilized by detergents from the *E. coli* cell membrane fraction. Shown are anti-His-tag Western immunoblots. Each lane contains sample from 23.4 µL of culture. I, detergent-insoluble portion of the membrane fraction. D, detergent-soluble portion of the membrane fraction. Most of PelB-P66 (110 kDa) is not membrane localized (yellow arrow). Purified GFP-His_6_ was used as a Western positive control. Abbreviations: MW, molecular weight marker.

We also determined whether the PelB-P66 that was expressed (Fig. 1d, lane 16) was also membrane-localized. PelB-BB0238 was not analyzed due to low expression levels (Fig. 1d, lanes 17–18). We evaluated expression of PelB-P66 in BL21-AI under different inducer concentrations, induction times and temperatures. Immunoblots of total protein showed increased expression with increasing concentrations of arabinose in the presence of IPTG (Supplementary Fig. S10a,b). IPTG was necessary to remove the bound LacI protein, which is also encoded in this vector, from the *lacO* sequence in the vector’s T7*lac* promoter. To determine membrane localization of PelB-P66, a membrane fraction was isolated (Fig. 3, lanes 2–6) from a culture grown at the same conditions as Supplementary Fig. S10a (0.001% arabinose, 8 h expression) and then extracted with several nonionic and zwitterionic detergents (Fig. 3, lanes 7–12, 16–25). In the low speed centrifugation of cell lysate, most of the PelB-P66 was found in the pellet (Fig. 3, lane 4), suggesting that a significant fraction of the target was in inclusion bodies. Upon subjecting the low speed supernatant (Fig. 3, lane 3) to the high speed centrifugation, most PelB-P66 was in the membrane fraction (Fig. 3, lane 6) rather than the soluble fraction (Fig. 3, lane 5). Upon extraction of the membrane fraction with several detergents, Western blot analysis showed that the majority of PelB-P66 was in the detergent insoluble fraction (Fig. 3, lanes 8, 9, 12, 17, 19, 21, 23, 25).

### BBA57-His_12_ is translocated to the *E. coli* outer membrane, detergent solubilized, and purified by nickel affinity

**Figure 4:**
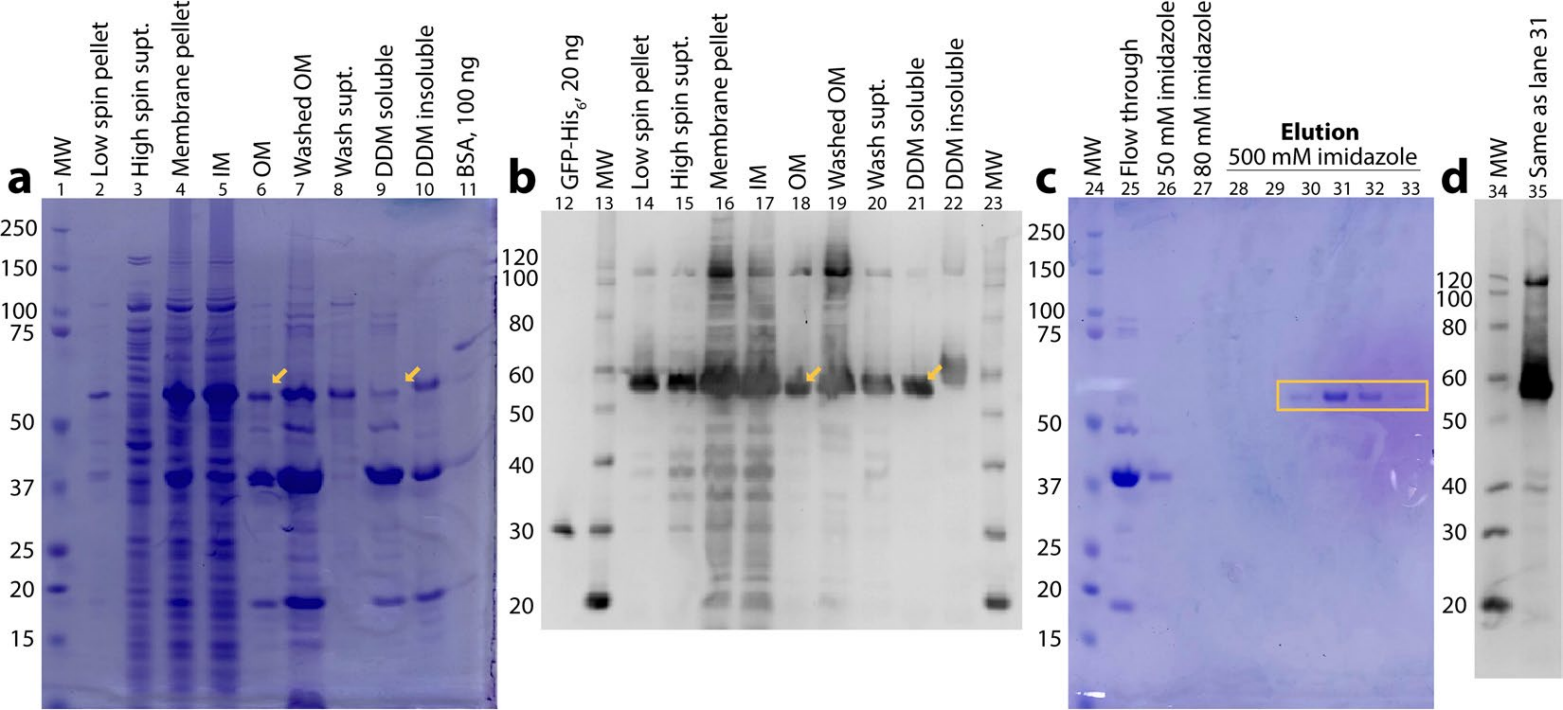
Full-length, C-terminally His-tagged BBA57 is isolated from the outer membrane fraction of *E. coli*, solubilized by detergent, and purified by affinity chromatography. Shown are Coomassie blue stained SDS-PAGE (**a,c**) and anti-His Western immunoblots (**b,d**). (**a,b**) Fractions from a cell membrane preparation showing BBA57 localized in the OM and extracted with 2% DDM (yellow arrows). Purified BSA and GFP-His_6_ were used as positive controls. (**c,d**) Detergent-solubilized, OM BBA57 purified by nickel affinity (boxed lanes). Lanes 28–33 and 35 contain 7 µL of each elution fraction. Abbreviations: BSA, bovine serum albumin; MW, molecular weight marker; IM, inner membrane; OM, outer membrane; supt., supernatant.

Translocation of BBA57-His_12_ to the OM fraction of *E. coli* was revealed after incubating the cell membrane fraction with N-lauroyl sarcosine, which solubilizes the inner membrane and thus allows isolation of an OM fraction. The cell membrane fraction is shown by Coomassie-stained SDS-PAGE in Fig. 4a, lane 4, and by Western blot in Fig. 4b, lane 16. The inner membrane fraction is shown in Fig. 4a, lane 5, and Fig. 4b, lane 17. The Coomassie stain indicates that the OM (i.e., the pellet from the sarkosyl solubilization) contained approximately a quarter of the total BBA57-His_12_ (Fig. 4a, lane 6) that had been in the membrane pellet (Fig. 4a, lane 4), while the other three-quarters was in the inner membrane (i.e., the sarkosyl soluble supernatant; Fig. 4a, lane 5). The OM fraction (Fig. 4a, lane 6; Fig. 4b, lane 18) was then solubilized by DDM detergent (Fig. 4a, lane 9; Fig. 4b, lane 21). Coomassie stain showed that approximately half of the BBA57-His_12_ that was in the in the OM (Fig. 4a, lane 6) was solubilized in 2% DDM (Fig. 4a, lane 9). The BBA57-His_12_ in the form of a DDM-protein detergent micelle was then purified using nickel affinity chromatography. The results are in Fig. 4c, which shows that we were able to isolate pure BBA57-His_12_ (Fig. 4c, lanes 30–33). Less than 10% of BBA57-His_12_ did not bind to the nickel as was visible in the column flowthrough (Fig. 4c, lane 25), and no BBA57-His_12_ was visible in the wash steps at 50 and 80 mM imidazole (Fig. 4c, lanes 26–27). BBA57-His_12_ that was eluted from the column with high imidazole (Fig. 4c, lane 31) was verified by anti-His Western (Fig. 4d, lane 35) and by LC/MS/MS following trypsin digestion, which identified 85 peptides (461 peptide spectrum matches) representing 89% coverage of the BBA57-His_12_ sequence. The yield in Fig. 4c was 0.28 mg of purified BBA57-His_12_ per liter of *E. coli* culture.

### BBA57-His_12_ isolated from the outer membrane forms a large homo-multimeric complex composed mainly of α-helices

**Figure 5:**
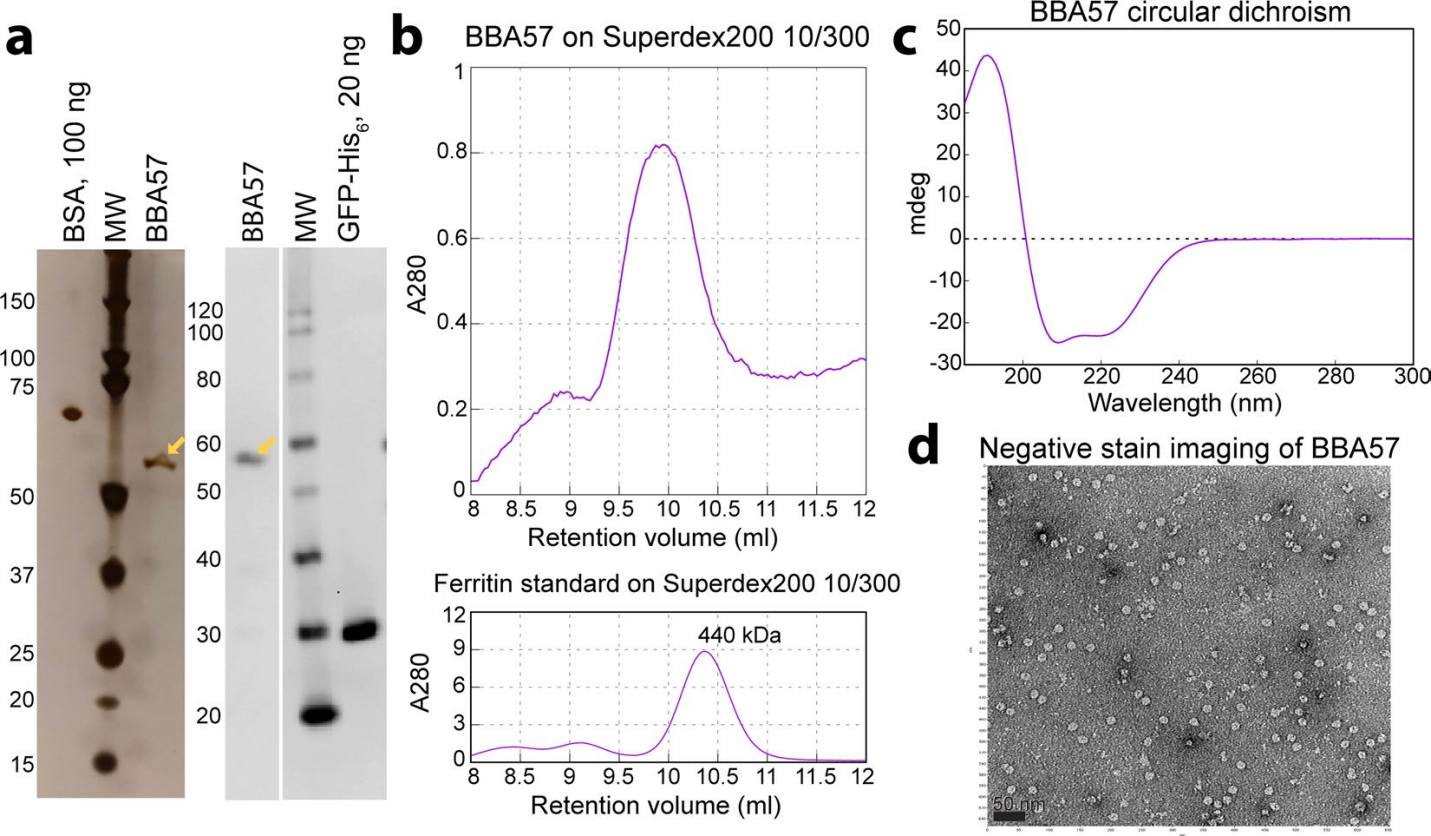
Full-length, C-terminally His-tagged BBA57 forms a large homo-multimeric complex composed mainly of α-helices. (**a**) Silver stained SDS-PAGE and and anti-His Western immunoblot from (**b**) a 280 nm absorbance peak (9.5–10.5 mL) obtained on size exclusion chromatography following nickel affinity purification. Also shown is the peak from a ferritin molecular weight standard run on this column. The BBA57 peak was subjected to (**c**) circular dichroism and (**d**) negative stain electron microscopy. The length of the black bar is 50 nm. Abbreviations: BSA, bovine serum albumin; MW, molecular weight marker.

BBA57-His_12_ that was isolated from the outer membrane and purified by nickel affinity was further purified by size exclusion chromatography. Silver stained SDS-PAGE (Fig. 5a) showed that a highly pure band migrating like BBA57-His_12_, which was verified by anti-His immunoblot (Fig. 5a), was present in a size exclusion peak (Fig. 5b) that migrated near the 440 kDa ferritin standard (Fig. 5b). Circular dichroism of this BBA57 peak (Fig. 5c) indicated structural features of 69.0% (±5.7%) α-helices, 2.5% (±1.7%) β-strands, 10.4% (±3.4%) turns, and 18.7% (±5.7%) unordered. Negative stain (Fig. 5d) gave evidence of uniformly sized particles with a diameter of approximately 10 nm.

## Discussion

In this study, we endeavored to heterologously express in *E. coli* several *Bb* virulence determinants with their signal peptides, thus pursuing the pathways that fold, process, and multimerize membrane-translocated proteins^60^. These pathways include the inner membrane translocases, primarily SecY^61^, that recognize, fold and move α-helices laterally into the membrane bilayer^62^. At the inner membrane, signal peptides are removed after translocation; lipoproteins are processed by the Lol system^30,63^; periplasmic chaperones like Skp and DegP maintain proteins in an unfolded state, refold protein (peptidyl-prolyl isomerases), or form disulfide bonds (disulfide oxidoreductases)^64^; and OM β-barrels are assembled, possibly one β-strand at a time, by the β-barrel assembly machine^60^ and may also form multimers^65^. These pathways are moderately conserved from *E. coli* to *Bb*^32,66–68^, making *E. coli* a reasonable starting system for heterologous overexpression of membrane-translocated proteins. In fact, surface expression in *E. coli* has been reported for full-length forms of *Bb* OspA, P66 and OspB^23,69,70^. That BBA57-His_12_ was localized in the *E. coli* OM fraction (Fig. 4a, lane 6) using conventional sarkosyl solubilization of the inner membrane^71^ suggests that BBA57 was sent through the proper membrane folding and processing pathways.

Importantly, the purified, membrane-translocated BBA57 showed evidence of multimerization (Fig. 5d). The approximate particle diameter of 10 nm (Fig. 5d) is consistent with its migration on size exclusion (Fig. 5b) and is similar to the diameters of the multimeric structures of Wza (10.5 nm)^41^ and CsgG (12 nm)^42^. The α-helical nature of BBA57 is reminiscent of the *E. coli* capsule-forming Wza homo-multimer that secretes polysaccharides. It is possible that multimerization of BBA57 also requires lipidation and thus membrane-translocation, as is the case for Wza^43^ and CsgG^42^. There is currently published evidence for a second α-helical homo-oligomer in *Bb*. Porin-active P13 forms a ~300 kDa homo-oligomer^72^ with a predicted α-helical structure^73^. Unlike for Wza, the membrane-spanning helices in P13 were sufficiently hydrophobic to be identified^73^ using programs, HMMTOP^74^, TMHMM^75^, and PSORT^76^ that identify inner membrane α-helices.

In this study, eleven target proteins were selected (Supplementary Table S2) in order to support future structural studies of their membrane directed forms. The approximate number of targets was based on the proportion of successful membrane-translocation in our previous work with targets from another tick-borne pathogen, *Francisella tularensis*^77^. The *Bb* targets were identified from the published literature and chosen based on some or all of the following criteria. (**1**) All of the targets have a demonstrated role in pathogenesis (Supplementary Table S2). Mutation of nine of the targets reduces pathogenesis (Supplementary Table S2). For the remaining two targets, P13 is a dominant component of the multiprotein complexes in the *Bb* OM, but its role in these complexes is unclear^38^; and BB0406^28^ has demonstrated porin activity and is coexpressed with BB0405^26^, with which it has 46% sequence identity. (**2**) All targets are associated with the inner or outer membrane (Supplementary Table S2). Three of the targets are predicted lipoproteins: BBA57^51^, RevA^54^ and BB0323^54^. However, actual lipidation and the type of lipid have yet to be demonstrated. (**3**) The targets are unique to *Borrelia* species. Sequence searches using BLASTp were used to identify the absence of homologous structures in the Protein Data Bank (PDB)^33^. Only the High Temperature Requirement A (HtrA) protein has homologous structures in the PDB, all of which are from non-*Borrelia* species, and which contain less than 35% sequence identity. Functions and substrates of Htr proteins vary remarkably between species’ forms despite similar structures, and are often unique virulence factors in pathogens, making them attractive targets^78^. In general, the selected targets are also highly conserved across the genus *Borrelia*, making the results of this work possibly applicable to multiple *Borrelia* species. (**4**) Another criterion was the lack of structures in the PDB. RevA has a structure in the PDB^33^, a 1.80 Å crystal structure (accession no. 5EQZ, unpublished [J.K. Yano, A.H. Sullivan, J. Abendroth, D.D. Lorimer, and T.E. Edwards]), which is of the soluble domain that was expressed in the cytoplasm of *E. coli*. Recently the crystal structure of the N-terminal 27 kDa fragment of BB0323^79^ was solved at 2.35 Å, following its expression in the *E. coli* cytoplasm and also lacking the signal peptide. This effort revealed structural homology to the spectrin superfamily, members of which function as linkers in bacterial cell division and the eukaryotic cytoskeleton^80^. Importantly, for all other membrane-translocated proteins from *Borrelia* spp., the available structures in the PDB have also been solved for only the soluble domain (Supplementary Table S1): each of these proteins was expressed without a signal peptide and therefore was not directed to the membrane or to the membrane protein folding pathways. For the other targets in Supplementary Table S2, no atomic-resolution structures are available. The β-barrel fold that is expected in BB0405, BB0406, DipA and P66 is based on sequence predictions and porin activity^26,29,36^, but no experimental structure has been determined. (**5**) Finally, the lack of experimental structures will be important to unravel the function on a molecular basis of many of the eleven targets. RevA, Lmp1 and P66 act as adhesins^10,24,81^. However, molecular functional activities have not been determined for BBA57, BB0238 or BB0323, and the functional roles of the porin activities are unknown for P13, BB0405, BB0406 and P66. Future structures will better illuminate how these important proteins function in *Borrelia* species’ pathogenesis.

Of the targets expressed, two were analyzed for membrane localization. BBA57-His_12_ was found in the *E. coli* membrane, while PelB-P66 was not. There are several possible contributing factors for lack of membrane localization. One is interference with membrane protein processing by the presence of even small purification tags, as has been demonstrated^82,83^. This interference could be overcome in the future with tag-free purification from the relatively pure OM fraction (Fig. 4a, lane 6), possibly aided by antibodies^51^. Other factors include differences between *E. coli* and *Bb* in the membrane protein processing components^31,84^. Additionally, *Bb* is unable to synthesize fatty acids, and instead, exchanges lipids with its eukaryotic host, resulting in a lipid composition reflective of the surrounding host tissue^85^. The *Borrelia* species also lacks lipopolysaccharides and is one of the few prokaryotes that contains cholesterol and cholesterol glycolipids^84,86,87^. Specifically, cholesterol located in the OM of *Borrelia* species may assist in formation of tightly packed lipid microdomains (lipid rafts), which are known to play roles in protein sorting and receptor signaling^88^. Also, some targets are processed by *Bb* specific proteases. In *Bb*, the HtrA protease processes BB0323^79^, P66^89^, and Lmp1^90^. Lmp1 is processed into three separate domains (N-terminal, middle, and C-terminal) that confer unique pathogenic properties^90^. Lmp1 constructs could not be generated (Table 1), possibly due to instability at the DNA level of the expression-optimized DNA. The C-terminal 28 residues^73^ of P13 are removed by the CtpA C-terminal protease^91^. BB0323 undergoes complex processing: BB0323 is proteolyzed by HtrA to yield a C-terminal ~15 kDa fragment^79^ plus an N-terminal 27 kDa fragment^79^ that interacts with BB0238^92,93^ and forms a 48.5 kDa dimer^93^. BB0323 is also proteolyzed by CtpA^91^. Here we showed recombinant expression in *E. coli* BL21-AI of the unprocessed size of BB0323 (Fig. 1c, lane 7) but could not extract BB0323 from total cellular protein using 1% DDM (Supplementary Fig. S8, lane 6).

It is possible that additional targets can be solubilized with somewhat higher detergent concentrations, such as 1.5–2%, which have been used in structure determination of other OM proteins^94,95^. Previously, 2% LDAO was used to solubilize natively folded P66 from the *Bb* OM^96^. Although PelB-P66 could not be detergent-solubilized under the conditions explored, use of a harsher detergent extraction may lead to solubilized protein. However, we did not explore these conditions as harsh detergent treatment often leads to protein that is only partially folded and therefore not suitable for structure determination.

Surprisingly, among the tested targets, expression of *Bb* proteins was highly strain-dependent (Table 1). Expression of the selected *Bb* targets was most successful in *E. coli* strain BL21-AI (Fig. 1c–e; Table 1). BL21-AI is a derivative of the BL21(DE3) line, with more tightly regulated expression through the *araBAD* promoter (P_BAD_), which controls expression of T7 RNA polymerase and reduces the likelihood of leaky expression in uninduced cells^97^. Leaky expression of proteins toxic to *E. coli* could kill the bacterium before it would be able to form colonies. The inability of some transformed strains to form colonies suggests that many of these membrane-translocated targets may be toxic to the *E. coli* strains listed in Table 1, possibly due to inadvertent disruption of the inner membrane. Interestingly, BBA57-His_12_ was the only target to express in more than one of the tried strains (Table 1), but was detergent-soluble only when expressed in BL21-AI cells. P66-His_12_ was especially toxic to the cells, yielding no colonies in any of the *E. coli* strains (Table 1). Expression of P66 was successful only after changing the construct to include the PelB signal sequence fused with MBP (Table 1). The BB0406-His_12_ expression construct may also be unstable as this clone could not be made (Table 1).

*E. coli* is often the go-to system for structural biology of bacterial membrane proteins, some methods of which require near-milligram amounts of purified protein. Further improvements in expression of some of the targets here may be achieved by systematically varying the culture medium, temperature, induction time and inducer concentration, as was suggested upon obtaining positive expression for HtrA-His_12_ (Fig. 1c, lane 4; Fig. 1d, lanes 19–20), BB0238-His_12_ (Fig. 1e, lane 27), BB0323-His_12_ (Fig. 1c, lane 7) and DipA-His_12_ (Fig. 1e, lane 29). These approaches may be promising for the remaining samples in Supplementary Fig. S10. Adding the PelB-MBP sequence to P66 (Fig. 1d, lanes 15–16) supports the approach of using alternative N-terminal membrane targeting sequences. In fact, a previous study reported integration of P66 in the *E. coli* OM when the *E. coli* OmpT signal peptide was used, although expression was also reported as inefficient^23^. Future studies will assess whether the PelB sequence without MBP is sufficient for expressing P66 or other difficult expression targets, and if these proteins are translocated to the membrane. While it is possible that alternate constructs, strains and expression conditions could direct expression of some of these targets to the *E. coli* membrane, the results described in this paper, plus the importance of processing pathways and unique lipids to proper folding of transmembrane proteins, suggest the necessity to develop overexpression systems within non-virulent strains of *Borrelia* to allow structural studies of more membrane-translocated *Bb* proteins. High expression levels in *Bb* will be facilitated by the developed genetics systems and the availability of strong, inducible promoters (reviewed in^98^).

It is important to note that the rapid screen used in Supplementary Fig. S8 (lane 2) to identify detergent soluble BBA57-His_12_ does not prove that BBA57 was integrated into the cell membrane, because the source of the extracted protein included soluble cytoplasmic and periplasmic proteins. Therefore we had addressed this issue by first isolating the membrane fractions and then extracting protein from these isolated membrane fractions. That BBA57-His_12_ was associated with the *E. coli* membranes was indicated by the presence of BBA57-His_12_ in the pellet of a high-speed centrifugation of cellular fragments (Fig. 2a, lane 6) and in the pellet of a sarkosyl solubilization (Fig. 4a, lane 6). The sarkosyl solubilization and nickel affinity purification in Fig. 2b,c strongly suggests that recombinant BBA57 is associated with the OM and can be purified at a level sufficient for structural analyses.

Other groups have characterized BBA57 subcellular localization in *Bb*. Native BBA57 was shown to be OM-localized using either proteinase K digestion of whole cells or isolation of the OM with hypotonic citrate buffer plus sucrose gradient^47^. One study also used the citrate/sucrose method^99^ to localize C-terminally His_6_-tagged BBA57 to the *Bb* OM^54^, while another study^51^ found BBA57 in the insoluble fraction of whole cell lysates after extraction with Triton X-114. Structural information on BBA57 is expected to illuminate these results.

The BBA57 sequence does not suggest an obvious structure. It contains a single conserved protein domain (pfam05058; residues 52–141 of GenBank accession number AAC66270.1) that has 29% identity to the ActA actin assembly inducing protein, a cell surface virulence factor from the Gram-positive *Listeria monocytogenes*. ActA is implicated in biofilm formation and dissemination via actin polymerization^100^, but like BBA57, there are no structures determined yet for this protein. BBA57 has a relatively low hydrophobicity of 27% and is not predicted (Supplementary Table S2, column 6) to form transmembrane α-helices or a β-barrel, using the standard programs that rely on high hydrophobicity or monomeric β-barrels. Secondary structure predictions that are not based on a membrane environment (e.g., PSIPRED) suggest that the protein would contain 50% α-helices, which is consistent with the results in Fig. 5c. Unlike inner membrane α-helices, OM α-helices are often amphipathic, where the hydrophobic side of the α-helices face the membrane, and the hydrophilic amino acids align along the inner structure. Two α-helical OM proteins have so far been demonstrated structurally using protein directed to the membrane: the polysaccharide translocon Wza^41^ and the phospholipid translocon MlaA^44^. The homo-multimeric OM β-barrel structures determined for CsgG, Hia and TolC also cannot be predicted from their primary protein sequence^42,45,46^. These fascinating proteins, that function in immune evasion, lipid transport, biofilm formation, adhesion, and drug resistance, serve as powerful examples for future structural studies of membrane translocated virulence proteins in the Lyme disease pathogen.

This work is the first reported purification of membrane-translocated BBA57, the first evidence of its localization in the *E. coli* membrane, and the first evidence of possible multimerization. BBA57-His_12_ was solubilized in several different detergents, including DDM and OG, and reproducibly purified by affinity chromatography, indicating its potential as a future structural target. Future studies will focus on functional and structural studies of the purified recombinant BBA57-His_12_ that will complement *in vivo* studies. We suggest that its detailed structure may indicate that BBA57 functions as an assembly factor, chaperone, or scaffold. Availability of the lipidated forms of these purified virulence determinants will also allow novel vaccination studies: it has been reported that lipidated OspA yields a greater immune response than non-lipidated^101^. Unfortunately, this study omitted details on preparation of the lipidated OspA. The results described here also highlight the non-trivial technical hurdles required to screen membrane fractions in order to begin to identify proper membrane protein folding and processing, and perhaps provide some justification for the lack of membrane-integral structures from *Borrelia* species. As structure determination methods continually improve, as recently seen with femtosecond nanocrystallography and single particle cryoelectron microscopy, protein yields will likely become much less important than protein quality, such that even modest improvements in yields will result in important new structures. This work contains significant first steps towards what we expect will be the first structure determination of a membrane-translocated form of any protein from *Borrelia* species.

## Methods

### Generation of expression constructs

Sequence analysis methods are in the Supplementary Methods. To obtain significant expression levels, we obtained genes optimized for expression in *E. coli* from GenScript. To minimize cost, the genes were obtained as tandem sequences on two plasmids (Supplementary Fig. S1) that were used as PCR template DNA for the expression subclones. The optimized DNA sequences are provided in Supplementary Fig. S2a,b. The genes were provided on plasmids pUC57-BBclone1 and pCC1-4k-BBclone2, containing 7 and 4 genes, respectively (Supplementary Fig. S1). The latter plasmid has optimized genes for BB0323, P13, DipA and Lmp1. Because cloning into pUC57 was problematic for this plasmid, these optimized genes may contain DNA sequence that is unstable in the high copy pUC57 plasmid (>100 plasmids/cell). Unlike pUC57, pCC1-4k-BBclone2 has a low copy RK2 origin of replication (<10 plasmids/cell).

Each of the 11 targets was cloned into the pRSET-TEV-12His vector that allows T7 promoter based expression. The C-terminal sequence ENLYFQ*GHHHHHHHHHHHH allows removal of the His_12_ tag by cleavage (*) with tobacco etch virus (TEV) protease (Fig. 1a). Long polyhistidine tags are often necessary for efficient binding of membrane proteins to the nickel affinity column during metal affinity purification. The complete DNA and protein sequences for the BBA57-His_12_ construct are provided as an example in Supplementary Fig. S3a,b. Two *Bb* targets, Lmp1 and BB0406, could not be cloned into pRSET-TEV-12His (Table 1). Because Lmp1 was also present in the low copy pCC1-4k-BBclone2, it is possible that the expression-optimized Lmp1 gene may contain a DNA sequence that is unstable in the high copy pRSET and pUC57 vectors.

Because some targets lacked visible expression under any condition, select targets were additionally cloned into the novel pelB-MBP vector, which replaces the *Bb* signal peptide with the PelB signal peptide, followed by a His_10_ tag, the mature maltose binding protein (MBP), and a TEV protease cleavage site designed to generate the native N-terminus of the mature target protein (Fig. 1b). The complete DNA and protein sequences for PelB-P66 are provided in Supplementary Fig. S4a,b.

### Cloning and transformation

Plasmids in this paper are listed in Supplementary Table S3. Genes optimized for expression in *E. coli* were acquired from GenScript. This optimization includes codon usage bias, GC content, CpG dinucleotides content, mRNA secondary structure, internal ribosomal binding sites, and repeat sequences. Sequences for all of the optimized genes are in Supplementary Fig. S2a,b. Ligation-independent cloning was accomplished as described previously^77^ and using the *E. coli* Stellar cloning strain (Clontech # 636766). Plasmid bearing cells were selected on LB Miller agar plates with the appropriate antibiotic (50 µg/mL carbenicillin, 30 µg/mL kanamycin, and/or 25 µg/mL chloramphenicol, according to Table 2 and Supplementary Table S3) with incubation at 37 °C overnight. Subclones were isolated by single colony isolation, and the DNA region that was generated by PCR was verified by Sanger sequencing at the DNA Laboratory core facility at Arizona State University. Complete sequences for the unique empty vectors pRSET-TEV-12His and pelB-MBP are in Supplementary Figs. S11 and S12, respectively. These vectors were designed to allow cloning into two *Bse*RI sites that flank a removable 0.4 kb fragment (“stuffer”). Importantly, because cleavage occurs outside of the *Bse*RI recognition site, cloning into these *Bse*RI-digested vectors avoids the need to include any of the restriction site recognition sequence in the expressed protein sequence. Complete sequences for representative subclones in each vector are shown in Supplementary Figs. S3 and S4.

The expression strains (Table 2) included C43(DE3), a BL21(DE3) mutant derivative that displays improved membrane protein expression^102^. Lemo21(DE3) is the same as BL21(DE3) but contains the pLemo plasmid that modulates expression from T7 promoters by rhamnose-inducible co-expression of T7 lysozyme (LysY)^103^. KTD101(DE3) is a (DE3) derivative of the trigger factor deficient (Δ*tig*) strain KTD101, which is from the *E. coli* K-12 lineage^104^. BL21-AI is a BL21 derivative that moderates expression from the T7 promoter due to a chromosomal copy of an arabinose-inducible gene encoding the T7 RNA polymerase^105^. To make the KTD101 strain^104^ compatible with expression from the T7 promoter, we used the λDE3 Lysogenization Kit (EMD Millipore #69734-3) to generate strain KTD101(DE3). Competent expression cells were generated and were transformed as described previously^106^ using 20–170 ng DNA and plating 10–100% of cells, or according to BL21-AI manufacturer instructions using 5–50 ng DNA and 15 µL cells. Petri plates with colonies were stored at 4 °C for up to 1 month.

### Expression growths

Unless specified, growths in liquid medium and on plates (1.5% agar) used LB Miller medium (10 g tryptone, 5 g yeast extract, and 10 g sodium chloride per liter) with the appropriate antibiotic (50 µg/mL carbenicillin, 30 µg/mL kanamycin, and/or 25 µg/mL chloramphenicol, according to Table 2 and Supplementary Table S3). The growth described in Fig. 4 used Terrific Broth (per liter: 12 g tryptone, 24 g yeast extract, 4 mL glycerol, 2.31 g KH_2_PO_4_, 12.54 g K_2_HPO_4_). In liquid medium, chloramphenicol was at 34 µg/mL. Large scale expression growths in BL21-AI were done as follows. An isolated colony from a transformation plate was used to inoculate 10 mL medium with 50 µg/mL carbenicillin. This culture was incubated overnight at 37 °C and with shaking at 250 rpm (230 rpm for Fig. 4). The next day, the overnight culture was added to 1 L of pre-warmed medium with 50 µg/mL carbenicillin in a 2 L flask. Incubation continued about 5 h until the culture OD_600_ was about 0.5 (in LB) or 1.3 (in Terrific Broth). Cultures were induced with arabinose (0.003% for Fig. 2; 0.2% for Fig. 4) and incubated 18 h at 25 °C. Cells were harvested by centrifugation at 8,000 × *g* for 20 min at 4 °C, and cell pellets were flash frozen in liquid nitrogen and stored at −80 °C.

### Isolation of a total cell membrane fraction and a screen for detergent extraction

A cell membrane fraction (containing both inner and outer membranes) was isolated and a detergent screen of this fraction (Fig. 2a) was performed as follows. A cell pellet from a 1 L expression growth culture was thawed and suspended in 10 mL (the volume is per gram wet weight of cells) of Protein Buffer (PBS [137 mM NaCl, 2.7 mM KCl, 10 mM Na_2_HPO_4_, 1.8 mM KH_2_PO_4_, pH 7.5] with one SIGMAFAST Protease Inhibitor Cocktail Tablet, EDTA-Free, for each 200 mL). Egg white lysozyme was added (final concentration 2 mg/mL) and the suspension was shaken at room temperature for 30 min. The lysate was sonicated on ice using a Branson Sonifier (10 s ON, 30 s OFF, total 1 min ON, at 60% power), and a cell lysate sample was collected for later analysis (Fig. 2a, lane 2). A low speed centrifugation step (8000 × *g* for 10 min at 4 °C) yielded supernatant (Fig. 2a, lane 3) and pellet (Fig. 2a, lane 4). The supernatant was centrifuged at high speed (100,000 × *g* for 1 h at 4 °C) to isolate the cell membranes. The supernatant (Fig. 2a, lane 5), containing soluble proteins (i.e., non-membrane-bound cytoplasmic and periplasmic proteins) was separated from the pellet (Fig. 2a, lane 6), containing the cell membrane fraction. The membrane pellet was suspended using a homogenizer (glass vessel tissue grinder with polytetrafluoroethylene pestle) in 32 mL Protein Buffer. A sample was collected from both fractions for later analysis. Eight 4 mL suspensions were aliquoted into 15 mL tubes, and detergent was added to each sample to a final concentration of 2% DM (*n*-decyl-β-D-maltoside), DDM (*n*-dodecyl β-D-maltoside), C_12_E_8_ (octaethylene glycol monododecyl ether), OG (*n*-octyl-β-D-glucopyranoside), CHAPS (3-[(3-cholamidopropyl)dimethylammonio]-1-propanesulfonate), LDAO (lauryldimethylamine-*N*-oxide), CYMAL-2 (2-cyclohexyl-1-ethyl-β-D-maltoside), or CYMAL-6 (6-cyclohexyl-1-hexyl-β-D-maltoside), and shaken overnight at 4 °C. The detergent/membrane solubilized suspensions were centrifuged at 100,000 × *g* at 4 °C for 1 h to yield a detergent-soluble supernatant and a detergent-insoluble pellet fraction. For gel loading, the detergent-insoluble pellet fraction was homogenized in 4 mL of 8 M urea. All other gel-loading samples were suspended in SDS-PAGE loading buffer (1X XT Sample Buffer [Bio-Rad #161-0791], 715 mM 2-mercaptoethanol). PelB-P66 was similarly treated except that the low-speed centrifugation steps was done at 20,000 × *g*, and the high-speed was at 50,000 × *g* for 2 h. All eight detergents screened are shown in Supplementary Fig. S9. The Supplementary Methods describes SDS-PAGE, Western blot analyses, silver stain, and the small-scale purification by nickel affinity in Fig. 2b.

### Isolation of a cell outer membrane fraction, detergent extraction, and purification of BBA57

A sarkosyl-insoluble cell OM fraction was isolated with N-lauroyl sarcosine, and BBA57 was solubilized with 2% DDM (Fig. 4a,b) as follows. Cell pellets from 1 L expression growth cultures were thawed and suspended in Protein Buffer (all pellets in prep were resuspended in 10 mL Protein Buffer per 1 g pellet mass) with 20 mM imidazole (without lysozyme). The lysate was sonicated on ice using a Branson Sonifier (5 s ON, 5 s OFF, total 1 min ON, at 60% power, repeated 3x). A low speed centrifugation step (5,000 × *g* for 10 min at 4 °C) yielded supernatant and pellet. The supernatant was centrifuged at high speed (150,000 × *g* for 1 h at 4 °C). The supernatant, containing soluble proteins (i.e., non-membrane-bound cytoplasmic and periplasmic proteins), was separated from the pellet, containing the cell membrane fraction. The membrane pellet was suspended using a homogenizer in Protein Buffer. *N*-Lauroyl sarcosine sodium salt (sarkosyl) was added to the suspension to a final concentration of 0.5% and incubated with shaking for 30 min at room temperature. The sarkosyl-insoluble OM pellet was collected by high speed centrifugation (150,000 x g for 1 h at 4 °C). The OM pellet was washed by homogenizing in Protein Buffer with 20 mM imidazole, and centrifuged at high speed (150,000 × *g* for 1 h at 4 °C). The washed OM pellet was resuspended in Protein Buffer with 2% DDM and 20 mM imidazole and incubated overnight with shaking at 4 °C. The solution was centrifuged at 150,000 × *g* at 4 °C for 1 h to yield a detergent-soluble supernatant and a detergent-insoluble pellet fraction. The detergent-soluble BBA57 was purified over a 5 mL GE Healthcare HisTrap HP column on an ÄKTA pure system at 4 °C. Insoluble particles were removed by centrifugation at 17,000 × *g* for 10 min prior to loading. The bound protein was washed with chilled 20 mM Tris pH 8.0, 500 mM NaCl, 0.05% DDM, 20 mM imidazole, and then washed with chilled buffer containing 80 mM imidazole. BBA57 was eluted with the same buffer containing 500 mM imidazole and collected in 1 mL fractions. LC/MS/MS using an Orbitrap Fusion Lumos Tribrid was performed by the Mass Spectrometry Facility at Arizona State University.

### Gel filtration chromatography

Nickel purified DDM-soluble BBA57 was purified by gel filtration chromatography from nickel-purified fractions that were pooled and concentrated down to 1 mL using a 30 K cutoff spin filter. The concentrated sample was purified over a GE Superdex 200 Increase 10/300 column on an ÄKTA pure system. Circular dichroism is described in Supplementary Methods.

### Negative stain imaging

BBA57 at 0.01 mg/mL was diluted 2-fold before applying on a glow-discharged homemade carbon coated grid. The grid was washed several times with water that was removed by side blotting, followed by a staining step with 0.75% uranyl formate solution. For imaging, the grid was inserted into a Phillips CM12 microscope at the Eyring Materials Center at Arizona State University. The image was obtained in 140 kX magnification.

## Supplementary Information

Supplementary Information

## Data Availability

No datasets were generated or analyzed during the current study.
