# Author Correction: Membrane directed expression in *Escherichia coli* of BBA57 and other virulence factors from the Lyme disease agent *Borrelia burgdorferi*

**DOI:** 10.1038/s41598-020-60882-x

**Published:** 2020-03-02

**Authors:** Karie E. Robertson, Chloe D. Truong, Felicia M. Craciunescu, Jay-How Yang, Po-Lin Chiu, Petra Fromme, Debra T. Hansen

**Affiliations:** 1 Biodesign Center for Applied Structural Discovery, Arizona State University, 1001 S. McAllister Avenue, Tempe, AZ 85287 USA; 2 School of Molecular Sciences, Arizona State University, 551 East University Drive, Tempe, AZ 85287 USA; 3 Biodesign Center for Innovations in Medicine, Arizona State University, 1001 S. McAllister Avenue, Tempe, AZ 85287 USA

Correction to: *Scientific Reports* 10.1038/s41598-019-53830-x, published online 26 November 2019

Jay-How Yang was omitted from the author list in the original version of this Article. This has been corrected in the PDF and HTML versions of the Article, and in the accompanying Supplementary Information file.

The Author Contributions section now reads:

K.E.R. performed most of the work; C.D.T. performed the negative stain with input from P.C.; J.Y.H. performed initial negative stain screening; F.M.C. developed the small scale screen and performed most of the cloning; K.E.R. and D.T.H. wrote the paper with input from P.F.; D.T.H. and P.F. directed the study.

