# Supplementary material for: Membrane directed expression in Escherichia coli of BBA57 and other virulence factors from the Lyme disease agent Borrelia burgdorferi
Source: Sci Rep. 2019 Nov 26;9:17606. doi: 10.1038/s41598-019-53830-x (PMC6879480; doi:10.1038/s41598-019-53830-x)
Supplement: Supplementary file 1 — Supplementary Information [file 41598_2019_53830_MOESM1_ESM.pdf]

Supplementary Information for

Membrane directed expression in *Escherichia coli* of BBA57 and other virulence factors from the Lyme disease agent *Borrelia burgdorferi*

Karie E. Robertson, Chloe D. Truong, Felicia M. Craciunescu, Po-Lin Chiu, Petra Fromme, Debra T. Hansen

Contents **page**

Supplementary Methods S2-S3

Supplementary Figures 1 – 12 S4-S23

Supplementary Tables 1 – 3 S24-S30

Supplementary References S31-S39

SUPPLEMENTARY METHODS

Sequence analyses. BLASTp was used to identify structures of homologous proteins in the Protein Data Bank[1]. Membrane association was analyzed using the structure prediction programs BOMP, HMMTOP, LipoP, MEMSAT, PRED TMBB, TBBpred, TMpred, TMHMM, BOCTOPUS and Jufo9d[2-11].

Small-scale screen of total cellular protein for detergent-solubilization and cobalt-affinity of His-tagged targets. Due to the multiple targets, strains, and expression conditions, a rapid screen was used to simultaneously identify if the expressed target was both (1) soluble in detergent and (2) able to bind via its His-tag to cobalt (TALON) magnetic beads (Fig. S5).

For the small-scale expression growths, a starter culture was prepared by inoculating a single colony from a transformation plate into 3 mL of medium plus antibiotic in a 14 mL snap-cap tube (BD #352057). This starter culture was incubated overnight at 37°C with shaking at 250 rpm. The next day, 5.5 mL of pre-warmed medium with antibiotic was inoculated with 275 µL of the overnight culture. Incubation continued until the OD_{600} reached approximately 0.5, at which point cells were induced with 0.5 mM IPTG or 0.2% arabinose. The cultures were incubated for 3 h. Cell pellets were collected by centrifugation in 1.5 mL tubes by centrifugation at 17,000 x *g* for 1 min. Culture supernatant was discarded. Cell pellets were collected from two culture volumes: 50 µL for a total protein sample, and 3 mL for the detergent/TALON screen. The cell pellets were frozen at -20°C until use.

For the small-scale detergent/TALON screen, cell pellets from the 3 mL of culture were thawed at room temperature for ~5 min. The remaining steps were done at room temperature unless noted. Thawed cell pellets were suspended by careful pipetting, to avoid bubble formation, in 200 µL of freshly prepared Lysis Buffer (50 mM sodium phosphate, pH 7.3, 300 mM NaCl, 20 mM imidazole, 1% *n*-dodecyl β-D-maltoside [DDM], 2 mg/mL chicken egg white lysozyme [Sigma #L6876-10G], 0.01 mg/mL deoxyribonuclease I from bovine pancreas [Sigma #DN25-100mg], 20 mM $MgCl_2$, plus one cOmplete Mini EDTA-free protease inhibitor tablet [Roche #11836170001] for every 10 mL buffer). Samples were incubated for 15 min, with vortexing every 5 min. Samples were placed in a pre-cooled rack at -80°C for at least 10 min, then thawed completely in a room-temperature tube rack (~5 min), and mixed once by vortex for 5 sec; these steps were repeated for a total of three times. Samples were incubated on ice for 30-60 min to allow detergent solubilization, then centrifuged at 17,000 x *g* for 15 min at 4°C. Most (~90%) of the supernatant, which includes the detergent-soluble fraction of total cell protein, was transferred to a separate tube and placed on ice, and the remaining supernatant that was nearest to the lysed cell pellet was discarded. The lysed cell pellet was stored at -20°C and later suspended in 100 µL of 8 M urea, 1X PBS (137 mM NaCl, 2.7 mM KCl, 10.1 mM Na_2HPO_4, 1.8 mM KH_2PO_4, pH 7.4) for SDS-PAGE analyses. For binding to TALON (cobalt) magnetic beads, a portion (120 µL) of the supernatant on ice was transferred to a fresh 1.5 mL tube on ice, and the remaining supernatant sample was stored at -20°C for later SDS-PAGE analyses. To the 120 µL supernatant sample was added 120 µL of ice-cold Buffer A (50 mM sodium phosphate, pH 7.3, 300 mM NaCl, 20 mM imidazole, 1% DDM) plus 40 µL of TALON magnetic beads (Clontech #635636) that had been washed four times in an equal volume of ice-cold Buffer A. Protein-bead samples were mixed by rocking at 4°C for 1 h to allow binding to cobalt. The tube was placed on a magnet, and the supernatant removed and stored at -20°C for later analysis of lack of binding to cobalt. The sample beads were then

washed four times, each time by adding 1 mL of ice-cold Buffer A, inverting the tube four times, placing the tube on the magnet, and then removing and discarding the supernatant. The last two washes each included 5 min of gentle rocking at 4°C after adding Buffer A and before collecting the beads using the magnet. Bound protein was eluted from the beads with 30 µL of SDS-PAGE loading buffer (1X XT Sample Buffer [Bio-Rad #161-0791], 715 mM 2-mercaptoethanol). Each 5 µL of this sample represents approximately 300 µL of the original culture. These samples were denatured at 95°C for 5 min just prior to loading SDS-PAGE.

Small-scale purification by nickel affinity. Nickel silica spin columns and nickel sepharose magnetic beads were used to verify metal binding (Fig. 2b) of BBA57-His$_{12}$ that was prepared from detergent soluble fractions from a 1 L culture. All steps and solutions were performed at room temperature unless noted. The detergent soluble fraction (600 µL) was loaded onto an Ni-NTA Spin Column (Qiagen #31014) that was equilibrated in Wash Buffer (50 mM sodium phosphate, 300 mM NaCl, 5 mM imidazole). The flowthrough was collected by centrifuging the tube at 270 x *g* for 10 min at room temperature. The spin column was washed twice with 600 µL Wash Buffer with centrifugation at 890 x *g* for 2 min. Protein was eluted twice with 300 µL of Elution Buffer (Wash Buffer with 500 mM imidazole) using centrifugation at 890 x *g* for 2 min. For the magnetic beads, HisMag nickel Sepharose Magnetic Beads (GE Healthcare #28-9781-19-AB) were prepared by adding 200 µL of beads to a 1.5 mL tube, removing the storage solution, and resuspending in 500 µL Wash Buffer. Detergent soluble sample (1 mL) was added to 500 µL prepared beads in a 1.5 mL tube and mixed by rocking on a shaker at 4°C for 1 h to allow binding. Using a magnet to remove the beads, the beads were washed twice with 500 µL Wash Buffer. Bound protein was removed with 100 µL Elution Buffer, which included 1 min incubation, and elution was repeated once.

SDS-PAGE, Western blot analysis, and silver stain. SDS-PAGE and Westerns were performed as described previously[12], with the following changes. MES(4-morpholineethanesulfonic acid)-SDS running buffer was used for SDS-PAGE. Samples were transferred to the nitrocellulose membrane at 350 mA for 1 h at 4°C. The membrane was blocked in TBST (20 mM Tris, 136 mM NaCl, pH 7.4, 0.05% Tween-20) with 5% BSA at room temperature for 30 min. Prior to gel loading, samples were heated in denaturing loading buffer at 95°C for 5 min, unless noted. Anti-His antibody was Qiagen Penta-His mouse monoclonal IgG1 (#34660; RRID:AB_2619735). Secondary antibody was HRP-conjugated anti-mouse-IgG (H+L) from goat (Invitrogen #626520) or sheep (Jackson ImmunoResearch Labs # 515-035-062; RRID:AB_2340296). Western molecular weight standards were Life Technologies MagicMark XP Western Protein Standard (#LC5603), which is recognized by anti-IgG secondary antibody. Silver stained gels were stained with the Pierce Silver Stain Kit (#24612) and used Bio-Rad Precision Plus Protein Dual Color Standards (#161-0374). Positive control proteins were CapA-TEV-GFP-His$_6$ (CapA is a protein from *Francisella tularensis*) and GFP-His$_6$[13].

Circular dichroism. Circular dichroism was performed on a Jasco J-815 Circular Dichroism Spectropolarimeter using purified 0.1 mg/mL sample in a 0.1 cm cuvette. Data was collected in continuous scanning mode at a 100 nm/min scanning speed, 1 nm scanning increment, 1 s response rate, and 1 nm bandwidth. The spectrum (Fig. 5c) represents the background subtracted average of three scans with a smoothing window of 8. Secondary structure features were determined using DICHROWEB[14-16] and analyzed with CDSSTR[17-19], CONTIN-LL[20,21], and SELCON3[22,23] using three different reference sets: SMP180[24], Set 4, and Set 7[17,25]. The outputs from the three analysis programs and reference sets were averaged for improved reliability of secondary structure predictions.

a

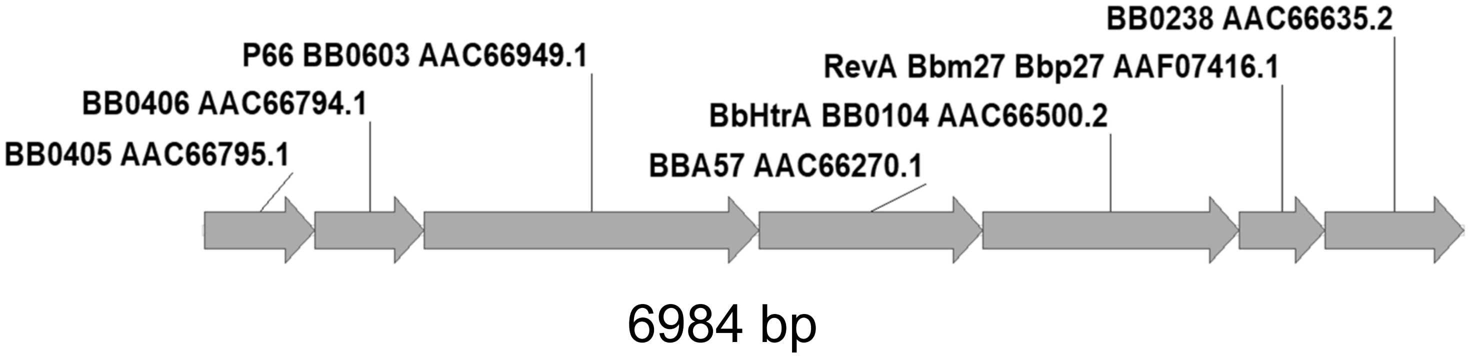

b

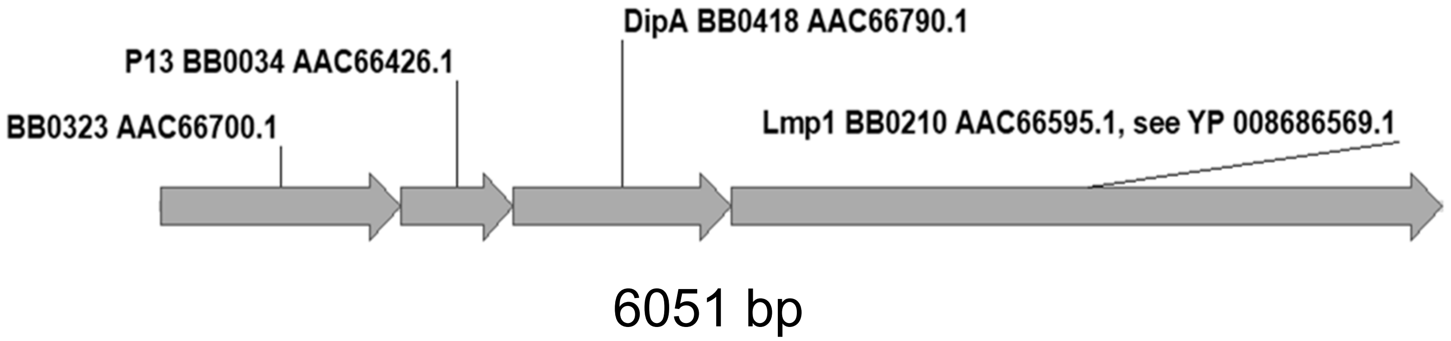

Figure S1. Schematic of the DNA inserts containing tandem copies of the expression-optimized genes that were used as template DNA in PCRs for the production of expression clones for the 11 targets in Table S1. (**a**) Insert from pUC57-BBclone1 containing 7 targets. (**b**) Insert from pCC1-4k-BBclone2 containing 4 targets.

a

```
LOCUS       pUC57-BBclone1\Genscript\INSERT\DTH        6984 bp    DNA     linear       15-FEB-2019
SOURCE
  ORGANISM
COMMENT
FEATURES             Location/Qualifiers
     CDS             1..609
                     /vntifkey="4"
                     /label=BB0405\AAC66795.1
     CDS             610..1218
                     /vntifkey="4"
                     /label=BB0406\AAC66794.1
     CDS             1219..3072
                     /vntifkey="4"
                     /label=P66\BB0603\AAC66949.1
     CDS             3073..4314
                     /vntifkey="4"
                     /label=BBA57\AAC66270.1
     CDS             4315..5736
                     /vntifkey="4"
                     /label=BbHtrA\BB0104\AAC66500.2
     CDS             5737..6216
                     /vntifkey="4"
                     /label=RevA\Bbm27\Bbp27\AAF07416.1
     CDS             6217..6984
                     /vntifkey="4"
                     /label=BB0238\AAC66635.2
BASE COUNT     2063 a      1649 c      1908 g      1364 t
ORIGIN
        1 atgcgtatgc tgctggcgac catcattctg attctgacca ccggtctgct ggcggcgcag
       61 agcaaaagca agagcatgac cgaggacgat ttcgactttg ataaactgct ggcgaaagag
      121 gagagcgtgc gtcgtctgtt tggcatcggt ttcggcgttg gttacccgct ggcgaacatc
      181 accattagcg tgccgtatgt tgacattgat ctgggttacg gtggctttgt gggcctgaag
      241 ccgaacaact tcctgccgta tgtggttatg ggtgttgatc tgctgttcaa agacgaaatt
      301 cacaagaaca ccatgatcag cggtggcatc ggcattggtg cggactggag caaaggcagc
      361 ccggagaaaa gcaacgaaaa gctggaggaa gaggaagaga acgaggcgca gcaagtggcg
      421 agcctgcaga accgtattgg tgtggttatc cgtctgccgc tggttattga atacagcttt
      481 ctgaaaaaca tcgtgattgg cttcaaggcg gttgcgacca tcggcaccac catgctgctg
      541 ggcagcccga tgagctttga gggtgcgcgt ttcaactttc tgggcaccgg tttcatcaag
      601 atctacatca tgatcaagat cttcaagaaa atttacatcc tgaccctggt gctgggcatg
      661 gcgcacctga gctttgcgag cgataactac atggttcgtt gcagcaagga agaggacagc
      721 accacctgca ttgcgaaact gaaggagatc aaagaaaaga aaaactatga cctgttcagc
      781 atgggtattg gtatcggcga cccgatcgcg aacattatga tcaccattcc gtatatcaac
      841 attgattttg gttacggtgg cttcattggc ctgaagagca acaactttga aaactatctg
      901 aacggtggca tcgacgttat cttcaagaag cagatcggtc aatacatgaa gatcggtggc
      961 ggtatcggta ttggcgcgga ttggagcaaa accagcctga tcccgccgaa cgaagaggaa
     1021 gagaccgatt acgagcgtat tggcgcggtg atccgtattc cgtttatcat ggaatataac
     1081 ttcgcgaaaa acctgagcat tggtttcaag atctatccgg cggttggtcc gaccatcctg
     1141 ctgaccaaac cgagcattct gtttgagggt atcaagttca acttctttgg tttcggcttt
     1201 attaaattcg cgtttaacat gaagagccac atcctgtaca aactgatcat ttttctgacc
     1261 accagcgcgg cgatctttgc ggcggatgcg ctgaaagaaa aggacatctt caaaattaac
     1321 ccgtggatgc cgaccttcgg ttttgagaac accagcgaat ttcgtctgga catggatgag
     1381 ctggtgccgg gcttcgaaaa caaaagcaag atcaccatta aactgaagcc gtttgaggcg
     1441 aacccggaac tgggcaagga cgatccgttc agcgcgtaca ttaaagtgga ggatctggcg
     1501 ctgaaggcgg aaggtaagaa aggcgaccaa ttcaaaatcg atgttggcga cattaccgcg
     1561 cagatcaaca tgtacgactt ctttatcaag attagcacca tgaccgactt cgattttaac
     1621 aaagagagcc tgttcagctt tgcgccgatg accggtttta agagcaccta ctatggcttc
     1681 ccgagcaacg atcgtgcggt tcgtggcacc atcctggcgc gtggcaccag caaaaacatc
     1741 ggcaccattc aactgggcta caaactgccg aagctggacc tgacctttgc gatcggtggc
     1801 accggcaccg gtaaccgtaa ccaggaaaac gacaaggata ccccgtacaa caaaacctat
     1861 cagggcattc tgtatggtat ccaagcgacc tggaaaccga ttaagaacct gctggatcag
     1921 aacgaggaca ccaagagcgt gatcgcggag accccgtttg aactgaactt cggtctgagc
     1981 ggcgcgtatg gtaacgaaac ctttaacaac agcagcatca cctacagcct gaaagataag
     2041 agcgtggttg gtaacgacct gctgagcccg accctgagca acagcgcgat tctggcgagc
     2101 ttcggtgcga aatataagct gggcctgacc aaaattaacg ataagaacac ctacctgatc
     2161 ctgcagatgg gcaccgattt tggtatcgac ccgttcgcga gcgactttag cattttcggt
     2221 cacatcagca aggcggcgaa cttcaagaaa gagaccccga gcgatccgaa caagaaagcg
     2281 gaaatctttg acccgaacgg taacgcgctg aatttcagca aaaacaccga gctgggcatt
     2341 gcgtttagca ccggcgcgag catcggtttc gcgtggaaca aagataccgg cgagaaggaa
     2401 agctgggcga tcaagggcag cgatagctac agcacccgtc tgtttggtga acaagacaag
     2461 aaaagcggcg tggcgctggg tattagctat ggccagaacc tgtaccgtag caaagacacc
     2521 gagaaacgtc tgaagaccat tagcgaaaac gcgttccaaa gcctgaacgt tgagatcagc
     2581 agctatgaag ataacaagaa aggtatcatc aacggtctgg gctggatcac cagcattggc
     2641 ctgtatgaca ttctgcgtca gaaaagcgtg gagaactacc cgaccaccat cagcagcacc
     2701 accgagaaca accagaccga acaaagcagc accagcacca agaccaccac cccgaacctg
     2761 accttcgagg atgcgatgaa actgggtctg gcgctgtacc tggactatgc gatcccgatt
     2821 gcgagcatca gcaccgaagc gtacgtggtt ccgtatattg gtgcgtacat cctgggcccg
     2881 agcaacaagc tgagcagcga tgcgaccaaa atctacctga agaccggtct gagcctggag
     2941 aaactgattc gttttaccac catcagcctg ggctgggaca gcaacaacat cattgaactg
     3001 gcgaacaaga acaccaacaa cgcggcgatt ggtagcgcgt ttctgcaatt caaaatcgcg
     3061 tacagcggta gcatgaacgg caaactgcgt aaggcgctga aaatcgcgat tttcaccacc
     3121 ctgctgctgg tgatcagctg caacgcgaac atggatacca acgacaaaaa caaggcgctg
     3181 aacgagtata aactgaagaa cattagcgaa gttatcaaga acagcctgca gctggagagc
     3241 gacccgaaac tgaagaaaga gccggaaagc aacatcaacc aaagcacccc gccgatcctg
     3301 gagattgaaa aaatcgagcc gggtaaacag gaaatgagcc tgaagagcga gttcggcagc
     3361 gaaagcctga tgccgctgga agagccggaa gaggcgaaca tggcgaaaag cgaagaggaa
     3421 attgcgaaaa tccaggagaa gctgctgctg attggtgcga gcgatgaaat caccgaccag
     3481 gagctgggcg aaaacatgca aaagtttctg aacccgacca ccgttgagtt caaaatcagc
```

(continued)

Figure S2. Complete DNA sequences for the expression-optimized sequences encoding the 11 *Bb* membrane protein targets. The genes are arranged in tandem in two vectors that were used as PCR template for expression subclones. (**a**) pUC57-BBclone1 (ampicillin^{R}) encodes 7 targets: BB0405, BB0406, P66, BBA57, HtrA, RevA and BB0238. (**b**) pCC1-4k-BBclone2 (chloramphenicol^{R}) encodes 4 targets: BB0323, P13, DipA and Lmp1.

```
3541 accaccacca acgaaaccat tctgaccacc atcgaggaag aggaaatcaa caacaacaag
3601 gataagatct tcgaccacaa agaggagaac gtgaccctgg gtaacaacag cctggagaac
3661 gcgaccctga acaagaacac cattaccctg gcgcagaacc aaaaatatac cacccacctg
3721 aaaaacgacg ataagtttat caccaaagaa tacctgaagc aagttcgtga tagcctggac
3781 aaggcgctga acgcgattaa aaacctggag accagcaagg agttccgtga gctggaaaac
3841 ctgccgaaag atcaggagaa cagcccgagc gcgaagaaag agagcaccga aagcaacatt
3901 aacaacacca acaccgagaa catcgaaatc attaaaaacc gtctgctgga ggaactgaac
3961 aagagcgagc tgatcttcga agataccaaa gacccgctgg gcaccagcac cattaagaaa
4021 gtgatcgacg cggcgaagaa atggcaggag aaggaaaaca gcagccaaat cgactgggat
4081 ctgggcttca aatttcaccc gaacccgaaa ttttataaca agctgaccgc gcaagagtac
4141 aaagtgctgg cggaaaagtt caccaaagtt aagaacgagt acaagaacac caaagagcag
4201 ctgaaagcgg aaagcaagct gaccaccaac agcatcagca aaatcattaa cgcgaccaag
4261 gagttcgcga accaagtgat taacctgatc ctgctggttg agaagaacta ccagatggaa
4321 aagaaattct ttagcggttt tctgctgagc ttcctggcgc tgagcattgg tttctttatc
4381 ggcatgcact atctggcgag caaccgtagc aacatcgtgt ttgcggagga aaaagataac
4441 accgttcgtg cgctgcaaga cagcttccgt gaggtgagca agaaaattct gccgagcagc
4501 gttgaagtgc acgcgaccgg tgttattaag cagagctttc cgatcccgtt ctttttcttt
4561 gatatgccgg agttcgacag cgaacgtaaa agcaactggg cgggtagcgg cgttatcatt
4621 ggccgtgaca gccagaagaa aagcctgttt tacgtggtta ccaacagcca cgtggttgat
4681 aaggcgaccg agctggaagt ggttagctat gacaagaaaa agcacaaagc gaagctgatc
4741 ggtaaagatg agaaaaagga cattgcgctg atcagcttcg aaagcgacga tgcgaccatt
4801 aaggtggcgg atctgggcga cagcgataaa ctggagatcg gtgactgggt gatggcggtt
4861 ggcagcccgt tccaatttag cttcaccgtg accgcgggta tcgttagcgg tctgcagcgt
4921 agcgcgaacc cgaacctgca gagccgtaac ctgttcattc aaaccgatgc ggcgatcaac
4981 cgtggtaaca gcggcggtcc gctggtgaac atcaagggtg aggttatcgg cattaacgcg
5041 tggattgcga gcaacagcgg cggtaacatc ggtctgggct ttgcgattcc ggtgaacaac
5101 atcaaaagca ccgttgactt ctttctgaag ggtaaaaaga ttgaaagcgc gtggctgggc
5161 atcagcttct acccgctgaa aacccgtgat agcgaggtgc tgaagagcct gggtgtggaa
5221 agcaacgacg ttagcgcggc gatcattgcg agcctgtatc cgggcagccc ggcggttaag
5281 agcggtctgc gtgcgggcga catcattatg aaagtgaacg gtgttagcat gagcgtgttt
5341 caagatgtta ccagctatat tagcgacttc tacgcgggcg agaaggttaa cgtggaaatc
5401 ctgcgtggca acgtgaaaaa gaacatcgag attgtgctgg cggttcgtcc gaaagataag
5461 gagctgagca gcagcaagat gctgccgggt tttgtggttt acccgctggt tgaggacatc
5521 aaagcgcagc tgaacctgcg taactggatt aagggtgtgg ttgtggatta catcgacaaa
5581 aacctggcga gcaacattaa aatgaagagc ggcgacgtga tcctgagcgt taacagcaaa
5641 agcgtgagca acctgcgtga gttctatgat gcgctggaag ttggtaaaaa cacctacaag
5701 atcctgcgtg gcaacgacag ctttaagatt accttcatgc gtaacaaaaa catctttaag
5761 ctgttctttg cggcgatgct gttcgtgatg gcgtgcaaag cgtacgttga ggagaagaag
5821 gagatcgata gcctgatgga agacgtgctg gcgctggtta acgacagcag cggcggtaaa
5881 ttcaaggatt acaaggacaa gatcaacgag ctgaaagaaa acctgaagga catcggtaac
5941 gcggagctga aagaaaagct gctgaacctg cagaacagct tccaagacaa actggcggcg
6001 aagctggcgg cgctgaaggc ggcgaaaaac accattgaga acatcaccga caaggatcaa
6061 gacattagca agcgtaagat ctggagcgaa gcgaaactgg ttggtgtgac cgttccgctg
6121 ctgggcagca acaccagcgg taacggcgat aagatgagca aaaacgcggt ggagcagatt
6181 gacaaggtta tcaaattcct ggaggaaggc accaacatgc gtcgtctgtt cctgctgtat
6241 atcctgtgca gctttgtttt cctgaacctg tttgcgcaag gtagcagcag ctacattgac
6301 aaacagaagg agctggcgat cttctactat gaagtgggcc aacgttacat caacgttggc
6361 aagatcaaaa agggcaagct gtttcaggcg aaggcgctga aaatttaccc ggatctgaaa
6421 aagggtttcg acatcaaact ggcggtgaag gagctggatg cgcgtattaa agacgataac
6481 ccgaaggttg tgatgctgga agacatcaag ctggaggaaa ttccgggcat cgttcacgag
6541 aagatcgaaa tcaacgattt caccaacgcg ccgaagattg agtacatcgc gcagcgtgaa
6601 cgtagcaaga accaagacaa gatcatcaag ttccagttcg gcaagttcgc gcgtgcgctg
6661 attagccgta actttgacct gttcgacagc gtgatcgcgg ataaggtgaa cgttatgggc
6721 caatttgaaa gcaaaaacga cttcatcagc accctgagca gcgcgagcag caaagcggat
6781 gcggacgagc tggaatacct gagcgtggac gattactatg atctgaagag cctgaaaatc
6841 agcaagagca acgacaccag cttcgcggtg aacgttaacg cgaaaaagaa cgacgttacc
6901 aaaaactttc cgttctggaa ggagcgtcag accctgattt ttaccaccga agacgataac
6961 aactggttcc tgagcagcat caac
//
```

(continued)

Figure S2 (continued).

b

```
LOCUS       pCC1-4k-BBclone2\Genscript\INSERT\v2\DTH        6051 bp    DNA     linear       15-FEB-2019
SOURCE
  ORGANISM
COMMENT
FEATURES             Location/Qualifiers
     CDS             1..1131
                     /vntifkey="4"
                     /label=BB0323\AAC66700.1
     CDS             1132..1668
                     /vntifkey="4"
                     /label=P13\BB0034\AAC66426.1
     CDS             1669..2694
                     /vntifkey="4"
                     /label=DipA\BB0418\AAC66790.1
     CDS             2695..6051
                     /vntifkey="4"
                     /label=Lmp1\BB0210\AAC66595.1,\see\YP_008686569.1
BASE COUNT     2023 a      1441 c      1512 g      1075 t
ORIGIN
        1 atgaacatca aaaacaagct gatcagcctg ctgattgtgg ttgcgatcag cttcattgcg
       61 tgcaagaccc cgccggagag ccgtgaaagc aaaaacgcga agatcgcgca gccggacaac
      121 aaaaactttc aactgcgtga catcaaagat attaagaacg aactgattcg tgagcgtggc
      181 cacctgttct atagcaaaga atttaacgag gcggaacgtc tggaggaagc gatgaagcag
      241 agcttcagca agaaaaaggc gatcgagggt aacgaaattg cgctgaaagt gctggagcgt
      301 tacaagacca tcattcgtga gacccgtgag aagaaggaga agaccaacta cctgaaagaa
      361 aacatcgaga agtatctgaa cgacgcggaa gcgaacgagg cgtacatctg gattccgctg
      421 gagattgatg aggttaacaa cctgtatttc gaggcgaccc gtaaatacaa gaactatgac
      481 ctggataacg cgctggacat gtacagcaaa gcgtttaacc gtgcgcagca agcggcgaaa
      541 aacgcgaagg aagcgaaagc gctgaaggaa accgatgagc gtatgtacaa acagctgaag
      601 gcgctggagg cggcgagcaa cctgccgatc tatagcaaca acaaactgat taagccgagc
      661 ccgtggaacg gccgtgcgtt catcaaagaa cgtaacagcc acctgaacct gctgaacacc
      721 aacaaggaca cctacctgct gggcgaggcg gaaattagca tcccgattgt gctggcgtat
      781 gaggaaaaag ttgaaatcgc gaaaaacagc aagccgcaag agcaatttaa gaccctggaa
      841 ctgattgagc gtagccgtac cctgtgggaa aaaggtgtgg aggcgaaaaa cgttaagaac
      901 ttccgtctgg cgaacgaact gtttctggag agcgcgcgtt acctggaagc gtatcagagc
      961 aacgcgagca gcgagctgta cgttatcaag attggcaaca ccctgtgggg tatcagcaaa
     1021 aagctgtaca acgacccgta tctgtggccg aagatttggt tcgcgaaccg tcagaaaatc
     1081 caaaacccgg atctgattca cagcaactgg aagatcatta tcccggcgaa aatgaacaag
     1141 ctgctgatct tcgtgctggc gaccttttgc gttttcagca gctttgcgca ggcgaacgac
     1201 agcaaaaacg gcgcgtttgg tatgagcgcg ggtgaaaagc tgctggtgta cgagaccagc
     1261 aaacaggacc cgatcgttcc gttcctgctg aacctgttcc tgggttttgg cattggtagc
     1321 tttgcgcaag gcgacatcct gggtggcagc ctgattctgg gtttcgatgc ggtgggcatc
     1381 ggtctgattc tggcgggcgc gtacctggac atcaaggcgc tggatggtat taccaaaaag
     1441 gcggcgtttc agtggacctg gggcaaaggt gtgatgctgg cgggtgtggt tacgatggcg
     1501 gttacccgtc tgaccgagat tatcctgccg ttcacctttg cgaacagcta taaccgtaaa
     1561 ctgaagaaca gcctgaacgt ggcgctgggt ggcttcgaac cgagctttga cgttgcgatg
     1621 ggtcagagca gcgcgctggg cttcgagctg agctttaaaa agagctacat gctgatcaaa
     1681 aagatttgct tctgcctgct gctgctgcag gcgaagatta tcctgagcca agatcagcaa
     1741 aacctggaat ttaacaacga gtacttctac ttcaacttcc aaagcgcgta ctatccgccg
     1801 cacgaactgg gcaccaacgg cagcggtttc agccagagct ttaaacaccc gaacttccaa
     1861 aacatcgacc cgagcctgaa aattccggtg aactactggg gtggcatcaa gctgattagc
     1921 tacctgggtt actacaagaa cttcaaggcg ctgaacaacc cgaacagcat cttctttaag
     1981 aacaacggca tcgacattga tctgaacatc ggtctgagcc cggtgattgt tagcttcaaa
     2041 agcaccgtta gcttcacccc gatcgcgttt ctgaacctgt acgcgagcaa cgaactgggc
     2101 attggttggc aggcgtttgg cttcaaaggc gtgggtgttc acatcgagaa cggcaagtac
     2161 agcaacgcgg tggaatttta tagcgagatc accgcgggtg gccgttttca gttcgacctg
     2221 aacgcgattt tccaaggcga gtggacccac attatcaccg ttatcggtaa cgactttacc
     2281 tacctgatta acccgcacgc gaaaaaggat caactgtgga aatataaggc ggacgatggc
     2341 aagaacatca acggtatgct gattaaaccg tacgcgctgc tggcgtataa gatgccgatc
     2401 ccgctgaaca ccattggcct gctgtatgaa ggtaaaaccc agatcggcaa ggagcgtaac
     2461 attagcagct ggaaaaacaa gggctggggt agcgacatct tctaccacaa catcagcatt
     2521 gttgcgaact ttgagattat caaaccgctg accctgggcc tgcaattcaa gctgagcacc
     2581 aacccgacct ataccagcaa caccgcgggt ctggcggaca tcagcaagcg tattgcgacc
     2641 ggcaacagct acttttacta tgatagcatc ggtatgagca tcacctacaa gtacatgaac
     2701 aagaagcaca ccaacttcag cgtgctgctg ctgctgatct ttctgctgat tctgagcttc
     2761 ggtggctttg gttactatat ctaccagagc aaactgaacg acaagaaccg tgaaatcatg
     2821 ctgaacgagg tgaagaacag cgttattgat cgtaactaca aaaaggcgta tagcgttgcg
     2881 aaactgctgc aggacaagta cccgcaaaac gaggacatcg cgatgctgac caacaccctg
     2941 gcggaaattg cgaacagcag cccgttcgag agcaaagacc tgcagcgtga tagcgcgaac
     3001 caaatcctgg acaaaattaa gggccaggat aacaccaaga ccaacgtgaa cgaaaacttc
     3061 gacatcgcgt ttaacaaccg ttacatcaaa gacagcacca ttaccgagaa ctatagcgat
     3121 cgtaacgacg atgttggtat cgaggacgaa gatattagcg agttcaaaaa gagcaaaatc
     3181 ccggagaaaa ttaagccgaa caccaacccg aaggaagagg accagattat ccaaagcccg
     3241 aacccgaaac tgagcgtgaa cgatcagaag aacctgttta acctggagaa actgaaaaag
     3301 aacctgagcg gtaaaagcaa cagcgaaaac atcctgaacg acagccagaa aattgagaac
     3361 gataagcaaa acaccaacct gagcaaagaa aagaacagcg agaacatcct gaaaaccccg
     3421 gacaacagca agtacagcaa caacaacaac accaccagcc tgaaaaagat tagcagcaac
     3481 agccagaagg agagcgaact gagcccgccg agccaaacca ttatcggcaa aatctaccgt
     3541 ccgtacagct atctgattaa aaaggaactg tatgagatcc tggacgatat taacaccggt
     3601 cgtgtgaccc tgggcaaaaa ccgtctgaag gagctgatca aaaagggtct gagcaacaaa
     3661 ttccagaagg ttaacgaact gattgagaac agcaaaaaca aggaagcgag caacctgctg
     3721 ctgaccctga tcaaaaagga cattgagccg aacctgatca acattccgaa agatccgtac
     3781 aaaaaggaaa tcttccagct ggacaaagag gacaaaaagc cgcaatatct ggaagacctg
     3841 aaaagcaagg tgcacagcat caagccgatt gatctggaaa acaccaagag ccgtcagcaa
     3901 gcgatcaaag acctgaacga gtttctgaag aacaacccga acgatgcgca ggcgagcaaa
     3961 accctggcgc aggcgaacaa gattcaacac ctggaggacc tgaaaagcaa ggttcacagc
     4021 atcaaaccga tcgatctgga aaataccaaa agccgtcagc aggcgattaa ggacctgaac
     4081 gagttcctga aaaataatcc gaacgatgcg caagcgagca agaccctggc gcaagcgaat
     4141 aaaattcaac acctggaaga tctgaagagc aaagttcata gcattaaacc gattgatctg
     4201 gaaaatacca agagccgtca gcaggcgatc aaggatctga atgagtttct gaaaaataac
     4261 ccgaatgacg cgcaggcgag caagacttta gcgcaagcga ataagatcca gcacctggag
     4321 gatctgaaga gcaaggtgca tagcattaag ccgatcgatc tggaaaacac caaaagccgt
     4381 cagcaagcga ttaaagatct gaatgagttc ctgaagaata atccgaatga cgcgcaagcg
     4441 agcaaaactt tagcgcaggc gaacaaaatc caacacctgg aagacctgaa gagcaaagtt
     4501 cacagcatta aacctatcga tctggaaaac actaagagcc gtcagcaagc tattaaggat
     4561 ctgaacgagt ttctgaaaaa caatccgaat gatgcgcagg cgagcaagac attagcgcag
```

(continued)

Figure S2 (continued).

```
4621 gcgaacaaga tccaacacct ggaggatctg aaaagcaaag tgcacagcat caaacctata
4681 gatctggaaa atactaagag ccgtcagcag gctatcaagg atctgaacga gttcctgaag
4741 aataacccga atgatgcgca agcgagcaaa acattagctc aagctaataa gattcagcac
4801 ctggaagatc tgaaaagcaa ggttcatagc attaagccta tcgatctgga aaatactaaa
4861 agccgtcagc aagctataaa ggatttaaac gagttcctga aaaacaatcc gaacgacgct
4921 caggcgagca aaactctggc gcaagcgtac gaaaacaacg gcgacctgct gaaagcggaa
4981 aacgcgtatg agaaaattat caagctgacc aacacccagg aagaccacta caaactgggt
5041 attatccgtt tcaagctgaa aaagtatgaa cacagcatcg agagctttga ccaaaccatt
5101 aagctggacc cgaagcacaa aaaggcgctg cacaacaaag gtatcgcgct gatgatgctg
5161 aacaagaaca aaaaggcgat tgagagcttc gagaaagcga tccagattga caagaactac
5221 ggcaccgcgt actatcaaaa aggcatcgcg gaggaaaaga acggtgacat gcagcaagcg
5281 ttcgcgagct ttaaaaacgc gtacaacctg gataagaacc cgaactatgc gctgaaagcg
5341 ggcatcgtta gcaacaacct gggtaacttc aagcagagcg aggaatacct gaacttcttt
5401 aacgcgaacg cgaaaaagcc gaacgaaatc gcgatttata acctgagcat cgcgaaattt
5461 gaaaacaaca agctggagga aagcctggag accatcaaca aagcgattga cctgaacccg
5521 gaaaagagcg agtacctgta tctgaaagcg agcatcaacc tgaaaaagga gaactaccag
5581 aacgcgatca gcctgtatag cctggtgatt gaaaagaacc cggagaacac cagcgcgtac
5641 atcaacctgg cgaaagcgta tgaaaagagc ggtaacaaaa gccaggcgat tagcaccctg
5701 gagaaaatta tcaacaaaaa caacaagctg gcgctgaaca acctgggtat cctgtacaaa
5761 aaggagaaga actatcaaaa agcgatcgaa attttcgaga aagcgattat caacagcgac
5821 attgaagcga agtacaacct ggcgaccacc ctgatcgaga ttaacgacaa cacccgtgcg
5881 aaggatctgc tgcgtgaata caccaaactg aaaccgaaca acccggaggc gctgcatgcg
5941 ctgggcatta tcgaatataa cgagaataac aacgatcaga ccctgcgtga actgatcaaa
6001 aagttcccga actacaaaaa gaacgagaac atcaaaaaga ttatcggtat t
//
```

Figure S2 (continued).

a

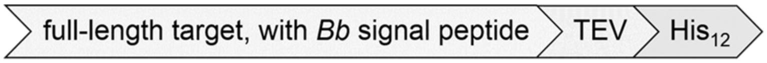


```
LOCUS       pBBA57-TEV-His12\KER        4091 bp    DNA     circular     18-MAY-2017
DEFINITION  T7 driven prokaryotic expression vector.
SOURCE
  ORGANISM
COMMENT
FEATURES             Location/Qualifiers
     promoter        20..39
                     /vntifkey="30"
                     /label=T7\promoter
     rep_origin      1650..2105
                     /vntifkey="33"
                     /label=f1\ori
     CDS             2236..3096
                     /vntifkey="4"
                     /label=AMP-R
     promoter        2137..2235
                     /vntifkey="30"
                     /label=bla\pr
     rep_origin      3241..3914
                     /vntifkey="33"
                     /label=pUC\ori
     RBS             85..92
                     /vntifkey="32"
                     /label=RBS
     terminator      1450..1579
                     /vntifkey="43"
                     /label=T7\term
     CDS             1342..1398
                     /vntifkey="4"
                     /label=BB0405-TEV-His12
     misc_feature    1342..1362
                     /vntifkey="21"
                     /label=TEV\cleavage\ENLYFQ*G
     misc_feature    1363..1398
                     /vntifkey="21"
                     /label=His12
     misc_feature    1399..1404
                     /vntifkey="21"
                     /label=double\stop\codon
     CDS             100..1341
                     /vntifkey="4"
                     /label=BBA57\AAC66270.1
     primer          20..39
                     /vntifkey="27"
                     /label=Primer\T7\(DNA\Lab)
     primer          complement(1492..1510)
                     /vntifkey="27"
                     /label=Primer\T7\terminator\(DNA\Lab)
BASE COUNT     1176 a      1011 c      1004 g       900 t
ORIGIN
        1 gatctcgatc ccgcgaaatt aatacgactc actataggga gaccacaacg gtttccctct
       61 agaaataatt ttgtttaact ttaagaagga gatatacata tgaacggcaa actgcgtaag
      121 gcgctgaaaa tcgcgatttt caccaccctg ctgctggtga tcagctgcaa cgcgaacatg
      181 gataccaacg acaaaaacaa ggcgctgaac gagtataaac tgaagaacat tagcgaagtt
      241 atcaagaaca gcctgcagct ggagagcgac ccgaaactga agaaagagcc ggaaagcaac
      301 atcaaccaaa gcaccccgcc gatcctggag attgaaaaaa tcgagccggg taaacaggaa
      361 atgagcctga agagcgagtt cggcagcgaa agcctgatgc cgctggaaga gccggaagag
      421 gcgaacatgg cgaaaagcga agaggaaatt gcgaaaatcc aggagaagct gctgctgatt
      481 ggtgcgagcg atgaaatcac cgaccaggag ctgggcgaaa acatgcaaaa gtttctgaac
      541 ccgaccaccg ttgagttcaa aatcagcacc accaccaacg aaaccattct gaccaccatc
      601 gaggaagagg aaatcaacaa caacaaggat aagatcttcg accacaaaga ggagaacgtg
      661 accctgggta acaacagcct ggagaacgcg accctgaaca agaacaccat taccctggcg
      721 cagaaccaaa aatataccac ccacctgaaa aacgacgata agtttatcac caaagaatac
      781 ctgaagcaag ttcgtgatag cctggacaag gcgctgaacg cgattaaaaa cctggagacc
      841 agcaaggagt tccgtgagct ggaaaacctg ccgaaagatc aggagaacag cccgagcgcg
      901 aagaaagaga gcaccgaaag caacattaac aacaccaaca ccgagaacat cgaaatcatt
      961 aaaaaccgtc tgctggagga actgaacaag agcgagctga tcttcgaaga taccaaagac
     1021 ccgctgggca ccagcaccat taagaaagtg atcgacgcgg cgaagaaatg gcaggagaag
     1081 gaaaacagca gccaaatcga ctgggatctg ggcttcaaat ttcacccgaa cccgaaattt
     1141 tataacaagc tgaccgcgca agagtacaaa gtgctggcgg aaaagttcac caaagttaag
     1201 aacgagtaca agaacaccaa agagcagctg aaagcggaaa gcaagctgac caccaacagc
     1261 atcagcaaaa tcattaacgc gaccaaggag ttcgcgaacc aagtgattaa cctgatcctg
     1321 ctggttgaga agaactacca ggaaaatctt tattttcaag gtcaccatca tcaccaccat
     1381 caccatcatc accaccatta ataaaagggc gaattccagc acactggcgg ccgttactag
     1441 tggatccggc tgctaacaaa gcccgaaagg aagctgagtt ggctgctgcc accgctgagc
     1501 aataactagc ataacccctt ggggcctcta aacgggtctt gaggggtttt ttgctgaaag
```

Full-length BBA57

(continued)

Figure S3. Complete and annotated (a) DNA and (b) protein sequences for pBBA57-TEV-His12, the His12-tagged, expression-optimized BBA57. The other targets' clones have the same sequence aside from the sequence representing the full-length target, which is shown here in blue.

```
     1561 gaggaactat atccggatct ggcgtaatag cgaagaggcc cgcaccgatc gcccttccca
     1621 acagttgcgc agcctgaatg gcgaatggga cgcgccctgt agcggcgcat taagcgcggc
     1681 gggtgtggtg gttacgcgca gcgtgaccgc tacacttgcc agcgccctag cgcccgctcc
     1741 tttcgctttc ttcccttcct ttctcgccac gttcgccggc tttccccgtc aagctctaaa
     1801 tcgggggctc cctttagggt tccgatttag tgctttacgg cacctcgacc ccaaaaaact
     1861 tgattagggt gatggttcac gtagtgggcc atcgccctga tagacggttt ttcgcccttt
     1921 gacgttggag tccacgttct ttaatagtgg actcttgttc caaactggaa caacactcaa
     1981 ccctatctcg gtctattctt ttgatttata agggattttg ccgatttcgg cctattggtt
     2041 aaaaaatgag ctgatttaac aaaaatttaa cgcgaatttt aacaaaatat taacgcttac
     2101 aatttaggtg gcacttttcg gggaaatgtg cgcggaaccc ctatttgttt atttttctaa
     2161 atacattcaa atatgtatcc gctcatgaga caataaccct gataaatgct tcaataatat
     2221 tgaaaaagga agagtatgag tattcaacat ttccgtgtcg cccttattcc cttttttgcg
     2281 gcattttgcc ttcctgtttt tgctcaccca gaaacgctgg tgaaagtaaa agatgctgaa
     2341 gatcagttgg gtgcacgagt gggttacatc gaactggatc tcaacagcgg taagatcctt
     2401 gagagttttc gccccgaaga acgttttcca atgatgagca cttttaaagt tctgctatgt
     2461 ggcgcggtat tatcccgtat tgacgccggg caagagcaac tcggtcgccg catacactat
     2521 tctcagaatg acttggttga gtactcacca gtcacagaaa agcatcttac ggatggcatg
     2581 acagtaagag aattatgcag tgctgccata accatgagtg ataacactgc ggccaactta
     2641 cttctgacaa cgatcggagg accgaaggag ctaaccgctt ttttgcacaa catgggggat
     2701 catgtaactc gccttgatcg ttgggaaccg gagctgaatg aagccatacc aaacgacgag
     2761 cgtgacacca cgatgcctgt agcaatggca acaacgttgc gcaaactatt aactggcgaa
     2821 ctacttactc tagcttcccg gcaacaatta atagactgga tggaggcgga taaagttgca
     2881 ggaccacttc tgcgctcggc ccttccggct ggctggttta ttgctgataa atctggagcc
     2941 ggtgagcgtg ggtctcgcgg tatcattgca gcactggggc cagatggtaa gccctcccgt
     3001 atcgtagtta tctacacgac ggggagtcag gcaactatgg atgaacgaaa tagacagatc
     3061 gctgagatag gtgcctcact gattaagcat tggtaactgt cagaccaagt ttactcatat
     3121 atactttaga ttgatttaaa acttcatttt taatttaaaa ggatctaggt gaagatcctt
     3181 tttgataatc tcatgaccaa aatcccttaa cgtgagtttt cgttccactg agcgtcagac
     3241 cccgtagaaa agatcaaagg atcttcttga gatccttttt ttctgcgcgt aatctgctgc
     3301 ttgcaaacaa aaaaaccacc gctaccagcg gtggtttgtt tgccggatca agagctacca
     3361 actctttttc cgaaggtaac tggcttcagc agagcgcaga taccaaatac tgttcttcta
     3421 gtgtagccgt agttaggcca ccacttcaag aactctgtag caccgcctac atacctcgct
     3481 ctgctaatcc tgttaccagt ggctgctgcc agtggcgata agtcgtgtct taccgggttg
     3541 gactcaagac gatagttacc ggataaggcg cagcggtcgg gctgaacggg gggttcgtgc
     3601 acacagccca gcttggagcg aacgacctac accgaactga gatacctaca gcgtgagcta
     3661 tgagaaagcg ccacgcttcc cgaagggaga aaggcggaca ggtatccggt aagcggcagg
     3721 gtcggaacag gagagcgcac gagggagctt ccagggggaa acgcctggta tctttatagt
     3781 cctgtcgggt ttcgccacct ctgacttgag cgtcgatttt tgtgatgctc gtcagggggg
     3841 cggagcctat ggaaaaacgc cagcaacgcg gcctttttac ggttcctggc cttttgctgg
     3901 ccttttgctc acatgttctt tcctgcgtta tcccctgatt ctgtggataa ccgtattacc
     3961 gcctttgagt gagctgatac cgctcgccgc agccgaacga ccgagcgcag cgagtcagtg
     4021 agcgaggaag cggaagagcg cccaatacgc aaaccgcctc tccccgcgcg ttggccgatt
     4081 cattaatgca g
//
```

b

```
LOCUS       Translation\of\pBBA57-TEV-His12\KER         433 aa                         29-JUN-2017
DEFINITION  Translation of a fragment of pBBA57-TEV-His12 KER.
KEYWORDS    TRANSLATED.
SOURCE
  ORGANISM
COMMENT
FEATURES             Location/Qualifiers
     Site            422..433
                     /vntifkey="264"
                     /label=His12
     Site            415..421
                     /vntifkey="264"
                     /label=TEV\cleavage\ENLYFQ*G
     Region          1..414
                     /vntifkey="1000"
                     /label=BBA57\AAC66270.1
ORIGIN
        1 mngklrkalk iaifttlllv iscnanmdtn dknkalneyk lknisevikn slqlesdpkl
       61 kkepesninq stppileiek iepgkqemsl ksefgseslm pleepeeanm akseeeiaki
      121 qekllligas deitdqelge nmqkflnptt vefkistttn etilttieee einnnkdkif
      181 dhkeenvtlg nnslenatln kntitlaqnq kytthlkndd kfitkeylkq vrdsldkaln
      241 aiknletske frelenlpkd qenspsakke stesninntn tenieiiknr lleelnksel
      301 ifedtkdplg tstikkvida akkwqekens sqidwdlgfk fhpnpkfynk ltaqeykvla
      361 ekftkvkney kntkeqlkae sklttnsisk iinatkefan qvinlillve knyqenlyfq
      421 ghhhhhhhhh hhh
//
```

Full-length BBA57

Figure S3 (continued).

a

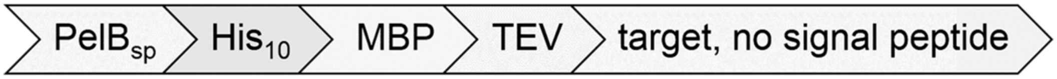


```
LOCUS       pelB-MBP-P66\DTH        8297 bp    DNA     circular      9-OCT-2018
DEFINITION
SOURCE
  ORGANISM  other sequences; artificial sequences; vectors.
COMMENT
FEATURES             Location/Qualifiers
     source          1..8297
                     /organism="pelB-MBP"
                     /mol_type="other DNA"
                     /vntifkey="98"
     gene            complement(561..1376)
                     /gene="KanR2"
                     /vntifkey="60"
     rep_origin      1482..2101
                     /vntifkey="33"
                     /label=pBR322_origin
     misc_feature    complement(2516..2707)
                     /vntifkey="21"
                     /label=ROP
     gene            complement(3179..3454)
                     /gene="tet (611 - 336)"
                     /vntifkey="60"
     misc_feature    complement(3516..4607)
                     /vntifkey="21"
                     /label=lacI
     CDS             complement(4348..4848)
                     /vntifkey="4"
                     /label=ORF\frame\3
     gene            complement(4699..4950)
                     /gene="tet (887 - 636)"
                     /vntifkey="60"
     promoter        4985..5003
                     /vntifkey="30"
                     /label=T7_promoter
     promoter        5003..5030
                     /vntifkey="30"
                     /label=lacO
     misc_feature    5048..5064
                     /vntifkey="21"
                     /label=RBS\(extended)
     terminator      8170..1
                     /vntifkey="43"
                     /label=T7_terminator
     misc_feature    5073..5138
                     /vntifkey="21"
                     /label=pelB\ss\(M1-A22\AAA24848;\PAMA*)
     CDS             complement(564..1376)
                     /vntifkey="4"
                     /label=KANAMYCIN-RESISTANCE
     misc_feature    5178..5207
                     /vntifkey="21"
                     /label=His10
     misc_feature    5214..6311
                     /vntifkey="21"
                     /label=MBP\(K27-T392\NP_290668)
     mutation        5114..5114
                     /vntifkey="62"
                     /label=C\to\T\removed\BseRI
     misc_feature    6321..6338
                     /vntifkey="21"
                     /label=TEV\protease\cleavage\ENFLYQ*A
     CDS             6339..8129
                     /vntifkey="4"
                     /label=P66\(aa\22-618)
     primer          4986..5002
                     /vntifkey="27"
                     /label=PRIMER\T7
     primer          complement(8215..8233)
                     /vntifkey="27"
                     /label=PRIMER\T7\Terminator
```

(continued)

Figure S4. Complete and annotated (a) DNA and (b) protein sequences for pelB-MBP-P66-ss. The other targets' clones have the same sequence aside from the sequence representing the mature form of the target, which is shown here in blue for P66: (**a**) bases 64-1854 of the full-length gene, and (**b**) amino acid residues 22-618 of the full-length protein.

```
     primer          complement(8255..8276)
                     /vntifkey="27"
                     /label=PRIMER\pET26\pelB\seqR
     primer          6188..6212
                     /vntifkey="27"
                     /label=PRIMER\MBPpelBseqF
BASE COUNT     2116 a      2195 c      2150 g      1836 t
ORIGIN
        1 ttggcgaatg ggacgcgccc tgtagcggcg cattaagcgc ggcgggtgtg gtggttacgc
       61 gcagcgtgac cgctacactt gccagcgccc tagcgcccgc tcctttcgct ttcttccctt
      121 cctttctcgc cacgttcgcc ggctttcccc gtcaagctct aaatcggggg ctccctttag
      181 ggttccgatt tagtgcttta cggcacctcg accccaaaaa acttgattag ggtgatggtt
      241 cacgtagtgg gccatcgccc tgatagacgg tttttcgccc tttgacgttg gagtccacgt
      301 tctttaatag tggactcttg ttccaaactg gaacaacact caaccctatc tcggtctatt
      361 cttttgattt ataagggatt ttgccgattt cggcctattg gttaaaaaat gagctgattt
      421 aacaaaaatt taacgcgaat tttaacaaaa tattaacgtt tacaatttca ggtggcactt
      481 ttcggggaaa tgtgcgcgga acccctattt gtttattttt ctaaatacat tcaaatatgt
      541 atccgctcat gaattaattc ttagaaaaac tcatcgagca tcaaatgaaa ctgcaattta
      601 ttcatatcag gattatcaat accatatttt tgaaaaagcc gtttctgtaa tgaaggagaa
      661 aactcaccga ggcagttcca taggatggca agatcctggt atcggtctgc gattccgact
      721 cgtccaacat caatacaacc tattaatttc ccctcgtcaa aaataaggtt atcaagtgag
      781 aaatcaccat gagtgacgac tgaatccggt gagaatggca aaagtttatg catttctttc
      841 cagacttgtt caacaggcca gccattacgc tcgtcatcaa aatcactcgc atcaaccaaa
      901 ccgttattca ttcgtgattg cgcctgagcg agacgaaata cgcgatcgct gttaaaagga
      961 caattacaaa caggaatcga atgcaaccgg cgcaggaaca ctgccagcgc atcaacaata
     1021 ttttcacctg aatcaggata ttcttctaat acctggaatg ctgttttccc ggggatcgca
     1081 gtggtgagta accatgcatc atcaggagta cggataaaat gcttgatggt cggaagaggc
     1141 ataaattccg tcagccagtt tagtctgacc atctcatctg taacatcatt ggcaacgcta
     1201 cctttgccat gtttcagaaa caactctggc gcatcgggct tcccatacaa tcgatagatt
     1261 gtcgcacctg attgcccgac attatcgcga gcccatttat acccatataa atcagcatcc
     1321 atgttggaat ttaatcgcgg cctagagcaa gacgtttccc gttgaatatg gctcataaca
     1381 ccccttgtat tactgtttat gtaagcagac agttttattg ttcatgacca aaatccctta
     1441 acgtgagttt tcgttccact gagcgtcaga ccccgtagaa aagatcaaag gatcttcttg
     1501 agatcctttt tttctgcgcg taatctgctg cttgcaaaca aaaaaaccac cgctaccagc
     1561 ggtggtttgt ttgccggatc aagagctacc aactcttttt ccgaaggtaa ctggcttcag
     1621 cagagcgcag ataccaaata ctgtccttct agtgtagccg tagttaggcc accacttcaa
     1681 gaactctgta gcaccgccta catacctcgc tctgctaatc ctgttaccag tggctgctgc
     1741 cagtggcgat aagtcgtgtc ttaccgggtt ggactcaaga cgatagttac cggataaggc
     1801 gcagcggtcg ggctgaacgg ggggttcgtg cacacagccc agcttggagc gaacgaccta
     1861 caccgaactg agatacctac agcgtgagct atgagaaagc gccacgcttc ccgaagggag
     1921 aaaggcggac aggtatccgg taagcggcag ggtcggaaca ggagagcgca cgagggagct
     1981 tccaggggga aacgcctggt atctttatag tcctgtcggg tttcgccacc tctgacttga
     2041 gcgtcgattt ttgtgatgct cgtcaggggg gcggagccta tggaaaaacg ccagcaacgc
     2101 ggccttttta cggttcctgg ccttttgctg gccttttgct cacatgttct ttcctgcgtt
     2161 atcccctgat tctgtggata accgtattac cgcctttgag tgagctgata ccgctcgccg
     2221 cagccgaacg accgagcgca gcgagtcagt gagcgaggaa gcggaagagc gcctgatgcg
     2281 gtattttctc cttacgcatc tgtgcggtat ttcacaccgc atatatggtg cactctcagt
     2341 acaatctgct ctgatgccgc atagttaagc cagtatacac tccgctatcg ctacgtgact
     2401 gggtcatggc tgcgccccga cacccgccaa cacccgctga cgcgccctga cgggcttgtc
     2461 tgctcccggc atccgcttac agacaagctg tgaccgtctc cgggagctgc atgtgtcaga
     2521 ggttttcacc gtcatcaccg aaacgcgcga ggcagctgcg gtaaagctca tcagcgtggt
     2581 cgtgaagcga ttcacagatg tctgcctgtt catccgcgtc cagctcgttg agtttctcca
     2641 gaagcgttaa tgtctggctt ctgataaagc gggccatgtt aagggcggtt ttttcctgtt
     2701 tggtcactga tgcctccgtg taagggggat ttctgttcat gggggtaatg ataccgatga
     2761 aacgagagag gatgctcacg atacgggtta ctgatgatga acatgcccgg ttactggaac
     2821 gttgtgaggg taaacaactg gcggtatgga tgcggcggga ccagagaaaa atcactcagg
     2881 gtcaatgcca gcgcttcgtt aatacagatg taggtgttcc acagggtagc cagcagcatc
     2941 ctgcgatgca gatccggaac ataatggtgc agggcgctga cttccgcgtt tccagacttt
     3001 acgaaacacg gaaaccgaag accattcatg ttgttgctca ggtcgcagac gttttgcagc
     3061 agcagtcgct tcacgttcgc tcgcgtatcg gtgattcatt ctgctaacca gtaaggcaac
     3121 cccgccagcc tagccgggtc ctcaacgaca ggagcacgat catgcgcacc cgtggggccg
     3181 ccatgccggc gataatggcc tgcttctcgc cgaaacgttt ggtggcggga ccagtgacga
     3241 aggcttgagc gagggcgtgc aagattccga ataccgcaag cgacaggccg atcatcgtcg
     3301 cgctccagcg aaagcggtcc tcgccgaaaa tgacccagag cgctgccggc acctgtccta
     3361 cgagttgcat gataaagaag acagtcataa gtgcggcgac gatagtcatg ccccgcgccc
     3421 accggaagga gctgactggg ttgaaggctc tcaagggcat cggtcgagat cccggtgcct
     3481 aatgagtgag ctaacttaca ttaattgcgt tgcgctcact gcccgctttc cagtcgggaa
     3541 acctgtcgtg ccagctgcat taatgaatcg gccaacgcgc ggggagaggc ggtttgcgta
     3601 ttgggcgcca gggtggtttt tcttttcacc agtgagacgg gcaacagctg attgcccttc
     3661 accgcctggc cctgagagag ttgcagcaag cggtccacgc tggtttgccc cagcaggcga
     3721 aaatcctgtt tgatggtggt taacggcggg atataacatg agctgtcttc ggtatcgtcg
     3781 tatcccacta ccgagatatc cgcaccaacg cgcagcccgg actcggtaat ggcgcgcatt
     3841 gcgcccagcg ccatctgatc gttggcaacc agcatcgcag tgggaacgat gccctcattc
     3901 agcatttgca tggtttgttg aaaaccggac atggcactcc agtcgccttc ccgttccgct
     3961 atcggctgaa tttgattgcg agtgagatat ttatgccagc cagccagacg cagacgcgcc
     4021 gagacagaac ttaatgggcc cgctaacagc gcgatttgct ggtgacccaa tgcgaccaga
     4081 tgctccacgc ccagtcgcgt accgtcttca tgggagaaaa taatactgtt gatgggtgtc
     4141 tggtcagaga catcaagaaa taacgccgga acattagtgc aggcagcttc cacagcaatg
     4201 gcatcctggt catccagcgg atagttaatg atcagcccac tgacgcgttg cgcgagaaga
```

(continued)

Figure S4 (continued).

```
4261 ttgtgcaccg ccgctttaca ggcttcgacg ccgcttcgtt ctaccatcga caccaccacg
4321 ctggcaccca gttgatcggc gcgagattta atcgccgcga caatttgcga cggcgcgtgc
4381 agggccagac tggaggtggc aacgccaatc agcaacgact gtttgcccgc cagttgttgt
4441 gccacgcggt tgggaatgta attcagctcc gccatcgccg cttccacttt ttcccgcgtt
4501 ttcgcagaaa cgtggctggc ctggttcacc acgcgggaaa cggtctgata agagacaccg
4561 gcatactctg cgacatcgta taacgttact ggtttcacat tcaccaccct gaattgactc
4621 tcttccgggc gctatcatgc cataccgcga aaggttttgc gccattcgat ggtgtccggg
4681 atctcgacgc tctcccttat gcgactcctg cattaggaag cagcccagta gtaggttgag
4741 gccgttgagc accgccgccg caaggaatgg tgcatgcaag gagatggcgc ccaacagtcc
4801 cccggccacg gggcctgcca ccatacccac gccgaaacaa gcgctcatga gcccgaagtg
4861 gcgagcccga tcttccccat cggtgatgtc ggcgatatag gcgccagcaa ccgcacctgt
4921 ggcgccggtg atgccggcca cgatgcgtcc ggcgtagagg atcgagatct cgatcccgcg
4981 aaattaatac gactcactat aggggaattg tgagcggata acaattcccc tctagaaata
5041 attttgttta actttaagaa ggagatatac atatgaaata cctgctgccg accgctgctg
5101 ctggtctgct gcttctcgct gcccagccgg cgatggccat ggatatcgga attaattcgg
5161 atccgaattc gagctcacat caccatcacc atcaccatca ccatcacccc atgaaaatcg
5221 aagaaggtaa actggtaatc tggattaacg gcgataaagg ctataacggt ctcgctgaag
5281 tcggtaagaa attcgagaaa gataccggaa ttaaagtcac cgttgagcat ccggataaac
5341 tggaagagaa attcccacag gttgcggcaa ctggcgatgg ccctgacatt atcttctggg
5401 cacacgaccg ctttggtggc tacgctcaat ctggcctgtt ggctgaaatc accccggaca
5461 aagcgttcca ggacaagctg tatccgttta cctgggatgc cgtacgttac aacggcaagc
5521 tgattgctta cccgatcgct gttgaagcgt tatcgctgat ttataacaaa gatctgctgc
5581 cgaacccgcc aaaaacctgg gaagagatcc cggcgctgga taaagaactg aaagcgaaag
5641 gtaagagcgc gctgatgttc aacctgcaag aaccgtactt cacctggccg ctgattgctg
5701 ctgacggggg ttatgcgttc aagtatgaaa acggcaagta cgacattaaa gacgtgggcg
5761 tggataacgc tggcgcgaaa gcgggtctga ccttcctggt tgacctgatt aaaaacaaac
5821 acatgaatgc agacaccgat tactccatcg cagaagctgc ctttaataaa ggcgaaacag
5881 cgatgaccat caacggcccg tgggcatggt ccaacatcga caccagcaaa gtgaattatg
5941 gtgtaacggt actgccgacc ttcaagggtc aaccatccaa accgttcgtt ggcgtgctga
6001 gcgcaggtat taacgccgcc agtccgaaca aagagctggc aaaagagttc ctcgaaaact
6061 atctgctgac tgatgaaggt ctggaagcgg ttaataaaga caaaccgctg ggtgccgtag
6121 cgctgaagtc ttacgaggaa gagttggcga aagatccacg tattgccgcc actatggaaa
6181 acgcccagaa aggtgaaatc atgccgaaca tcccgcagat gtccgctttc tggtatgccg
6241 tgcgtactgc ggtgatcaac gccgccagcg gtcgtcagac tgtcgatgaa gccctgaaag
6301 acgcgcagac tccgggtagc gaaaacctgt acttccaagc ggatgcgctg aaagaaaagg
6361 acatcttcaa aattaacccg tggatgccga ccttcggttt tgagaacacc agcgaatttc
6421 gtctggacat ggatgagctg gtgccgggct tcgaaaacaa aagcaagatc accattaaac
6481 tgaagccgtt tgaggcgaac ccggaactgg gcaaggacga tccgttcagc gcgtacatta
6541 aagtggagga tctggcgctg aaggcggaag gtaagaaagg cgaccaattc aaaatcgatg
6601 ttggcgacat taccgcgcag atcaacatgt acgacttctt tatcaagatt agcaccatga
6661 ccgacttcga ttttaacaaa gagagcctgt tcagctttgc gccgatgacc ggttttaaga
6721 gcacctacta tggcttcccg agcaacgatc gtgcggttcg tggcaccatc ctggcgcgtg
6781 gcaccagcaa aaacatcggc accattcaac tgggctacaa actgccgaag ctggacctga
6841 cctttgcgat cggtggcacc ggcaccggta accgtaacca ggaaaacgac aaggataccc
6901 cgtacaacaa aacctatcag ggcattctgt atggtatcca agcgacctgg aaaccgatta
6961 agaacctgct ggatcagaac gaggacacca agagcgtgat cgcggagacc ccgtttgaac
7021 tgaacttcgg tctgagcggc gcgtatggta acgaaacctt taacaacagc agcatcacct
7081 acagcctgaa agataagagc gtggttggta acgacctgct gagcccgacc ctgagcaaca
7141 gcgcgattct ggcgagcttc ggtgcgaaat ataagctggg cctgaccaaa attaacgata
7201 agaacaccta cctgatcctg cagatgggca ccgattttgg tatcgacccg ttcgcgagcg
7261 actttagcat tttcggtcac atcagcaagg cggcgaactt caagaaagag accccgagcg
7321 atccgaacaa gaaagcggaa atctttgacc cgaacggtaa cgcgctgaat ttcagcaaaa
7381 acaccgagct gggcattgcg tttagcaccg gcgcgagcat cggtttcgcg tggaacaaag
7441 ataccggcga gaaggaaagc tgggcgatca agggcagcga tagctacagc acccgtctgt
7501 ttggtgaaca agacaagaaa agcggcgtgg cgctgggtat tagctatggc cagaacctgt
7561 accgtagcaa agacaccgag aaacgtctga agaccattag cgaaaacgcg ttccaaagcc
7621 tgaacgttga gatcagcagc tatgaagata acaagaaagg tatcatcaac ggtctgggct
7681 ggatcaccag cattggcctg tatgacattc tgcgtcagaa aagcgtggag aactacccga
7741 ccaccatcag cagcaccacc gagaacaacc agaccgaaca aagcagcacc agcaccaaga
7801 ccaccacccc gaacctgacc ttcgaggatg cgatgaaact gggtctggcg ctgtacctgg
7861 actatgcgat cccgattgcg agcatcagca ccgaagcgta cgtggttccg tatattggtg
7921 cgtacatcct gggcccgagc aacaagctga gcagcgatgc gaccaaaatc tacctgaaga
7981 ccggtctgag cctggagaaa ctgattcgtt ttaccaccat cagcctgggc tgggacagca
8041 acaacatcat tgaactggcg aacaagaaca ccaacaacgc ggcgattggt agcgcgtttc
8101 tgcaattcaa aatcgcgtac agcggtagct aataactcga gcaccaccac caccaccact
8161 gagatccggc tgctaacaaa gcccgaaagg aagctgagtt ggctgctgcc accgctgagc
8221 aataactagc ataacccctt ggggcctcta aacgggtctt gaggggtttt ttgctgaaag
8281 gaggaactat atccgga
//
```

Mature P66

(continued)

Figure S4 (continued).

b

```
LOCUS       Translation\of\pelB-MBP-P66-ss\DTH        1019 aa                         1-NOV-2018
DEFINITION  Translation of a fragment of pelB-MBP-P66 DTH.
KEYWORDS    TRANSLATED.
SOURCE
  ORGANISM
COMMENT
FEATURES             Location/Qualifiers
     Region          1..22
                     /vntifkey="200"
                     /label=pelB\signal\sequence\(aa1-22\AAA24848)\QPAMA*
     Site            36..45
                     /vntifkey="264"
                     /label=His10
     Region          48..413
                     /vntifkey="1005"
                     /label=MBP\(K27-T392\of\NP_290668)
     Region          423..1019
                     /vntifkey="1000"
                     /label=P66\(aa\22-618)
     Site            417..422
                     /vntifkey="221"
                     /label=TEV\protease\cleavage\ENLYFQ*
ORIGIN
        1 mkyllptaaa glllaaqpa mamdiginsd pnssshhhhh hhhhhpmkie egklviwing
       61 dkgynglaev gkkfekdtgi kvtvehpdkl eekfpqvaat gdgpdiifwa hdrfggyaqs
      121 gllaeitpdk afqdklypft wdavryngkl iaypiaveal sliynkdllp nppktweeip
      181 aldkelkakg ksalmfnlqe pyftwpliaa dggyafkyen gkydikdvgv dnagakaglt
      241 flvdliknkh mnadtdysia eaafnkgeta mtingpwaws nidtskvnyg vtvlptfkgq
      301 pskpfvgvls aginaaspnk elakefleny lltdegleav nkdkplgava lksyeeelak
      361 dpriaatmen aqkgeimpni pqmsafwyav rtavinaasg rqtvdealkd aqtpgsenly
      421 fqadalkekd ifkinpwmpt fgfentsefr ldmdelvpgf enkskitikl kpfeanpelg
      481 kddpfsayik vedlalkaeg kkgdqfkidv gditaqinmy dffikistmt dfdfnkeslf
      541 sfapmtgfks tyygfpsndr avrgtilarg tsknigtiql gyklpkldlt faiggtgtgn
      601 rnqendkdtp ynktyqgily giqatwkpik nlldqnedtk sviaetpfel nfglsgaygn
      661 etfnnssity slkdksvvgn dllsptlsns ailasfgaky klgltkindk ntylilqmgt
      721 dfgidpfasd fsifghiska anfkketpsd pnkkaeifdp ngnalnfskn telgiafstg
      781 asigfawnkd tgekeswaik gsdsystrlf geqdkksgva lgisygqnly rskdtekrlk
      841 tisenafqsl nveissyedn kkgiinglgw itsiglydil rqksvenypt tissttennq
      901 teqsststkt ttpnltfeda mklglalyld yaipiasist eayvvpyiga yilgpsnkls
      961 sdatkiylkt glsleklirf ttislgwdsn niielanknt nnaaigsafl qfkiaysgs
//
```

Mature P66

Figure S4 (continued).

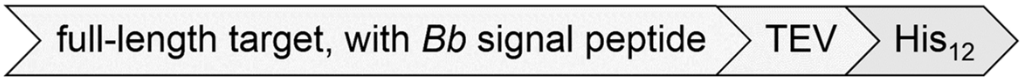

* 95°C for 5 min
** 37°C for 1 h

a

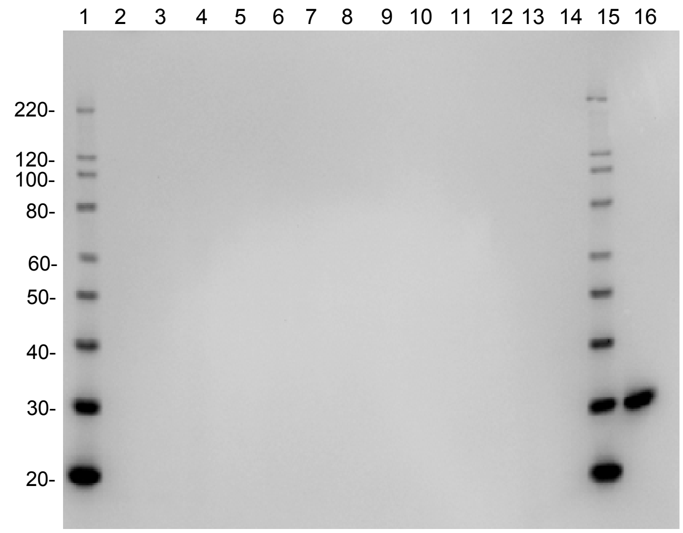

kDa	Lane & sample
	**1** MW
22	**2** P13-His$_{12}$, C43(DE3), TALON purified sample *
22	**3** P13-His$_{12}$, C43(DE3) *
22	**4** P13-His$_{12}$, C43(DE3) **
22	**5** P13-His$_{12}$, BL21(DE3) *
22	**6** P13-His$_{12}$, BL21(DE3) **
22	**7** P13-His$_{12}$, BL21-AI *
22	**8** P13-His$_{12}$, BL21-AI **
47	**9** BB0323-His$_{12}$, C43(DE3) *
47	**10** BB0323-His$_{12}$, C43(DE3) **
41	**11** DipA-His$_{12}$, C43(DE3), TALON purified sample *
41	**12** DipA-His$_{12}$, C43(DE3) *
41	**13** DipA-His$_{12}$, C43(DE3) **
41	**14** DipA-His$_{12}$, BL21(DE3) *
	**15** MW
	**16** GFP-His$_6$ (20 ng)

b

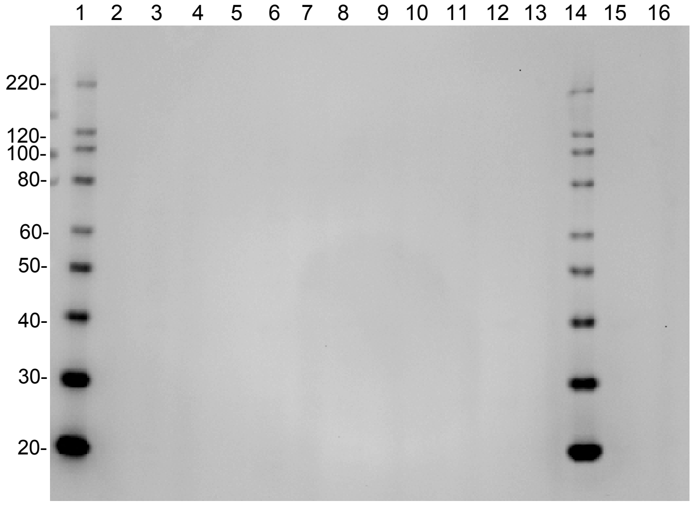

kDa	Lane & sample
	**1** MW
41	**2** DipA-His$_{12}$, BL21(DE3) **
41	**3** DipA-His$_{12}$, BL21-AI *
41	**4** DipA-His$_{12}$, BL21-AI **
25	**5** BB0405-His$_{12}$, C43(DE3), TALON purified sample *
25	**6** BB0405-His$_{12}$, C43(DE3) *
25	**7** BB0405-His$_{12}$, C43(DE3) **
25	**8** BB0405-His$_{12}$, BL21(DE3) *
25	**9** BB0405-His$_{12}$, BL21(DE3) **
25	**10** BB0405-His$_{12}$, BL21-AI *
25	**11** BB0405-His$_{12}$, BL21-AI **
25	**12** BB0405-His$_{12}$, KTD101(DE3) *
25	**13** BB0405-His$_{12}$, KTD101(DE3) **
	**14** MW
20	**15** RevA-His$_{12}$, C43(DE3) *
20	**16** RevA-His$_{12}$, C43(DE3) **

c

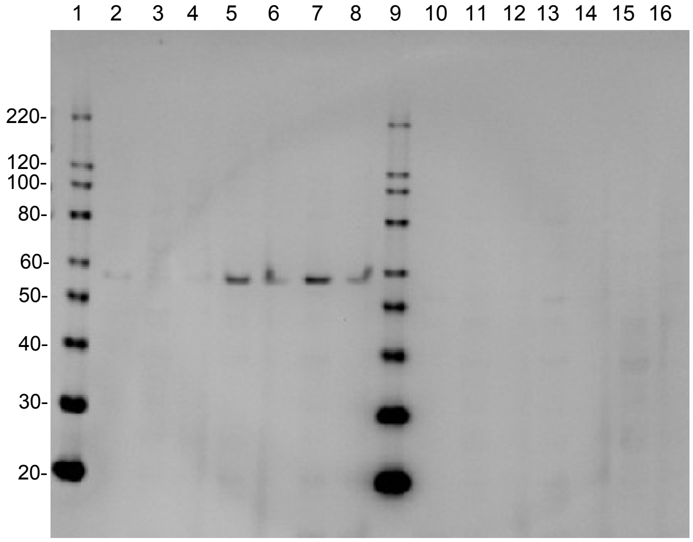

kDa	Lane & sample
	**1** MW
50	**2** BBA57-His$_{12}$, C43(DE3), TALON purified sample *
50	**3** BBA57-His$_{12}$, C43(DE3) *
50	**4** BBA57-His$_{12}$, C43(DE3) **
50	**5** BBA57-His$_{12}$, BL21(DE3) *
50	**6** BBA57-His$_{12}$, BL21(DE3) **
50	**7** BBA57-His$_{12}$, KTD101(DE3) *
50	**8** BBA57-His$_{12}$, KTD101(DE3) **
	**9** MW
55	**10** HtrA-His$_{12}$, C43(DE3) *
55	**11** HtrA-His$_{12}$, C43(DE3) *
55	**12** HtrA-His$_{12}$, C43(DE3) **
55	**13** HtrA-His$_{12}$, KTD101(DE3) *
55	**14** HtrA-His$_{12}$, KTD101(DE3) **
32	**15** BB0238-His$_{12}$, C43(DE3) *
32	**16** BB0238-His$_{12}$, C43(DE3) **

Figure S5. Expression in various *E. coli* strains of C-terminally His$_{12}$–tagged targets as determined by Western blot (anti-His). Cultures were grown at 37°C to an OD_{600} of 0.5, induced with 0.2% arabinose (for BL21-AI) or 0.5 mM IPTG (for all other strains), and harvested after 3 hours. Prior to gel loading, the samples were heated in loading buffer at (*) 95°C for 5 min or (**) 37°C for 1 h, since conditions can vary for the complete denaturation of membrane proteins. However, no differences in protein migration was observed here for these denaturation conditions. For TALON purified samples, cells from 3 mL cultures (**a**, lanes 2 and 11; **b**, lane 5; **b**, lane 2) were lysed in buffer with 1% DDM and centrifuged, and the supernatant was subjected to His-tag purification using cobalt affinity magnetic beads (Supplementary Methods). Each lane contains sample from the equivalent of 10 µL of cell culture, except TALON purified samples contain sample from 300 µL of culture.
Abbreviations: MW, molecular weight marker.

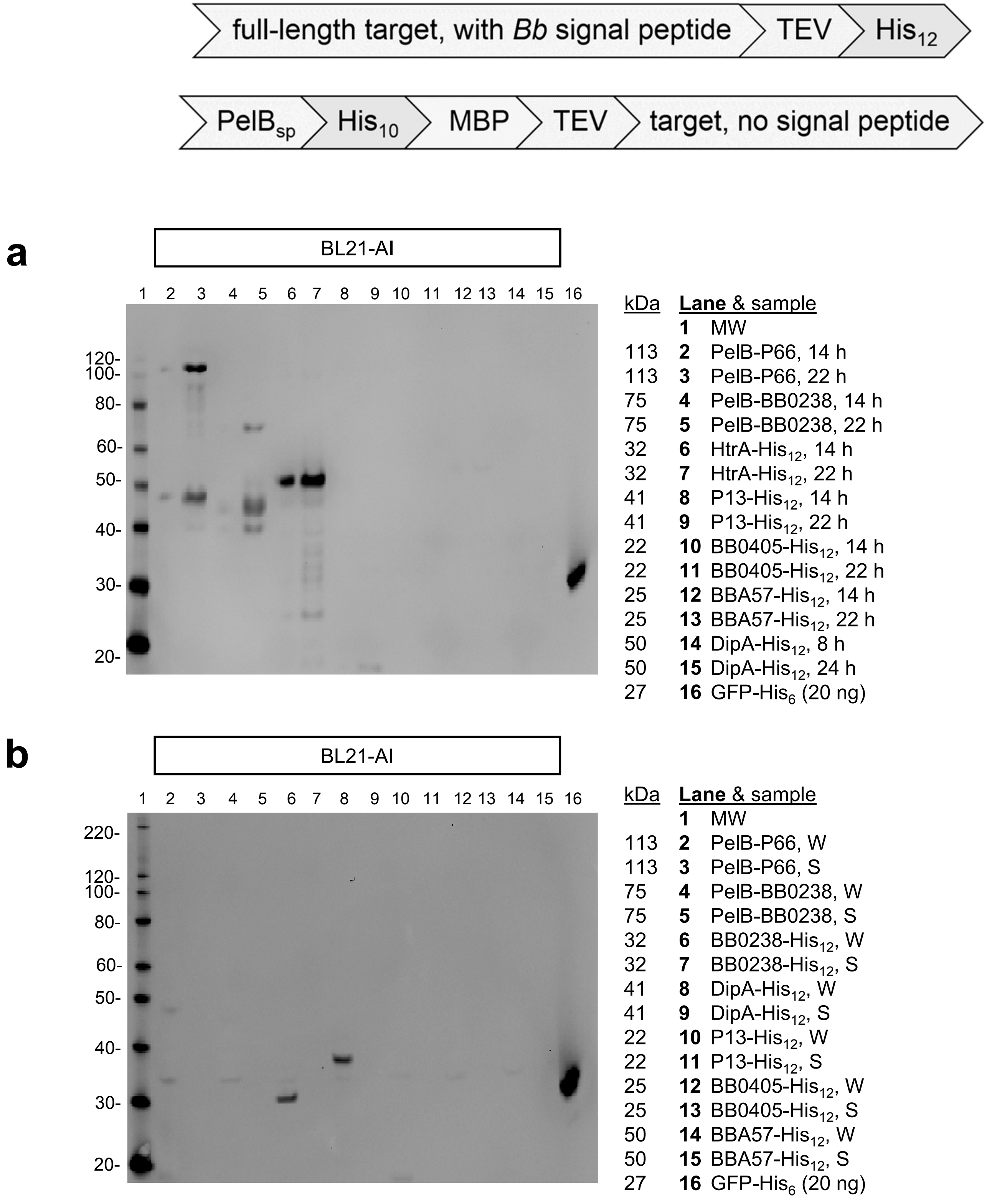

Figure S6. Complete Western blot images are shown here for (a) Figure 1d and (b) Figure 1e. Expression conditions were as described in Figure 1. (**a**) Note that BBA57-His$_{12}$ (lanes 12-13) was not expressed in M9 minimal media under induction with 0.1% arabinose and a temperature shift to 25°C at induction. (**b**) Note that there was no visible expression of BBA57-His$_{12}$ (lanes 14-15), possibly due to the temperature shift to 18°C at induction and/or to an induction at an OD_{600} of 1.0, since BBA57-His$_{12}$ was visible under similar conditions when induced at an OD_{600} near 0.5 with either no temperature shift (Fig. 1c) or a shift to 25C (Fig. 2).
Abbreviations: W, whole culture before harvesting the cells by centrifugation; S, culture supernatant, after centrifugation and removal of the cells.

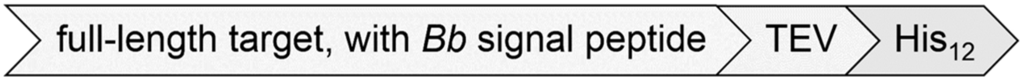

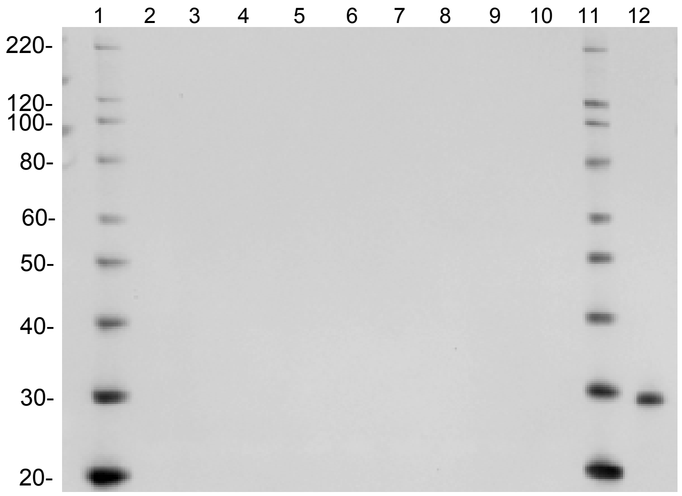

kDa	**Lane** & sample		
	**1**	MW	
25	**2**	BB0405-His_{12}	100 µM rhamnose
25	**3**	BB0405-His_{12}	500 µM rhamnose
25	**4**	BB0405-His_{12}	1000 µM rhamnose
41	**5**	DipA-His_{12}	100 µM rhamnose
41	**6**	DipA-His_{12}	500 µM rhamnose
41	**7**	DipA-His_{12}	1000 µM rhamnose
50	**8**	BBA57-His_{12}	100 µM rhamnose
50	**9**	BBA57-His_{12}	500 µM rhamnose
50	**10**	BBA57-His_{12}	1000 µM rhamnose
	**11**	MW	
27	**12**	GFP-His_6 (20 ng)	

Figure S7. Expression in *E. coli* Lemo21(DE3) of targets containing a C-terminal His_{12}-tag. Shown is a Western blot (anti-His) of total cellular protein from 10 µL of culture that was grown at 37°C to an OD_{600} of 0.5, then induced with 400 µM IPTG, and incubated for 5 h before harvesting the cells. The indicated concentrations of rhamnose were added to the media prior to inoculation.

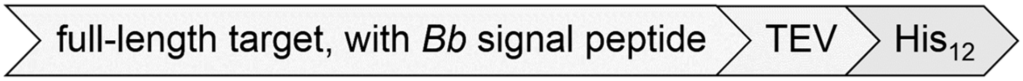

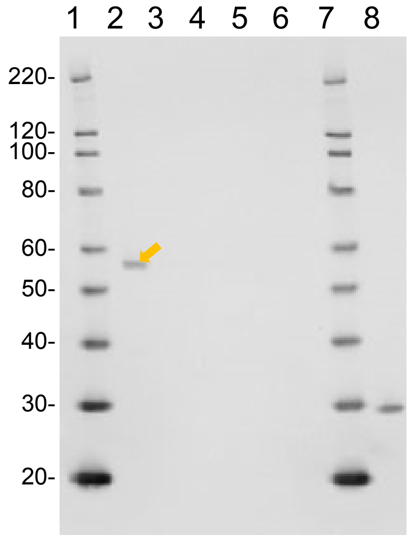

kDa	Lane & sample		
	**1**	MW	
47.5	**2**	BBA57-His_{12}	BL21-AI
47.5	**3**	BBA57-His_{12}	KTD101(DE3)
47.5	**4**	BBA57-His_{12}	BL21(DE3)
51.4	**5**	HtrA-His_{12}	BL21-AI
44.5	**6**	BB0323-His_{12}	BL21-AI
	**7**	MW	
27.7	**8**	GFP-His_6 (20 ng)	

Figure S8. *Bb* BBA57-His_{12} is translocated to the membrane of *E. coli*. Shown is a Western blot (anti-His) of the detergent-soluble (1% DDM) protein fraction from expressed targets in Figure 1c and Figure S5c. Each lane contains sample from the equivalent of 75 µL of culture. The yellow arrow indicates detergent-soluble BBA57-His_{12}. Purified GFP-His_6 is a positive control (lane 8).

Abbreviations: *Bb*, *Borrelia burgdorferi*; DDM, *n*-dodecyl β-D-maltoside; MW, molecular weight marker.

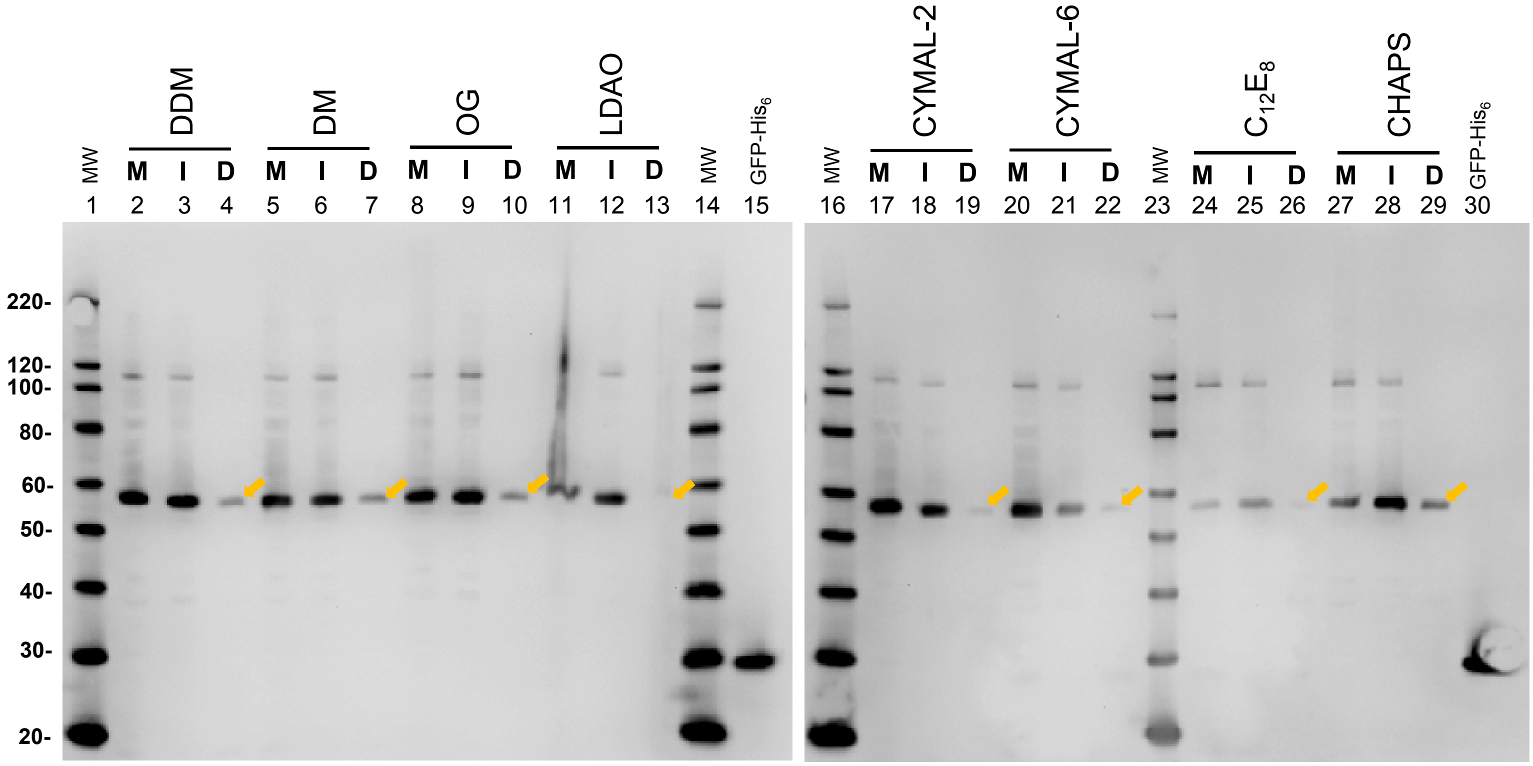

Figure S9. Full detergent screen of full-length, C-terminally His-tagged BBA57 solubilized from a cell membrane fraction of *E. coli*. Shown are Western immunoblots (anti-His). Samples are as described in Figure 2 except that each lane contains sample from 23.4 µL of culture.
Abbreviations: MW, molecular weight; sup., supernatant.

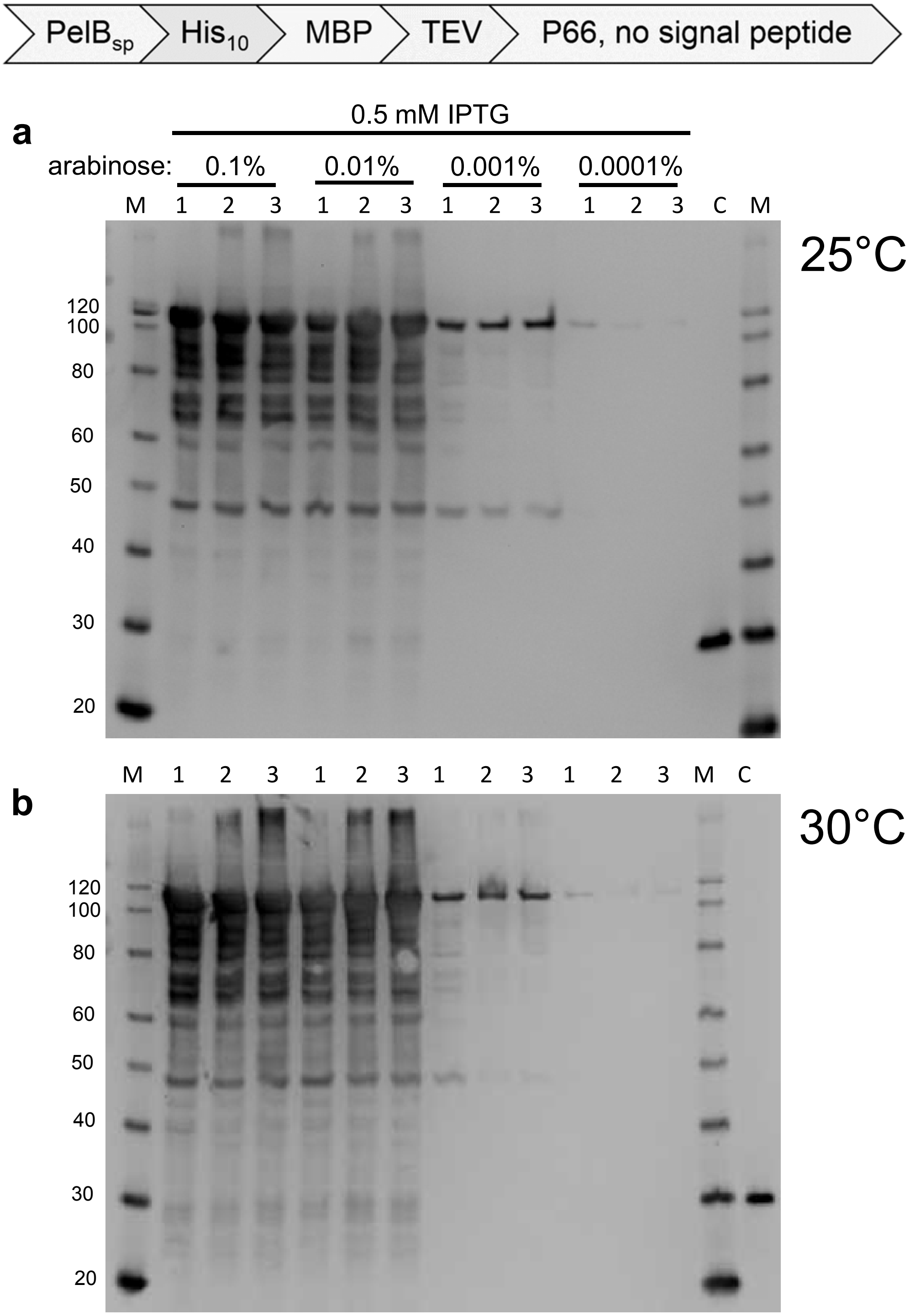

Figure S10. Expression of PelB-P66 in *E. coli* BL21-AI. Shown are Western blots (anti-His) of total cellular protein from *E. coli* culture expressing PelB-P66 (110 kDa). Cultures were grown at 37°C until reaching an OD_{600} of 0.7, induced with 0.1-0.0001% arabinose plus 0.5 mM IPTG, and harvested after 8 h (lanes 1), 20 h (lanes 2) or 24 h (lanes 3). At induction, the temperature was reduced to (**a**) 25°C or (**b**) 30°C. Each lane contains cells from 5 µL of culture. Lanes C contain a Western positive control, 20 ng of purified GFP-His$_6$.

Abbreviations: M, molecular weight marker.

```
LOCUS       pRSET-TEV-12His\DTH        3281 bp    DNA     circular      9-OCT-2018
DEFINITION  T7 driven prokaryotic expression vector (Invitrogen).
SOURCE
  ORGANISM
COMMENT
FEATURES             Location/Qualifiers
     promoter        20..39
                     /vntifkey="30"
                     /label=T7\promoter
     rep_origin      840..1295
                     /vntifkey="33"
                     /label=f1\ori
     CDS             1426..2286
                     /vntifkey="4"
                     /label=AMP-R
     promoter        1327..1425
                     /vntifkey="30"
                     /label=bla\pr
     rep_origin      2431..3104
                     /vntifkey="33"
                     /label=pUC\ori
     RBS             85..92
                     /vntifkey="32"
                     /label=RBS
     terminator      640..769
                     /vntifkey="43"
                     /label=T7\term
     misc_feature    85..121
                     /vntifkey="21"
                     /label=F\infuse\stuffer\BseRI
     primer          20..39
                     /vntifkey="27"
                     /label=PRIMER\T7\(SOLS)
     primer          complement(682..700)
                     /vntifkey="27"
                     /label=Primer\T7\terminator\(SOLS)
     primer          complement(725..746)
                     /vntifkey="27"
                     /label=PRIMER\pET26\pelB\seqR
BASE COUNT      836 a       833 c       806 g       806 t
ORIGIN
        1 gatctcgatc ccgcgaaatt aatacgactc actataggga gaccacaacg gtttccctct
       61 agaaataatt ttgtttaact ttaagaagga gatatacata gatctgtcgc tcctcatgta
      121 gacaccgaca ctgtgaacac cgccatcctg ggcgtttcaa ggtccggcaa aggcgagaca
      181 atcgtagtgc caatgatcga caatctgagc cgcgcaaaga accagtctag catggtcgtg
      241 aacgacccca agggcgagct ctactctgcc agcaaggaaa cacttgagaa acgcggatac
      301 gacgtgcagg tgctgaacat cctggatccc ctgcagggca tgagttacaa cccactccag
      361 ctcgtgatcg acgcctgggt gaacggcgac gaccaggagg ctgcaaagag ggcaaacaca
      421 ctgactttct cactgtacaa caacccaaat gctggcgaca acgctttctt taacaccagc
      481 gcccagaatg ctatcaacgg aatcatcctc gctatgagga ggcagaagct tgaaaatctt
      541 tattttcaag gtcaccatca tcaccaccat caccatcatc accaccatta ataaaagggc
      601 gaattccagc acactggcgg ccgttactag tggatccggc tgctaacaaa gcccgaaagg
      661 aagctgagtt ggctgctgcc accgctgagc aataactagc ataacccctt ggggcctcta
      721 aacgggtctt gaggggtttt ttgctgaaag gaggaactat atccggatct ggcgtaatag
      781 cgaagaggcc cgcaccgatc gcccttccca acagttgcgc agcctgaatg gcgaatggga
      841 cgcgccctgt agcggcgcat taagcgcggc gggtgtggtg gttacgcgca gcgtgaccgc
      901 tacacttgcc agcgccctag cgcccgctcc tttcgctttc ttcccttcct ttctcgccac
      961 gttcgccggc tttccccgtc aagctctaaa tcgggggctc cctttagggt tccgatttag
     1021 tgctttacgg cacctcgacc ccaaaaaact tgattagggt gatggttcac gtagtgggcc
     1081 atcgccctga tagacggttt ttcgcccttt gacgttggag tccacgttct ttaatagtgg
     1141 actcttgttc caaactggaa caacactcaa ccctatctcg gtctattctt ttgatttata
     1201 agggattttg ccgatttcgg cctattggtt aaaaaatgag ctgatttaac aaaaatttaa
     1261 cgcgaatttt aacaaaatat taacgcttac aatttaggtg gcacttttcg gggaaatgtg
     1321 cgcggaaccc ctatttgttt atttttctaa atacattcaa atatgtatcc gctcatgaga
     1381 caataaccct gataaatgct tcaataatat tgaaaaagga agagtatgag tattcaacat
     1441 ttccgtgtcg cccttattcc cttttttgcg gcattttgcc ttcctgtttt tgctcaccca
     1501 gaaacgctgg tgaaagtaaa agatgctgaa gatcagttgg gtgcacgagt gggttacatc
     1561 gaactggatc tcaacagcgg taagatcctt gagagttttc gccccgaaga acgttttcca
     1621 atgatgagca cttttaaagt tctgctatgt ggcgcggtat tatcccgtat tgacgccggg
     1681 caagagcaac tcggtcgccg catacactat tctcagaatg acttggttga gtactcacca
     1741 gtcacagaaa agcatcttac ggatggcatg acagtaagag aattatgcag tgctgccata
     1801 accatgagtg ataacactgc ggccaactta cttctgacaa cgatcggagg accgaaggag
     1861 ctaaccgctt ttttgcacaa catgggggat catgtaactc gccttgatcg ttgggaaccg
     1921 gagctgaatg aagccatacc aaacgacgag cgtgacacca cgatgcctgt agcaatggca
     1981 acaacgttgc gcaaactatt aactggcgaa ctacttactc tagcttcccg gcaacaatta
     2041 atagactgga tggaggcgga taaagttgca ggaccacttc tgcgctcggc ccttccggct
     2101 ggctggttta ttgctgataa atctggagcc ggtgagcgtg ggtctcgcgg tatcattgca
     2161 gcactggggc cagatggtaa gccctcccgt atcgtagtta tctacacgac ggggagtcag
     2221 gcaactatgg atgaacgaaa tagacagatc gctgagatag gtgcctcact gattaagcat
     2281 tggtaactgt cagaccaagt ttactcatat atactttaga ttgatttaaa acttcatttt
     2341 taatttaaaa ggatctaggt gaagatcctt tttgataatc tcatgaccaa aatcccttaa
     2401 cgtgagtttt cgttccactg agcgtcagac cccgtagaaa agatcaaagg atcttcttga
     2461 gatccttttt ttctgcgcgt aatctgctgc ttgcaaacaa aaaaaccacc gctaccagcg
     2521 gtggtttgtt tgccggatca agagctacca actctttttc cgaaggtaac tggcttcagc
     2581 agagcgcaga taccaaatac tgttcttcta gtgtagccgt agttaggcca ccacttcaag
     2641 aactctgtag caccgcctac atacctcgct ctgctaatcc tgttaccagt ggctgctgcc
     2701 agtggcgata agtcgtgtct taccgggttg gactcaagac gatagttacc ggataaggcg
     2761 cagcggtcgg gctgaacggg gggttcgtgc acacagccca gcttggagcg aacgacctac
     2821 accgaactga gatacctaca gcgtgagcta tgagaaagcg ccacgcttcc cgaagggaga
     2881 aaggcggaca ggtatccggt aagcggcagg gtcggaacag gagagcgcac gagggagctt
     2941 ccagggggaa acgcctggta tctttatagt cctgtcgggt ttcgccacct ctgacttgag
     3001 cgtcgatttt tgtgatgctc gtcagggggg cggagcctat ggaaaaacgc cagcaacgcg
     3061 gcctttttac ggttcctggc cttttgctgg ccttttgctc acatgttctt tcctgcgtta
     3121 tcccctgatt ctgtggataa ccgtattacc gcctttgagt gagctgatac cgctcgccgc
     3181 agccgaacga ccgagcgcag cgagtcagtg agcgaggaag cggaagagcg cccaatacgc
     3241 aaaccgcctc tccccgcgcg ttggccgatt cattaatgca g
//
```

Figure S11. Complete and annotated DNA sequence for the empty parent vector pRSET-TEV-12His.

```
LOCUS       pelB-MBP\DTH        6932 bp    DNA     circular      9-OCT-2018
DEFINITION
SOURCE
  ORGANISM  other sequences; artificial sequences; vectors.
COMMENT
FEATURES             Location/Qualifiers
     source          1..6932
                     /organism="pet"
                     /mol_type="other DNA"
                     /vntifkey="98"
     gene            complement(561..1376)
                     /gene="KanR2"
                     /vntifkey="60"
     rep_origin      1482..2101
                     /vntifkey="33"
                     /label=pBR322_origin
     misc_feature    2500..2522
                     /vntifkey="21"
                     /label=pGEX_3_primer
     misc_feature    complement(2516..2707)
                     /vntifkey="21"
                     /label=ROP
     gene            complement(3179..3454)
                     /gene="tet (611 - 336)"
                     /vntifkey="60"
     misc_feature    complement(3516..4607)
                     /vntifkey="21"
                     /label=lacI
     CDS             complement(4348..4848)
                     /vntifkey="4"
                     /label=ORF\frame\3
     gene            complement(4699..4950)
                     /gene="tet (887 - 636)"
                     /vntifkey="60"
     misc_feature    4882..4901
                     /vntifkey="21"
                     /label=pBRrevBam_primer
     promoter        4985..5003
                     /vntifkey="30"
                     /label=T7_promoter
     promoter        5003..5030
                     /vntifkey="30"
                     /label=lacO
     misc_feature    5048..5064
                     /vntifkey="21"
                     /label=RBS\(extended)
     terminator      6805..1
                     /vntifkey="43"
                     /label=T7_terminator
     misc_feature    5073..5138
                     /vntifkey="21"
                     /label=pelB\ss\(M1-A22\AAA24848;\BseRI\removed;\PAMA*)
     CDS             complement(564..1376)
                     /vntifkey="4"
                     /label=KANAMYCIN-RESISTANCE
     misc_feature    5178..5207
                     /vntifkey="21"
                     /label=His10
     misc_feature    5214..6311
                     /vntifkey="21"
                     /label=MBP\(K27-T392\from\NP_290668)
     mutation        5114..5114
                     /vntifkey="62"
                     /label=C\to\T\change\removed\BseRI\site\from\pelB-ss
     misc_feature    6321..6338
                     /vntifkey="21"
                     /label=TEV\protease\cleavage\site
     primer          complement(6890..6911)
                     /vntifkey="27"
                     /label=PRIMER\pET26\pelB\seqR
     primer          4954..4975
                     /vntifkey="27"
                     /label=FC\sequencing\primer\pET-PPL\seq\1\F
     primer          complement(6807..6828)
                     /vntifkey="27"
                     /label=FC\sequencing\primer\pET-PPL\seq\1\R
     primer          5616..5640
                     /vntifkey="27"
                     /label=FC\sequencing\primer\pET-PPL\seq\2\F
     primer          6218..6238
                     /vntifkey="27"
                     /label=FC\primer\MBPFOR
     primer          6186..6209
                     /vntifkey="27"
                     /label=FC\primer\MBP\seq\F
     primer          6188..6212
                     /vntifkey="27"
                     /label=PRIMER\MBPpelBseqF
BASE COUNT     1709 a      1853 c      1788 g      1582 t
ORIGIN
        1 ttggcgaatg ggacgcgccc tgtagcggcg cattaagcgc ggcgggtgtg gtggttacgc
       61 gcagcgtgac cgctacactt gccagcgccc tagcgcccgc tcctttcgct ttcttccctt
      121 cctttctcgc cacgttcgcc ggctttcccc gtcaagctct aaatcggggg ctccctttag
      181 ggttccgatt tagtgcttta cggcacctcg accccaaaaa acttgattag ggtgatggtt
      241 cacgtagtgg gccatcgccc tgatagacgg tttttcgccc tttgacgttg gagtccacgt
      301 tctttaatag tggactcttg ttccaaactg gaacaacact caaccctatc tcggtctatt
      361 cttttgattt ataagggatt ttgccgattt cggcctattg gttaaaaaat gagctgattt
      421 aacaaaaatt taacgcgaat tttaacaaaa tattaacgtt tacaatttca ggtggcactt
      481 ttcggggaaa tgtgcgcgga acccctattt gtttattttt ctaaatacat tcaaatatgt
      541 atccgctcat gaattaattc ttagaaaaac tcatcgagca tcaaatgaaa ctgcaattta
      601 ttcatatcag gattatcaat accatatttt tgaaaaagcc gtttctgtaa tgaaggagaa
      661 aactcaccga ggcagttcca taggatggca agatcctggt atcggtctgc gattccgact
      721 cgtccaacat caatacaacc tattaatttc ccctcgtcaa aaataaggtt atcaagtgag
      781 aaatcaccat gagtgacgac tgaatccggt gagaatggca aaagtttatg catttctttc
      841 cagacttgtt caacaggcca gccattacgc tcgtcatcaa aatcactcgc atcaaccaaa
      901 ccgttattca ttcgtgattg cgcctgagcg agacgaaata cgcgatcgct gttaaaagga
      961 caattacaaa caggaatcga atgcaaccgg cgcaggaaca ctgccagcgc atcaacaata
     1021 ttttcacctg aatcaggata ttcttctaat acctggaatg ctgttttccc ggggatcgca
```

(continued)

Figure S12. Complete and annotated DNA sequence for the empty parent vector pelB-MBP.

```
1081 gtggtgagta accatgcatc atcaggagta cggataaaat gcttgatggt cggaagaggc
1141 ataaattccg tcagccagtt tagtctgacc atctcatctg taacatcatt ggcaacgcta
1201 cctttgccat gtttcagaaa caactctggc gcatcgggct tcccatacaa tcgatagatt
1261 gtcgcacctg attgcccgac attatcgcga gcccatttat acccatataa atcagcatcc
1321 atgttggaat ttaatcgcgg cctagagcaa gacgtttccc gttgaatatg gctcataaca
1381 ccccttgtat tactgtttat gtaagcagac agttttattg ttcatgacca aaatccctta
1441 acgtgagttt tcgttccact gagcgtcaga ccccgtagaa aagatcaaag gatcttcttg
1501 agatcctttt tttctgcgcg taatctgctg cttgcaaaca aaaaaaccac cgctaccagc
1561 ggtggtttgt ttgccggatc aagagctacc aactcttttt ccgaaggtaa ctggcttcag
1621 cagagcgcag ataccaaata ctgtccttct agtgtagccg tagttaggcc accacttcaa
1681 gaactctgta gcaccgccta catacctcgc tctgctaatc ctgttaccag tggctgctgc
1741 cagtggcgat aagtcgtgtc ttaccgggtt ggactcaaga cgatagttac cggataaggc
1801 gcagcggtcg ggctgaacgg ggggttcgtg cacacagccc agcttggagc gaacgaccta
1861 caccgaactg agatacctac agcgtgagct atgagaaagc gccacgcttc ccgaagggag
1921 aaaggcggac aggtatccgg taagcggcag ggtcggaaca ggagagcgca cgagggagct
1981 tccaggggga aacgcctggt atctttatag tcctgtcggg tttcgccacc tctgacttga
2041 gcgtcgattt ttgtgatgct cgtcaggggg gcggagccta tggaaaaacg ccagcaacgc
2101 ggccttttta cggttcctgg ccttttgctg gccttttgct cacatgttct ttcctgcgtt
2161 atcccctgat tctgtggata accgtattac cgcctttgag tgagctgata ccgctcgccg
2221 cagccgaacg accgagcgca gcgagtcagt gagcgaggaa gcggaagagc gcctgatgcg
2281 gtattttctc cttacgcatc tgtgcggtat ttcacaccgc atatatggtg cactctcagt
2341 acaatctgct ctgatgccgc atagttaagc cagtatacac tccgctatcg ctacgtgact
2401 gggtcatggc tgcgccccga cacccgccaa cacccgctga cgcgccctga cgggcttgtc
2461 tgctcccggc atccgcttac agacaagctg tgaccgtctc cgggagctgc atgtgtcaga
2521 ggttttcacc gtcatcaccg aaacgcgcga ggcagctgcg gtaaagctca tcagcgtggt
2581 cgtgaagcga ttcacagatg tctgcctgtt catccgcgtc cagctcgttg agtttctcca
2641 gaagcgttaa tgtctggctt ctgataaagc gggccatgtt aagggcggtt ttttcctgtt
2701 tggtcactga tgcctccgtg taagggggat ttctgttcat gggggtaatg ataccgatga
2761 aacgagagag gatgctcacg atacgggtta ctgatgatga acatgcccgg ttactggaac
2821 gttgtgaggg taaacaactg gcggtatgga tgcggcggga ccagagaaaa atcactcagg
2881 gtcaatgcca gcgcttcgtt aatacagatg taggtgttcc acagggtagc cagcagcatc
2941 ctgcgatgca gatccggaac ataatggtgc agggcgctga cttccgcgtt tccagacttt
3001 acgaaacacg gaaaccgaag accattcatg ttgttgctca ggtcgcagac gttttgcagc
3061 agcagtcgct tcacgttcgc tcgcgtatcg gtgattcatt ctgctaacca gtaaggcaac
3121 cccgccagcc tagccgggtc ctcaacgaca ggagcacgat catgcgcacc cgtggggccg
3181 ccatgccggc gataatggcc tgcttctcgc cgaaacgttt ggtggcggga ccagtgacga
3241 aggcttgagc gagggcgtgc aagattccga ataccgcaag cgacaggccg atcatcgtcg
3301 cgctccagcg aaagcggtcc tcgccgaaaa tgacccagag cgctgccggc acctgtccta
3361 cgagttgcat gataaagaag acagtcataa gtgcggcgac gatagtcatg ccccgcgccc
3421 accggaagga gctgactggg ttgaaggctc tcaagggcat cggtcgagat cccggtgcct
3481 aatgagtgag ctaacttaca ttaattgcgt tgcgctcact gcccgctttc cagtcgggaa
3541 acctgtcgtg ccagctgcat taatgaatcg gccaacgcgc ggggagaggc ggtttgcgta
3601 ttgggcgcca gggtggtttt tcttttcacc agtgagacgg gcaacagctg attgcccttc
3661 accgcctggc cctgagagag ttgcagcaag cggtccacgc tggtttgccc cagcaggcga
3721 aaatcctgtt tgatggtggt taacggcggg atataacatg agctgtcttc ggtatcgtcg
3781 tatcccacta ccgagatatc cgcaccaacg cgcagcccgg actcggtaat ggcgcgcatt
3841 gcgcccagcg ccatctgatc gttggcaacc agcatcgcag tgggaacgat gccctcattc
3901 agcatttgca tggtttgttg aaaaccggac atggcactcc agtcgccttc ccgttccgct
3961 atcggctgaa tttgattgcg agtgagatat ttatgccagc cagccagacg cagacgcgcc
4021 gagacagaac ttaatgggcc cgctaacagc gcgatttgct ggtgacccaa tgcgaccaga
4081 tgctccacgc ccagtcgcgt accgtcttca tgggagaaaa taatactgtt gatgggtgtc
4141 tggtcagaga catcaagaaa taacgccgga acattagtgc aggcagcttc cacagcaatg
4201 gcatcctggt catccagcgg atagttaatg atcagcccac tgacgcgttg cgcgagaaga
4261 ttgtgcaccg ccgctttaca ggcttcgacg ccgcttcgtt ctaccatcga caccaccacg
4321 ctggcaccca gttgatcggc gcgagattta atcgccgcga caatttgcga cggcgcgtgc
4381 agggccagac tggaggtggc aacgccaatc agcaacgact gtttgcccgc cagttgttgt
4441 gccacgcggt tgggaatgta attcagctcc gccatcgccg cttccacttt ttcccgcgtt
4501 ttcgcagaaa cgtggctggc ctggttcacc acgcgggaaa cggtctgata agagacaccg
4561 gcatactctg cgacatcgta taacgttact ggtttcacat tcaccaccct gaattgactc
4621 tcttccgggc gctatcatgc cataccgcga aaggttttgc gccattcgat ggtgtccggg
4681 atctcgacgc tctcccttat gcgactcctg cattaggaag cagcccagta gtaggttgag
4741 gccgttgagc accgccgccg caaggaatgg tgcatgcaag gagatggcgc ccaacagtcc
4801 cccggccacg gggcctgcca ccatacccac gccgaaacaa gcgctcatga gcccgaagtg
4861 gcgagcccga tcttccccat cggtgatgtc ggcgatatag gcgccagcaa ccgcacctgt
4921 ggcgccggtg atgccggcca cgatgcgtcc ggcgtagagg atcgagatct cgatcccgcg
4981 aaattaatac gactcactat aggggaattg tgagcggata acaattcccc tctagaaata
5041 attttgttta actttaagaa ggagatatac atatgaaata cctgctgccg accgctgctg
5101 ctggtctgct gcttctcgct gcccagccgg cgatggccat ggatatcgga attaattcgg
5161 atccgaattc gagctcacat caccatcacc atcaccatca ccatcacccc atgaaaatcg
5221 aagaaggtaa actggtaatc tggattaacg gcgataaagg ctataacggt ctcgctgaag
5281 tcggtaagaa attcgagaaa gataccggaa ttaaagtcac cgttgagcat ccggataaac
5341 tggaagagaa attcccacag gttgcggcaa ctggcgatgg ccctgacatt atcttctggg
5401 cacacgaccg ctttggtggc tacgctcaat ctggcctgtt ggctgaaatc accccggaca
5461 aagcgttcca ggacaagctg tatccgttta cctgggatgc cgtacgttac aacggcaagc
5521 tgattgctta cccgatcgct gttgaagcgt tatcgctgat ttataacaaa gatctgctgc
5581 cgaacccgcc aaaaacctgg gaagagatcc cggcgctgga taaagaactg aaagcgaaag
5641 gtaagagcgc gctgatgttc aacctgcaag aaccgtactt cacctggccg ctgattgctg
5701 ctgacggggg ttatgcgttc aagtatgaaa acggcaagta cgacattaaa gacgtgggcg
5761 tggataacgc tggcgcgaaa gcgggtctga ccttcctggt tgacctgatt aaaaacaaac
5821 acatgaatgc agacaccgat tactccatcg cagaagctgc ctttaataaa ggcgaaacag
5881 cgatgaccat caacggcccg tgggcatggt ccaacatcga caccagcaaa gtgaattatg
5941 gtgtaacggt actgccgacc ttcaagggtc aaccatccaa accgttcgtt ggcgtgctga
6001 gcgcaggtat taacgccgcc agtccgaaca aagagctggc aaaagagttc ctcgaaaact
6061 atctgctgac tgatgaaggt ctggaagcgg ttaataaaga caaaccgctg ggtgccgtag
6121 cgctgaagtc ttacgaggaa gagttggcga aagatccacg tattgccgcc actatggaaa
6181 acgcccagaa aggtgaaatc atgccgaaca tcccgcagat gtccgctttc tggtatgccg
6241 tgcgtactgc ggtgatcaac gccgccagcg gtcgtcagac tgtcgatgaa gccctgaaag
6301 acgcgcagac tccgggtagc gaaaacctgt acttccaaag atctgtcgct cctcatgtag
6361 acaccgacac tgtgaacacc gccatcctgg gcgtttcaag gtccggcaaa ggcgagacaa
6421 tcgtagtgcc aatgatcgac aatctgagcc gcgcaaagaa ccagtctagc atggtcgtga
6481 acgaccccaa gggcgagctc tactctgcca gcaaggaaac acttgagaaa cgcggatacg
6541 acgtgcaggt gctgaacatc ctggatcccc tgcagggcat gagttacaac ccactccagc
6601 tcgtgatcga cgcctgggtg aacggcgacg accaggaggc tgcaaagagg gcaaacacac
6661 tgactttctc actgtacaac aacccaaatg ctggcgacaa cgctttcttt aacaccagcg
6721 cccagaatgc tatcaacgga atcatcctcg ctatgaggag gcagaagctt ctcgagcacc
6781 accaccacca ccactgagat ccggctgcta acaaagcccg aaaggaagct gagttggctg
6841 ctgccaccgc tgagcaataa ctagcataac cccttggggc ctctaaacgg gtcttgaggg
6901 gtttttttgct gaaaggagga actatatccg ga
//
```

Figure S12 (continued).

SUPPLEMENTARY TABLES

Table S1. All of the protein structures in the Protein Data Bank[1] from *Borrelia* spp. for proteins that contain membrane-targeting signal peptides in the full-length protein [a]

PDB ID	Protein	Species	Expression system	Signal peptide included in the expressed protein? [b]	Structure Method	Reference
Lipoproteins, outer surface/membrane						
4D53	BB0689	*B. burgdorferi*	*E. coli* BL21(DE3)	No	Crystal	[26]
6RJW; 6RJX	BB0323	*B. burgdorferi*	*E. coli* BL21(DE3)	No (lacks aa 1-25, 211-377)	Crystal; Crystal	[27]; [27]
4ALY	BBA64	*B. burgdorferi*	*E. coli* BL21(DE3)	No	Crystal	[28]
4BG5	BBA65	*B. burgdorferi*	*E. coli* BL21(DE3)	No (lacks aa 1-24)	Crystal	Unpublished
2YN7; 5IZT	BBA66	*B. burgdorferi*	*E. coli* BL21(DE3)	No; No (lacks aa 1-23)	Crystal; Crystal	[29]; Unpublished
6QO1; 6ROC	BBA69	*B. burgdorferi*	*E. coli* BL21(DE3)	No (lacks aa 1-75); No (lacks aa 1-75)	Crystal; Crystal	[30]; [30]
4AXZ; 4B2F	BBA73	*B. burgdorferi*	*E. coli* BL21(DE3)	No; No	Crystal; Crystal	[31]; [31]
6FXE; 6FZE	BBE31	*B. burgdorferi*	*E. coli* BL21	No (lacks aa 1-37); No (lacks aa 1-37)	Crystal; Crystal	Unpublished; Unpublished
4PZ5; 6N1L	BBK32 (Fbp)	*B. burgdorferi*	*E. coli* BL21(DE3)	No (only aa 175-187); No (only aa 206–348)	Crystal; Crystal	[32]; [33]
6FL0; 6FMH	BGA71	*B. bavariensis*	*E. coli* BL21(DE3)	No (lacks aa 1-61); No (lacks aa 1-61)	Crystal; Crystal	[34]; [34]
6FZZ	BSE31	*B. spielmanii*	*E. coli* BL21	No (lacks aa 1-92)	Crystal	Unpublished
1W33; 1W3Z; 4BL4; 5A2U	CspA (BBA68)	*B. burgdorferi*	*E. coli* or *E. coli* BL21(DE3)	No; No; No; C-terminal to GST-tag [c]; No (lacks aa 1-64)	Crystal; Crystal; Crystal; Crystal	[35]; [35]; [36]; Unpublished
4BG0; 4CBE; 6ATG	CspZ (BBH06)	*B. burgdorferi*	*E. coli* BL21(DE3)	No; No; No (lacks aa 1-24)	Crystal; Crystal; Crystal	[37]; [37]; Unpublished
2LQU; 4ONR; 2MTC	DbpA (BBA24)	*B. burgdorferi*	*E. coli* or *E. coli* BL21(DE3)	No; No; No	NMR; NMR; NMR	[38]; [39]; [40]
2MTD	DbpA (BGAPBR_A0025)	*B. garinii*	*E. coli* BL21(DE3)	No (lacks 1-54)	NMR	[40]
2MVG	DbpB (BBA25)	*B. burgdorferi*	*E. coli* BL21(DE3)	No	NMR	[41]
4BOB	ErpP (BBN38)	*B. burgdorferi*	*E. coli* BL21(DE3)	No	Crystal	[42]
1OSP; 1FJ1	OspA (BBA15)	*B. burgdorferi*	*E. coli*	No; No	Crystal; Crystal	[43]; [44]
1P4P; 1RJL	OspB (BBA16)	*B. burgdorferi*	*E. coli*	No (only aa 152-296); No (only aa 152-296)	Crystal; Crystal	[45]; [45]
1F1M; 1GGQ; 1G5Z	OspC (BBB19)	*B. burgdorferi*	*E. coli*	No; No; No	Crystal; Crystal; Crystal	[46]; [46]; [47]
4J38; 2M4F; 5NBQ	OspE (Erp47)	*B. burgdorferi*	*E. coli* or *E. coli* BL21(DE3)	C-terminal to GST-tag [c]; No; No	Crystal; NMR; Crystal	[48]; [48]; [49]
4N13	PstS (BB0215)	*B. burgdorferi*	*E. coli*	No	Crystal	[50]
5EQZ	RevA	*B. burgdorferi*	*E. coli* BL21(DE3)	No (lacks 1-22)	Crystal	Unpublished
1L8W	VlsE	*B. burgdorferi*	*E. coli*	No	Crystal	[51]
1YJG; 2GA0	Vsp1 (VspA)	*B. turicatae*	*E. coli* BL21(DE3)	No; No	Crystal; Crystal	[52]; [52]
Non-lipidated proteins, outer surface/membrane						
3OON	BB0167 (OmpA-like)	*B. burgdorferi*	*E. coli*	No (only aa 260-390)	Crystal	Unpublished
5VJ4	BTA121	*B. turicatae*	*E. coli*	No	Crystal	[53]
4BF3; 4BXM; 4BOD	ErpC	*B. burgdorferi*	*E. coli* BL21(DE3)	No; No; No	Crystal; Crystal; Crystal	[54]; [42]; [42]
Lipoproteins, inner membrane or periplasmic						
4KKS; 4KKT; 4KKU	BesA	*B. burgdorferi*	*E. coli*	No; No; No	Crystal; Crystal; Crystal	[55]; [55]; [55]
4GL8	OppA4	*B. burgdorferi*	*E. coli*	No (lacks aa 1-23)	Crystal	Unpublished
Non-lipidated proteins, inner membrane or periplasmic						
3TMG	BB0144	*B. burgdorferi*	*E. coli* BL21(DE3)	No (lacks aa 1-15)	Crystal	Unpublished
5DLE	BB0408 (FruA1)	*B. burgdorferi*	*E. coli* BL21(DE3)	No (only aa 167- 265)	Crystal	Unpublished
5BWJ	Hk1	*B. burgdorferi*	*E. coli*	No (only aa 23-249)	Crystal	[56]

[a] All structures as of August 6, 2019. Not included in the table are the many additional structures of OspA, which contain an engineered β-sheet domain or other mutations, all of which also lacked signal peptides. Abbreviations: aa, amino acid residue numbers; GST, glutathione S-transferase.

[b] For proteins that were truncated outside of the signal peptide, the amino acid residues that were expressed are listed in parentheses. For unpublished references, this information is based on the available sequence in the PDB file.

[c] For this protein, inclusion of the signal peptide is unclear. However, the signal peptide, if included, was placed C-terminal to a large purification tag, which prevents translocation of proteins to the membrane[57].

Table S2. Target virulence factors in this study: physical characteristics, roles in pathogenesis, and functions [a]

Protein name	Accession no.	Expected molecular weight, precursor/mature [b] [observed [c]] (kDa)	Membrane localization [d]	Hydrophobicity, precursor/mature [e]	Predicted no. of α-helical transmembrane domains, precursor/mature [f]; or predicted β-barrel formation (β)	Reported role in pathogenesis [g]	Function
BBA57	AAC66270.1	47.4/45.0	OM[58,59] lipoprotein[58,60,61]	29/27%	0-1/0	Transmission from tick to mouse[58] or human[62]; necessary for Lyme arthritis (joint inflammation) and carditis in mice[58]; weakens complement, neutrophil, interferon responses[62]; decreases transcription of antimicrobial peptides and interferon genes[62]; modulates *Bb* OspC, ErpP/B expression[62]; transcription increases from 23°C to 35°C[63]; protein is upregulated by temperature and by cultivation in mammalian host[60,64]; immunogen[61,65,66]	Unknown[62]
RevA (BBM27, BBP27) <u>rev</u>erse gene orientation[67, 68]; <u>A</u>, to distinguish from related proteins[69]	AAF07416.1	17.9/15.6 [13.6[70]; 16[69,71,72]]	OM[59,69,73] lipoprotein[59]	35/32%	1-2/0-1	Binds human plasma fibronectin and mouse laminin[74], but binds fibronectin less well than BBK32 does[75]; RevA mutant poorly infected mouse cardiac tissues, and had more arthritis and lower levels chemokine CCL2[76]; expressed in mammals, not ticks[74]; expression induced by temperature[69]; a component of multiprotein complexes in *Bb* OMVs only in Osp-deficient *Bb*[77]; immunogen[61,66,68,71,72]	Unknown, aside from possible adhesin activity[78]
P13 (BB0034)	AAC66426.1	19.1/16.8 [13-14[79-81]; 60[82]; 200-300[83]] [h]	OM[79,80]	48/46%	4-5/3-4	Expressed in infected mice[84]; dominant component of multiprotein complexes in *Bb* OMVs[77]; component of *Bb* OMVs[77,85] and *Bb* outer membrane[86] lipid rafts[86,87]	Unknown; voltage-independent diffusion porin[82,83] formed from homo-oligomers[83] with a single-channel conductance of 3.5[82,88] or 0.6[83] nS in 1 M KCl; has N-terminal pyroglutamylate post-translational modification[89]
HtrA (BB0104) <u>h</u>igh <u>t</u>emperature <u>r</u>equirement <u>A</u>[90]	AAC66500	52.1/48.9 [~46[90]; 48[91]; 52[92]]	OM[86,90,91]; soluble[90]; a smaller fraction[93] is in the periplasm[92]	40/39%	1-2/0-1	Degrades components (fibronectin; proteoglycans [including aggrecan[91]]; E-cadherin) of the host extracellular matrix, causing inflammatory responses[94]; necessary for mouse infection[95], possibly due to downstream effects on *Bb* proteins that are processed by HtrA; intermittent component of multiprotein complexes in *Bb* OMVs[77]; component of *Bb* OMVs[77,85,90] and *Bb* outer membrane[86] lipid rafts[86,87,96]; immunogen[90,91]	Temperature-dependent[95] serine protease[90]; processes several *Bb* OMPs[92,97] (BB0323[92], P66[97], Lmp1[98]) and non-OMPs[90,99] (BmpD[90]; CheX[90]; Hbb[99], FlaB[100]); possible role in periplasmic protein quality control[100]

Protein name	Accession no.	Expected molecular weight, precursor/mature[b] [observed[c]] (kDa)	Membrane localization[d]	Hydrophobicity, precursor/mature[e]	Predicted no. of α-helical transmembrane domains, precursor/mature[f]; or predicted β-barrel formation (β)	Reported role in pathogenesis[g]	Function
Lmp1 (BB0210) surface-located membrane protein 1[101]	YP_008686569.1	128.1/125.2 [~128[98,101], ~100[98], ~47[98]] [i]	OM[101,102]	29/28%	1/0	Central domain binds chondroitin-6-sulfate[103]; required for *Bb* persistence in immunocompetent mice[101]; may have a role in inflammation in the heart and joints[102]; contributes to complement-independent serum resistance[101]; ΔLmp1 impairs transmission from tick-to-mouse and from mouse-to-tick[104]; identified in in vivo selection in mice for *Bb* proteins that bind endothelial cells[105]; transcription increases from 23°C to 35°C[63]; immunogen[101,102]	Unknown, aside from adhesin activity[98]
BB0238	AAC66635.2	29.7/27.2 [27.9[70]; 28.1[70]; 27[106]]	IM[86,93,106]; or associated with outer leaflet of the IM[106]; a smaller fraction[93] is in the periplasm[93]	37/35%	1/0	Necessary for transmission from tick to mouse[93,106]; necessary for persistence in mouse[93]; infectivity is reduced in mice by point mutations in tetratricopeptide repeat (TPR) domain, which is predicted to mediate protein-protein interactions[106], although GST-tagged recombinant mature BB0238 does not interact with BB0323[107]; TPR deletion does not affect profile of major proteins[106]; component of *Bb* lipid rafts[86,87] at the inner membrane[86]; stability of the BB0323 virulence factor[93]; immunogen[61]	Unknown[107]
BB0323	AAC66700.1	44.1/42.0 [28.8[70]; ~30[72]; ~27[92]; 48.5[107]] [j]	OM[59,108] lipoprotein[59]; not surface-exposed[59,108]; a smaller fraction[93] is in the periplasm[92]	33/32%	0-1/0	Necessary for infection of ticks and mice and persistence in mice[108]; mutant impairs severity of arthritis in mice[108] and integrity of *Bb* outer membrane[108,109], cell fission and cell division[109]; component of *Bb* inner membrane[86] lipid rafts[86,87]; stability of the BB0238 virulence factor[93]; a component of multiprotein complexes in *Bb* OMVs only in Osp-deficient *Bb*[77]; immunogen[61,72]	Unknown[107]; N-terminal domain has structural homology to spectrin superfamily that function as linkers in bacterial cell division and the eukaryotic cytoskeleton[27]; C-terminal LysM domain is predicted to bind peptidoglycan[72] but is not required for OM stability or cell fission[92]
BB0405	AAC66795.1	22.3/20.3 [23[60]; ~22[110]]	OM[60,110,111]	41/39%	3-4/2-3; β [k]	Necessary for transmission from tick to mouse (also mouse to tick), and establishing infection in mice[111]; common component of multiprotein complexes in *Bb* OMVs[77]; transcription increases from 23°C to 35°C[63]; protein is upregulated by temperature and by cultivation in mammalian host[60]; not immunogenic in infected patients or mice[111]; immunogenic in infected non-human primates[112]; injecting mice with recombinant mature BB0405 led to a long-lasting, high titer (>1:450,000) antibody response that conferred protection against inoculation via ticks but not via syringe[111]	Unknown[112]; porin activity[110]

Protein name	Accession no.	Expected molecular weight, precursor/mature [b] [observed [c]] (kDa)	Membrane localization [d]	Hydrophobicity, precursor/mature [e]	Predicted no. of α-helical transmembrane domains, precursor/mature [f]; or predicted β-barrel formation (β)	Reported role in pathogenesis [g]	Function
BB0406	AAC66794.1	22.8/20.2 [l]	OM[110]; not surface-exposed[110]	40/38%	0-4/0-3; β [k]	Infected non-human primates have a delayed humoral immune response to BB0406[112]; a component of multiprotein complexes in *Bb* OMVs only in Osp-deficient *Bb*[77]	Unknown[112]; porin activity[110]
DipA (BB0418, Oms38) dicarboxylate-specific porin A[113]	AAC66790.1	38.8/36.5 [36[113]]	OM[110,113]	38/36%	0-5/0-4; β [k]	A role in uptake of host nutrients was indicated by inhibition of DipA channel conductance by amino acids (aspartate, glutamate) and by biosynthetic intermediates of the tricarboxylic acid cycle (malate, succinate, oxaloacetate and 2-oxoglutarate) and oxidative phosphorylation[113]; intermittent component of multiprotein complexes in *Bb* OMVs[77]	Voltage-independent porin with single channel conductance of 50 pS in 1 M KCl and specificity for a variety of dicarboxylates (1-3 carboxylic acid groups)[113]; may be an essential gene[113]
P66 (BB0603, Oms66 outer membrane spanning)	AAC66949.1	68.2/65.8 [70.0[70]; 66[72]; ~66[77]; 460[114] or ~400 and ~600[115]] [m]	OM[86,110,116,117]	34/33%	1-6/0-5; β [k]	Binds to the β_3-chain integrins[118,119] $\alpha_{IIb}\beta_3$[118] and $\alpha_v\beta_3$[118,119]; also binds β_1 integrins but at lower affinity[118,120]; necessary for infection of mouse (via injection or tick) but not of ticks (via immersion feeding)[121]; mechanism of immune clearance of the Δ*p66* mutant is unknown[121]; role in dissemination[122,123]: integrin binding mutants had reduced dissemination from the vasculature in vivo, and reduced endothelial transmigration in vitro[122]; Δ*p66* could not disseminate from skin inoculation site[123]; may be necessary for adhesion to heart and ear vasculature, as the mutant strain was absent in these tissues but was present in joints and bloodstream, as shown in retro-orbitally inoculated mice[124]; one of the most abundant proteins in *Bb*[77,125]; common component of multiprotein complexes in *Bb* OMVs[77]; component of *Bb* outer membrane lipid rafts[86]; does not contribute to OM stability, motility or chemotaxis[123]; expressed when *Bb* is in mammals, in culture, in engorged ticks[126] and in the mammalian-adapted environment of dialysis membrane chambers[127] but not in unfed ticks[126]; immunogen[61,66,72,128]; the natively folded P66, but not recombinantly expressed P66, raises serum bactericidal activity in rabbits and provides protection in mice against host-adapted OspABCD[-] *Bb*[129]; uninfected patients[130] and rabbits[131] also show serum antibody cross-reactivity with P66; no role in resistance to serum complement[122]; no role in phagocytosis by macrophages and skin dendritic cells[122]	Adhesin; in vivo role of the porin activity[123] and pore substrate(s)[114] are unknown; voltage-dependent non-selective[131] porin[115,131,132] with an unusually high single-channel conductance of 11 nS in 1 M KCl[114,131,133,134], which may be a consequence of a P66 octamer, where each subunit has a conductance of ~1.5 nS[114]; 1.9 nm diameter opening, 0.8 nm central constriction[114]; non-essential protein, as the mutant has no phenotype in vitro[77,119,133]

Table S2 (continued)

[a] Abbreviations: *Bb*, *Borrelia burgdorferi*; GST, glutathione S-transferase; IM, inner membrane; nS, nanosiemens; OM, outer membrane; OMP, outer membrane protein; OMV, outer membrane vesicle.

[b] Expected molecular weight of the precursor protein (prior to removal of the signal peptide) and of the mature protein (after removal of the signal peptide). Signal peptides were predicted by SignalP 4.1[135] or LipoP 1.0[136].

[c] Observed molecular weight of the protein that was isolated from *Bb* and as determined by the following methods. MALDI-TOF of membrane-associated proteins[70]. Blue native PAGE[83,114,115]. Liquid chromatography tandem mass spectrometry[91]. Western immunoblot[60,69,71,81,90-92,106,110,113]. Two-dimensional electrophoresis plus immunoblot with mammalian sera and MALDI-TOF[72]. Two-dimensional electrophoresis plus immunoblot[81]. Both by analytical size exclusion chromatography and by crosslinking plus SDS-PAGE[107]. SDS-PAGE[77,113]. For P13, Lmp1, BB0323 and BB0406, see also [h, i, j, l], respectively.

[d] References for membrane localization indicate results from physical localization studies in *Bb*, except for lipoprotein designations, which are based on predictions. Actual lipidation as well as the type of lipid have not been specifically demonstrated for these proteins.

[e] Percentage by weight of amino acid residues A, F, I, L, V, and W of the precursor protein (prior to removal of the signal peptide) and of the mature protein (after removal of the signal peptide). Signal peptides were predicted by SignalP 4.1[135] or LipoP 1.0[136].

[f] Shown are the number of α-helical transmembrane domains for the precursor protein (prior to removal of the signal peptide) and for the mature protein (after removal of the signal peptide) as predicted using the programs TMHMM[137], TMpred[138] and HMMTOP[139]. Note that these prediction programs usually identify the signal peptide as an α-helical transmembrane domain. Prediction of an α-helical domain is not evidence for formation of an α-helix in the outer membrane, because transmembrane α-helices in the outer membrane are amphipathic[140] or have unusual topologies[141].

[g] In this column, immunogenicity refers to antibody responses due to natural infection, not due to recombinantly expressed protein, except where specified.

[h] Although mature P13 is predicted to be 16.8 kDa, P13 appears as 13-14 kDa when purified by preparative SDS-PAGE from the wild type B31 strain or from strain B313 (an OspA$^-$ OspB$^-$ OspC$^-$ OspD$^-$ mutant) as determined by Western immunoblot[79,82], electrospray mass spectrometry (13.9 kDa)[80], and Coomassie stained SDS-PAGE[80]. The C-terminal 28 residues[80] of P13 are removed by the CtpA C-terminal protease[81]. For P13 purified by FPLC, an additional band at 60 kDa, comprised of a homo-oligomer, was observed in Western immunoblots[82].

[i] Lmp1 appears as ~110-140 kDa in cultured *Bb*[98,101] and in *Bb* isolated from ticks[98] as determined by Western. In cultured *Bb*, additional bands were visible by Western at ~100 kDa (containing middle and C-terminal Lmp1) and ~47 kDa (multiple bands containing N-terminal or middle regions; confirmed by LC-MS/MS)[98]. These truncated forms of Lmp1 are due at least in part to processing by HtrA protease[98].

[j] Although mature BB0323 is predicted to be 42.0 kDa, BB0323 was identified as a 28.8 kDa protein by MALDI-TOF of membrane-associated proteins[70]. Later it was shown that BB0323 is proteolyzed by HtrA to yield a 27 kDa N-terminal fragment[92] (residues 22–242) that forms a 48.5 kDa dimer, as determined by analytical size exclusion chromatography and by crosslinking[107]. The ~15 kDa C-terminal fragment is difficult to detect due to poor immunogenicity[92]. That the BB0323 N-terminal fragment interacts with BB0238 was shown using recombinant, mature BB0238 and BB0323 expressed in *E. coli*[93,107]. BB0323 is also proteolyzed by the CtpA C-terminal protease[81].

[k] β-barrel structures have not been demonstrated structurally for these proteins. The β-barrel prediction is based on demonstrated porin activity for BB0405[110], BB0406[110], DipA[113] and P66[115]. A DipA β-barrel was

proposed based on predicted β-strands[113]. P66 is predicted to have 22 or 24 transmembrane β-strands[115].

[l] To our knowledge, there are no reports of the observed size of BB0406 from *Bb*. The mature form of BB0406 recombinantly expressed in the *E. coli* cytoplasm migrated at ~22 kDa as determined by Western[112].

[m] P66 is processed by HtrA[97]. In vivo, HtrA overexpression reduces P66 levels[97,99]. In vitro, HtrA degrades P66[97].

Table S3. Plasmid vectors generated in this study

Plasmid name	Notes
Empty parent vectors	
pRSET-TEV-12His	Adds C-terminal TEV protease cleavage site (ENLYFQG) + His_{12}. *Bse*RI cloning sites. $Ampicillin^R$.
pelB-MBP	Adds N-terminal PelB signal sequence + His_{10} + maltose binding protein + TEV protease cleavage site. *Bse*RI cloning sites. Available from the DNASU[142] Plasmid Repository, accession number EvNO00813783. $Kanamycin^R$.
Subclones	
pUC57-BBclone1	PCR template for expression optimized genes encoding BB0405, BB0406, P66, BBA57, BbHtrA, RevA, BB0238. $Ampicillin^R$. ColE1 origin of replication. 9655 bp
pCC1-4k-BBclone2	PCR template for expression optimized genes encoding BB0323, P13, DipA, Lmp1. $Chloramphenicol^R$. RK2 origin of replication. 10324 bp
pBBA57-TEV-His12	Expression clone. $Ampicillin^R$.
pRevA-TEV-His12	Expression clone. $Ampicillin^R$.
pP13-TEV-His12	Expression clone. $Ampicillin^R$.
pHtrA-TEV-His12	Expression clone. $Ampicillin^R$.
pLmp1-TEV-His12	Expression clone. $Ampicillin^R$.
pBB0238-TEV-His12	Expression clone. $Ampicillin^R$.
pBB0323-TEV-His12	Expression clone. $Ampicillin^R$.
pBB0405-TEV-His12	Expression clone. $Ampicillin^R$.
pBB0406-TEV-His12	Expression clone. $Ampicillin^R$.
pDipA-TEV-His12	Expression clone. $Ampicillin^R$.
pP66-TEV-His12	Expression clone. $Ampicillin^R$.
pelB-MBP-BB0238-ss	Expression clone. $Kanamycin^R$.
pelB-MBP-P66-ss	Expression clone. $Kanamycin^R$.
pelB-MBP-RevA-ss	Expression clone. $Kanamycin^R$.
pelB-MBP-P13-ss	Expression clone. $Kanamycin^R$.
pelB-MBP-HtrA-ss	Expression clone. $Kanamycin^R$.
pelB-MBP-BB0323-ss	Expression clone. $Kanamycin^R$.
pelB-MBP-DipA-ss	Expression clone. $Kanamycin^R$.

SUPPLEMENTARY REFERENCES

1. Rose, P.W., Prlić, A., Altunkaya, A., Bi, C., Bradley, A.R., Christie, C.H., Costanzo, L.D., Duarte, J.M., Dutta, S., Feng, Z., Green, R.K., Goodsell, D.S., Hudson, B., Kalro, T., Lowe, R., Peisach, E., Randle, C., Rose, A.S., Shao, C., Tao, Y.P., Valasatava, Y., Voigt, M., Westbrook, J.D., Woo, J., Yang, H., Young, J.Y., Zardecki, C., Berman, H.M. & Burley, S.K. The RCSB protein data bank: integrative view of protein, gene and 3D structural information. *Nucleic Acids Res.* **45**, D271-D281 (2017).
2. Hofmann, K.S., W. TMBASE - A database of membrane spanning protein segments. *Biol. Chem.* **374**, 166 (1993).
3. Tusnády, G.E. & Simon, I. Principles governing amino acid composition of integral membrane proteins: application to topology prediction. *J. Mol. Biol.* **283**, 489-506 (1998).
4. Krogh, A., Larsson, B., von Heijne, G. & Sonnhammer, E.L. Predicting transmembrane protein topology with a hidden Markov model: application to complete genomes. *J. Mol. Biol.* **305**, 567-580 (2001).
5. Juncker, A.S., Willenbrock, H., Von Heijne, G., Brunak, S., Nielsen, H. & Krogh, A. Prediction of lipoprotein signal peptides in Gram-negative bacteria. *Protein Sci.* **12**, 1652-1662 (2003).
6. Bagos, P.G., Liakopoulos, T.D., Spyropoulos, I.C. & Hamodrakas, S.J. PRED-TMBB: a web server for predicting the topology of β-barrel outer membrane proteins. *Nucleic Acids Res.* **32**, W400-404 (2004).
7. Berven, F.S., Flikka, K., Jensen, H.B. & Eidhammer, I. BOMP: a program to predict integral β-barrel outer membrane proteins encoded within genomes of Gram-negative bacteria. *Nucleic Acids Res.* **32**, W394-W399 (2004).
8. Natt, N.K., Kaur, H. & Raghava, G.P. Prediction of transmembrane regions of beta-barrel proteins using ANN- and SVM-based methods. *Proteins* **56**, 11-18 (2004).
9. Nugent, T. & Jones, D.T. Transmembrane protein topology prediction using support vector machines. *BMC Bioinformatics* **10**, 159-159 (2009).
10. Leman, J.K., Mueller, R., Karakas, M., Woetzel, N. & Meiler, J. Simultaneous prediction of protein secondary structure and transmembrane spans. *Proteins* **81**, 1127-1140 (2013).
11. Hayat, S., Peters, C., Shu, N., Tsirigos, K.D. & Elofsson, A. Inclusion of dyad-repeat pattern improves topology prediction of transmembrane β-barrel proteins. *Bioinformatics* **32**, 1571-1573 (2016).
12. Hansen, D.T., Robida, M.D., Craciunescu, F.M., Loskutov, A.V., Dörner, K., Rodenberry, J.C., Wang, X., Olson, T.L., Patel, H., Fromme, P. & Sykes, K.F. Polyclonal antibody production for membrane proteins *via* genetic immunization. *Sci. Rep.* **6**, 21925 (2016).
13. Martin-Garcia, J.M., Hansen, D.T., Zook, J., Loskutov, A.V., Robida, M.D., Craciunescu, F.M., Sykes, K.F., Wachter, R.M., Fromme, P. & Allen, J.P. Purification and biophysical characterization of the CapA membrane protein FTT0807 from *Francisella tularensis*. *Biochemistry* **53**, 1958-1970 (2014).
14. Lobley, A., Whitmore, L. & Wallace, B.A. DICHROWEB: an interactive website for the analysis of protein secondary structure from circular dichroism spectra. *Bioinformatics* **18**, 211-212 (2002).
15. Whitmore, L. & Wallace, B.A. DICHROWEB, an online server for protein secondary structure analyses from circular dichroism spectroscopic data. *Nucleic Acids Res.* **32**, W668-673 (2004).
16. Whitmore, L. & Wallace, B.A. Protein secondary structure analyses from circular dichroism spectroscopy: methods and reference databases. *Biopolymers* **89**, 392-400 (2008).

17. Sreerama, N. & Woody, R.W. Estimation of protein secondary structure from circular dichroism spectra: comparison of CONTIN, SELCON, and CDSSTR methods with an expanded reference set. *Anal. Biochem.* **287**, 252-260 (2000).

18. Manavalan, P. & Johnson, W.C., Jr. Variable selection method improves the prediction of protein secondary structure from circular dichroism spectra. *Anal. Biochem.* **167**, 76-85 (1987).

19. Compton, L.A. & Johnson, W.C., Jr. Analysis of protein circular dichroism spectra for secondary structure using a simple matrix multiplication. *Anal. Biochem.* **155**, 155-167 (1986).

20. van Stokkum, I.H., Spoelder, H.J., Bloemendal, M., van Grondelle, R. & Groen, F.C. Estimation of protein secondary structure and error analysis from circular dichroism spectra. *Anal. Biochem.* **191**, 110-118 (1990).

21. Provencher, S.W. & Glockner, J. Estimation of globular protein secondary structure from circular dichroism. *Biochemistry* **20**, 33-37 (1981).

22. Sreerama, N., Venyaminov, S.Y. & Woody, R.W. Estimation of the number of alpha-helical and beta-strand segments in proteins using circular dichroism spectroscopy. *Protein Sci.* **8**, 370-380 (1999).

23. Sreerama, N. & Woody, R.W. A self-consistent method for the analysis of protein secondary structure from circular dichroism. *Anal. Biochem.* **209**, 32-44 (1993).

24. Abdul-Gader, A., Miles, A.J. & Wallace, B.A. A reference dataset for the analyses of membrane protein secondary structures and transmembrane residues using circular dichroism spectroscopy. *Bioinformatics* **27**, 1630-1636 (2011).

25. Sreerama, N., Venyaminov, S.Y. & Woody, R.W. Estimation of protein secondary structure from circular dichroism spectra: inclusion of denatured proteins with native proteins in the analysis. *Anal. Biochem.* **287**, 243-251 (2000).

26. Brangulis, K., Jaudzems, K., Petrovskis, I., Akopjana, I., Kazaks, A. & Tars, K. Structural and functional analysis of BB0689 from *Borrelia burgdorferi*, a member of the bacterial CAP superfamily. *J. Struct. Biol.* **192**, 320-330 (2015).

27. Brangulis, K., Akopjana, I., Kazaks, A. & Tars, K. Crystal structure of the N-terminal domain of the major virulence factor BB0323 from the Lyme disease agent *Borrelia burgdorferi*. *Acta Crystallogr D Struct Biol* **75**, 825-830 (2019).

28. Brangulis, K., Tars, K., Petrovskis, I., Kazaks, A., Ranka, R. & Baumanis, V. Structure of an outer surface lipoprotein BBA64 from the Lyme disease agent *Borrelia burgdorferi* which is critical to ensure infection after a tick bite. *Acta Crystallogr. D Biol. Crystallogr.* **69**, 1099-1107 (2013).

29. Brangulis, K., Petrovskis, I., Kazaks, A., Tars, K. & Ranka, R. Crystal structure of the infectious phenotype-associated outer surface protein BBA66 from the Lyme disease agent *Borrelia burgdorferi*. *Ticks Tick Borne Dis.* **5**, 63-68 (2014).

30. Brangulis, K., Akopjana, I., Petrovskis, I., Kazaks, A. & Tars, K. Crystal structure of *Borrelia burgdorferi* outer surface protein BBA69 in comparison to the paralogous protein CspA. *Ticks Tick Borne Dis.* **10**, 1135-1141 (2019).

31. Brangulis, K., Petrovskis, I., Kazaks, A., Baumanis, V. & Tars, K. Structural characterization of the *Borrelia burgdorferi* outer surface protein BBA73 implicates dimerization as a functional mechanism. *Biochem. Biophys. Res. Commun.* **434**, 848-853 (2013).

32. Harris, G., Ma, W., Maurer, L.M., Potts, J.R. & Mosher, D.F. *Borrelia burgdorferi* protein BBK32 binds to soluble fibronectin via the N-terminal 70-kDa region, causing fibronectin to undergo conformational extension. *J. Biol. Chem.* **289**, 22490-22499 (2014).

33. Xie, J., Zhi, H., Garrigues, R.J., Keightley, A., Garcia, B.L. & Skare, J.T. Structural determination of the complement inhibitory domain of *Borrelia burgdorferi* BBK32 provides insight into classical pathway complement evasion by Lyme disease spirochetes. *PLoS Pathog.* **15**, e1007659 (2019).

34. Brangulis, K., Akopjana, I., Petrovskis, I., Kazaks, A., Kraiczy, P. & Tars, K. Crystal structure of the membrane attack complex assembly inhibitor BGA71 from the Lyme disease agent *Borrelia bavariensis*. *Sci. Rep.* **8**, 11286 (2018).

35. Cordes, F.S., Roversi, P., Kraiczy, P., Simon, M.M., Brade, V., Jahraus, O., Wallis, R., Skerka, C., Zipfel, P.F., Wallich, R. & Lea, S.M. A novel fold for the factor H-binding protein BbCRASP-1 of *Borrelia burgdorferi*. *Nat. Struct. Mol. Biol.* **12**, 276-277 (2005).

36. Caesar, J.J., Wallich, R., Kraiczy, P., Zipfel, P.F. & Lea, S.M. Further structural insights into the binding of complement factor H by complement regulator-acquiring surface protein 1 (CspA) of *Borrelia burgdorferi*. *Acta Crystallogr. Sect. F Struct. Biol. Cryst. Commun.* **69**, 629-633 (2013).

37. Brangulis, K., Petrovskis, I., Kazaks, A., Bogans, J., Otikovs, M., Jaudzems, K., Ranka, R. & Tars, K. Structural characterization of CspZ, a complement regulator factor H and FHL-1 binding protein from *Borrelia burgdorferi*. *FEBS J* **281**, 2613-2622 (2014).

38. Wang, X. Solution structure of decorin-binding protein A from *Borrelia burgdorferi*. *Biochemistry* **51**, 8353-8362 (2012).

39. Fortune, D.E., Lin, Y.P., Deka, R.K., Groshong, A.M., Moore, B.P., Hagman, K.E., Leong, J.M., Tomchick, D.R. & Blevins, J.S. Identification of lysine residues in the *Borrelia burgdorferi* DbpA adhesin required for murine infection. *Infect. Immun.* **82**, 3186-3198 (2014).

40. Morgan, A.M. & Wang, X. Structural mechanisms underlying sequence-dependent variations in GAG affinities of decorin binding protein A, a *Borrelia burgdorferi* adhesin. *Biochem. J.* **467**, 439-451 (2015).

41. Feng, W. & Wang, X. Structure of decorin binding protein B from *Borrelia burgdorferi* and its interactions with glycosaminoglycans. *Biochim. Biophys. Acta* **1854**, 1823-1832 (2015).

42. Brangulis, K., Petrovskis, I., Kazaks, A., Akopjana, I. & Tars, K. Crystal structures of the Erp protein family members ErpP and ErpC from *Borrelia burgdorferi* reveal the reason for different affinities for complement regulator factor H. *Biochim. Biophys. Acta* **1854**, 349-355 (2015).

43. Li, H., Dunn, J.J., Luft, B.J. & Lawson, C.L. Crystal structure of Lyme disease antigen outer surface protein A complexed with an Fab. *Proc. Natl. Acad. Sci. U. S. A.* **94**, 3584-3589 (1997).

44. Ding, W., Huang, X., Yang, X., Dunn, J.J., Luft, B.J., Koide, S. & Lawson, C.L. Structural identification of a key protective B-cell epitope in Lyme disease antigen OspA. *J. Mol. Biol.* **302**, 1153-1164 (2000).

45. Becker, M., Bunikis, J., Lade, B.D., Dunn, J.J., Barbour, A.G. & Lawson, C.L. Structural investigation of *Borrelia burgdorferi* OspB, a bactericidal Fab target. *J. Biol. Chem.* **280**, 17363-17370 (2005).

46. Kumaran, D., Eswaramoorthy, S., Luft, B.J., Koide, S., Dunn, J.J., Lawson, C.L. & Swaminathan, S. Crystal structure of outer surface protein C (OspC) from the Lyme disease spirochete, *Borrelia burgdorferi*. *EMBO J.* **20**, 971-978 (2001).

47. Eicken, C., Sharma, V., Klabunde, T., Owens, R.T., Pikas, D.S., Höök, M. & Sacchettini, J.C. Crystal structure of Lyme disease antigen outer surface protein C from *Borrelia burgdorferi*. *J. Biol. Chem.* **276**, 10010-10015 (2001).

48. Bhattacharjee, A., Oeemig, J.S., Kolodziejczyk, R., Meri, T., Kajander, T., Lehtinen, M.J., Iwaï, H., Jokiranta, T.S. & Goldman, A. Structural basis for complement evasion by Lyme disease pathogen *Borrelia burgdorferi*. *J. Biol. Chem.* **288**, 18685-18695 (2013).

49. Kolodziejczyk, R., Mikula, K.M., Kotila, T., Postis, V.L.G., Jokiranta, T.S., Goldman, A. & Meri, T. Crystal structure of a tripartite complex between C3dg, C-terminal domains of factor H and OspE of *Borrelia burgdorferi*. *PLoS One* **12**, e0188127 (2017).

50. Brautigam, C.A., Ouyang, Z., Deka, R.K. & Norgard, M.V. Sequence, biophysical, and structural analyses of the PstS lipoprotein (BB0215) from *Borrelia burgdorferi* reveal a likely binding component of an ABC-type phosphate transporter. *Protein Sci.* **23**, 200-212 (2014).

51. Eicken, C., Sharma, V., Klabunde, T., Lawrenz, M.B., Hardham, J.M., Norris, S.J. & Sacchettini, J.C. Crystal structure of Lyme disease variable surface antigen VlsE of *Borrelia burgdorferi*. *J. Biol. Chem.* **277**, 21691-21696 (2002).

52. Lawson, C.L., Yung, B.H., Barbour, A.G. & Zückert, W.R. Crystal structure of neurotropism-associated variable surface protein 1 (Vsp1) of *Borrelia turicatae*. *J. Bacteriol.* **188**, 4522-4530 (2006).

53. Luo, Z., Kelleher, A.J., Darwiche, R., Hudspeth, E.M., Shittu, O.K., Krishnavajhala, A., Schneiter, R., Lopez, J.E. & Asojo, O.A. Crystal Structure of *Borrelia turicatae* protein, BTA121, a differentially regulated gene in the tick-mammalian transmission cycle of relapsing fever spirochetes. *Sci. Rep.* **7**, 15310 (2017).

54. Caesar, J.J., Johnson, S., Kraiczy, P. & Lea, S.M. ErpC, a member of the complement regulator-acquiring family of surface proteins from *Borrelia burgdorferi*, possesses an architecture previously unseen in this protein family. *Acta Crystallogr. Sect. F Struct. Biol. Cryst. Commun.* **69**, 624-628 (2013).

55. Greene, N.P., Hinchliffe, P., Crow, A., Ababou, A., Hughes, C. & Koronakis, V. Structure of an atypical periplasmic adaptor from a multidrug efflux pump of the spirochete *Borrelia burgdorferi*. *FEBS Lett.* **587**, 2984-2988 (2013).

56. Bauer, W.J., Luthra, A., Zhu, G., Radolf, J.D., Malkowski, M.G. & Caimano, M.J. Structural characterization and modeling of the *Borrelia burgdorferi* hybrid histidine kinase Hk1 periplasmic sensor: A system for sensing small molecules associated with tick feeding. *J. Struct. Biol.* **192**, 48-58 (2015).

57. Kang, H.J., Lee, C. & Drew, D. Breaking the barriers in membrane protein crystallography. *Int. J. Biochem. Cell Biol.* **45**, 636-644 (2013).

58. Yang, X., Qin, J., Promnares, K., Kariu, T., Anderson, J.F. & Pal, U. Novel microbial virulence factor triggers murine Lyme arthritis. *J. Infect. Dis.* **207**, 907-918 (2013).

59. Dowdell, A.S., Murphy, M.D., Azodi, C., Swanson, S.K., Florens, L., Chen, S. & Zückert, W.R. Comprehensive spatial analysis of the *Borrelia burgdorferi* lipoproteome reveals a compartmentalization bias toward the bacterial surface. *J. Bacteriol.* **199**, e00658-00616 (2017).

60. Brooks, C.S., Vuppala, S.R., Jett, A.M. & Akins, D.R. Identification of *Borrelia burgdorferi* outer surface proteins. *Infect. Immun.* **74**, 296-304 (2006).

61. Barbour, A.G., Jasinskas, A., Kayala, M.A., Davies, D.H., Steere, A.C., Baldi, P. & Felgner, P.L. A genome-wide proteome array reveals a limited set of immunogens in natural infections of humans and white-footed mice with *Borrelia burgdorferi*. *Infect. Immun.* **76**, 3374-3389 (2008).

62. Bernard, Q., Smith, A.A., Yang, X., Koci, J., Foor, S.D., Cramer, S.D., Zhuang, X., Dwyer, J.E., Lin, Y.P., Mongodin, E.F., Marques, A., Leong, J.M., Anguita, J. & Pal, U. Plasticity in early immune evasion strategies of a bacterial pathogen. *Proc Natl Acad Sci U S A* (2018).

63. Ojaimi, C., Brooks, C., Casjens, S., Rosa, P., Elias, A., Barbour, A., Jasinskas, A., Benach, J., Katona, L., Radolf, J., Caimano, M., Skare, J., Swingle, K., Akins, D. & Schwartz, I. Profiling of temperature-induced changes in *Borrelia burgdorferi* gene expression by using whole genome arrays. *Infect. Immun.* **71**, 1689-1705 (2003).

64. Caimano, M.J., Iyer, R., Eggers, C.H., Gonzalez, C., Morton, E.A., Gilbert, M.A., Schwartz, I. & Radolf, J.D. Analysis of the RpoS regulon in *Borrelia burgdorferi* in response to mammalian host signals provides insight into RpoS function during the enzootic cycle. *Mol. Microbiol.* **65**, 1193-1217 (2007).

65. Baum, E., Grosenbaugh, D.A. & Barbour, A.G. Diversity of antibody responses to *Borrelia burgdorferi* in experimentally infected beagle dogs. *Clin. Vaccine Immunol.* **21**, 838-846 (2014).

66. Tokarz, R., Mishra, N., Tagliafierro, T., Sameroff, S., Caciula, A., Chauhan, L., Patel, J., Sullivan, E., Gucwa, A., Fallon, B., Golightly, M., Molins, C., Schriefer, M., Marques, A., Briese, T. & Lipkin, W.I. A multiplex serologic platform for diagnosis of tick-borne diseases. *Sci. Rep.* **8**, 3158 (2018).

67. Porcella, S.F., Popova, T.G., Akins, D.R., Li, M., Radolf, J.D. & Norgard, M.V. *Borrelia burgdorferi* supercoiled plasmids encode multicopy tandem open reading frames and a lipoprotein gene family. *J. Bacteriol.* **178**, 3293-3307 (1996).

68. Gilmore, R.D., Jr. & Mbow, M.L. A monoclonal antibody generated by antigen inoculation via tick bite is reactive to the *Borrelia burgdorferi* Rev protein, a member of the 2.9 gene family locus. *Infect. Immun.* **66**, 980-986 (1998).

69. Carroll, J.A., El-Hage, N., Miller, J.C., Babb, K. & Stevenson, B. *Borrelia burgdorferi* RevA antigen is a surface-exposed outer membrane protein whose expression is regulated in response to environmental temperature and pH. *Infect. Immun.* **69**, 5286-5293 (2001).

70. Nowalk, A.J., Nolder, C., Clifton, D.R. & Carroll, J.A. Comparative proteome analysis of subcellular fractions from *Borrelia burgdorferi* by NEPHGE and IPG. *Proteomics* **6**, 2121-2134 (2006).

71. Mbow, M.L., Gilmore, R.D., Jr., Stevenson, B., Golde, W.T., Piesman, J. & Johnson, B.J. *Borrelia burgdorferi*-specific monoclonal antibodies derived from mice primed with Lyme disease spirochete-infected *Ixodes scapularis* ticks. *Hybrid Hybridomics* **21**, 179-182 (2002).

72. Nowalk, A.J., Gilmore, R.D., Jr. & Carroll, J.A. Serologic proteome analysis of *Borrelia burgdorferi* membrane-associated proteins. *Infect. Immun.* **74**, 3864-3873 (2006).

73. Skare, J.T., Foley, D.M., Hernandez, S.R., Moore, D.C., Blanco, D.R., Miller, J.N. & Lovett, M.A. Cloning and molecular characterization of plasmid-encoded antigens of *Borrelia burgdorferi*. *Infect. Immun.* **67**, 4407-4417 (1999).

74. Brissette, C.A., Bykowski, T., Cooley, A.E., Bowman, A. & Stevenson, B. *Borrelia burgdorferi* RevA antigen binds host fibronectin. *Infect. Immun.* **77**, 2802-2812 (2009).

75. Moriarty, T.J., Shi, M., Lin, Y.P., Ebady, R., Zhou, H., Odisho, T., Hardy, P.O., Salman-Dilgimen, A., Wu, J., Weening, E.H., Skare, J.T., Kubes, P., Leong, J. & Chaconas, G. Vascular binding of a pathogen under shear force through mechanistically distinct sequential interactions with host macromolecules. *Mol. Microbiol.* **86**, 1116-1131 (2012).

76. Byram, R., Gaultney, R.A., Floden, A.M., Hellekson, C., Stone, B.L., Bowman, A., Stevenson, B., Johnson, B.J. & Brissette, C.A. *Borrelia burgdorferi* RevA significantly affects pathogenicity and host response in the mouse model of Lyme disease. *Infect. Immun.* **83**, 3675-3683 (2015).

77. Yang, X., Promnares, K., Qin, J., He, M., Shroder, D.Y., Kariu, T., Wang, Y. & Pal, U. Characterization of multiprotein complexes of the *Borrelia burgdorferi* outer membrane vesicles. *J. Proteome Res.* **10**, 4556-4566 (2011).

78. Caine, J.A. & Coburn, J. Multifunctional and redundant roles of *Borrelia burgdorferi* outer surface proteins in tissue adhesion, colonization, and complement evasion. *Front. Immunol.* **7**, 442 (2016).

79. Sadziene, A., Thomas, D.D. & Barbour, A.G. *Borrelia burgdorferi* mutant lacking Osp: biological and immunological characterization. *Infect. Immun.* **63**, 1573-1580 (1995).

80. Noppa, L., Östberg, Y., Lavrinovicha, M. & Bergström, S. P13, an integral membrane protein of *Borrelia burgdorferi*, is C-terminally processed and contains surface-exposed domains. *Infect. Immun.* **69**, 3323-3334 (2001).

81. Östberg, Y., Carroll, J.A., Pinne, M., Krum, J.G., Rosa, P. & Bergström, S. Pleiotropic effects of inactivating a carboxyl-terminal protease, CtpA, in *Borrelia burgdorferi*. *J. Bacteriol.* **186**, 2074-2084 (2004).

82. Östberg, Y., Pinne, M., Benz, R., Rosa, P. & Bergström, S. Elimination of channel-forming activity by insertional inactivation of the *p13* gene in *Borrelia burgdorferi*. *J. Bacteriol.* **184**, 6811-6819 (2002).

83. Bárcena-Uribarri, I., Thein, M., Barbot, M., Sans-Serramitjana, E., Bonde, M., Mentele, R., Lottspeich, F., Bergström, S. & Benz, R. Study of the protein complex, pore diameter, and pore-forming activity of the *Borrelia burgdorferi* P13 porin. *J. Biol. Chem.* **289**, 18614-18624 (2014).

84. Pinne, M., Östberg, Y., Comstedt, P. & Bergström, S. Molecular analysis of the channel-forming protein P13 and its paralogue family 48 from different Lyme disease *Borrelia* species. *Microbiology* **150**, 549-559 (2004).

85. Toledo, A., Coleman, J.L., Kuhlow, C.J., Crowley, J.T. & Benach, J.L. The enolase of *Borrelia burgdorferi* is a plasminogen receptor released in outer membrane vesicles. *Infect. Immun.* **80**, 359-368 (2012).

86. Toledo, A., Huang, Z., Coleman, J.L., London, E. & Benach, J.L. Lipid rafts can form in the inner and outer membranes of *Borrelia burgdorferi* and have different properties and associated proteins. *Mol. Microbiol.* **108**, 63-76 (2018).

87. Toledo, A., Pérez, A., Coleman, J.L. & Benach, J.L. The lipid raft proteome of *Borrelia burgdorferi*. *Proteomics* **15**, 3662-3675 (2015).

88. Pinne, M., Denker, K., Nilsson, E., Benz, R. & Bergström, S. The BBA01 protein, a member of paralog family 48 from *Borrelia burgdorferi*, is potentially interchangeable with the channel-forming protein P13. *J. Bacteriol.* **188**, 4207-4217 (2006).

89. Nilsson, C.L., Cooper, H.J., Håkansson, K., Marshall, A.G., Östberg, Y., Lavrinovicha, M. & Bergström, S. Characterization of the P13 membrane protein of *Borrelia burgdorferi* by mass spectrometry. *J. Am. Soc. Mass Spectrom.* **13**, 295-299 (2002).

90. Coleman, J.L., Crowley, J.T., Toledo, A.M. & Benach, J.L. The HtrA protease of *Borrelia burgdorferi* degrades outer membrane protein BmpD and chemotaxis phosphatase CheX. *Mol. Microbiol.* **88**, 619-633 (2013).

91. Russell, T.M. & Johnson, B.J. Lyme disease spirochaetes possess an aggrecan-binding protease with aggrecanase activity. *Mol. Microbiol.* **90**, 228-240 (2013).

92. Kariu, T., Yang, X., Marks, C.B., Zhang, X. & Pal, U. Proteolysis of BB0323 results in two polypeptides that impact physiologic and infectious phenotypes in *Borrelia burgdorferi*. *Mol. Microbiol.* **88**, 510-522 (2013).

93. Kariu, T., Sharma, K., Singh, P., Smith, A.A., Backstedt, B., Buyuktanir, O. & Pal, U. BB0323 and novel virulence determinant BB0238: *Borrelia burgdorferi* proteins that interact with and stabilize each other and are critical for infectivity. *J. Infect. Dis.* **211**, 462-471 (2015).

94. Russell, T.M., Delorey, M.J. & Johnson, B.J. *Borrelia burgdorferi* BbHtrA degrades host ECM proteins and stimulates release of inflammatory cytokines *in vitro*. *Mol. Microbiol.* **90**, 241-251 (2013).

95. Ye, M., Sharma, K., Thakur, M., Smith, A.A., Buyuktanir, O., Xiang, X., Yang, X., Promnares, K., Lou, Y., Yang, X.F. & Pal, U. HtrA, a temperature- and stationary phase-activated protease involved in maturation of a key microbial virulence determinant, facilitates *Borrelia burgdorferi* infection in mammalian hosts. *Infect. Immun.* **84**, 2372-2381 (2016).

96. Toledo, A., Crowley, J.T., Coleman, J.L., LaRocca, T.J., Chiantia, S., London, E. & Benach, J.L. Selective association of outer surface lipoproteins with the lipid rafts of *Borrelia burgdorferi*. *MBio* **5**, e00899-00814 (2014).

97. Coleman, J.L., Toledo, A. & Benach, J.L. *Borrelia burgdorferi* HtrA: evidence for twofold proteolysis of outer membrane protein p66. *Mol. Microbiol.* **99**, 135-150 (2016).

98. Zhuang, X., Yang, X., Altieri, A.S., Nelson, D.C. & Pal, U. *Borrelia burgdorferi* surface-located Lmp1 protein processed into region-specific polypeptides that are critical for microbial persistence. *Cell. Microbiol.* **20**, e12855 (2018).

99. Coleman, J.L., Toledo, A. & Benach, J.L. HtrA of *Borrelia burgdorferi* leads to decreased swarm motility and decreased production of pyruvate. *MBio* **9**, e01136-01118 (2018).

100. Zhang, K., Qin, Z., Chang, Y., Liu, J., Malkowski, M.G., Shipa, S., Li, L., Qiu, W., Zhang, J.R. & Li, C. Analysis of a flagellar filament cap mutant reveals that HtrA serine protease degrades unfolded flagellin protein in the periplasm of *Borrelia burgdorferi*. *Mol. Microbiol.* **111**, 1652-1670 (2019).

101. Yang, X., Coleman, A.S., Anguita, J. & Pal, U. A chromosomally encoded virulence factor protects the Lyme disease pathogen against host-adaptive immunity. *PLoS Pathog.* **5**, e1000326 (2009).

102. Yang, X., Lenhart, T.R., Kariu, T., Anguita, J., Akins, D.R. & Pal, U. Characterization of unique regions of *Borrelia burgdorferi* surface-located membrane protein 1. *Infect. Immun.* **78**, 4477-4487 (2010).

103. Yang, X., Lin, Y.P., Heselpoth, R.D., Buyuktanir, O., Qin, J., Kung, F., Nelson, D.C., Leong, J.M. & Pal, U. Middle region of the *Borrelia burgdorferi* surface-located protein 1 (Lmp1) interacts with host chondroitin-6-sulfate and independently facilitates infection. *Cell. Microbiol.* **18**, 97-110 (2016).

104. Koci, J., Bernard, Q., Yang, X. & Pal, U. *Borrelia burgdorferi* surface protein Lmp1 facilitates pathogen dissemination through ticks as studied by an artificial membrane feeding system. *Sci. Rep.* **8**, 1910 (2018).

105. Antonara, S., Chafel, R.M., LaFrance, M. & Coburn, J. *Borrelia burgdorferi* adhesins identified using *in vivo* phage display. *Mol. Microbiol.* **66**, 262-276 (2007).

106. Groshong, A.M., Fortune, D.E., Moore, B.P., Spencer, H.J., Skinner, R.A., Bellamy, W.T. & Blevins, J.S. BB0238, a presumed tetratricopeptide repeat-containing protein, is required during *Borrelia burgdorferi* mammalian infection. *Infect. Immun.* **82**, 4292-4306 (2014).

107. Thakur, M., Sharma, K., Chao, K., Smith, A.A., Herzberg, O. & Pal, U. A protein-protein interaction dictates Borrelial infectivity. *Sci. Rep.* **7**, 2932 (2017).

108. Zhang, X., Yang, X., Kumar, M. & Pal, U. BB0323 function is essential for *Borrelia burgdorferi* virulence and persistence through tick-rodent transmission cycle. *J. Infect. Dis.* **200**, 1318-1330 (2009).

109. Stewart, P.E., Hoff, J., Fischer, E., Krum, J.G. & Rosa, P.A. Genome-wide transposon mutagenesis of *Borrelia burgdorferi* for identification of phenotypic mutants. *Appl. Environ. Microbiol.* **70**, 5973-5979 (2004).

110. Kenedy, M.R., Scott, E.J., 2nd, Shrestha, B., Anand, A., Iqbal, H., Radolf, J.D., Dyer, D.W. & Akins, D.R. Consensus computational network analysis for identifying candidate outer membrane proteins from *Borrelia* spirochetes. *BMC Microbiol.* **16**, 141 (2016).

111. Kung, F., Kaur, S., Smith, A.A., Yang, X., Wilder, C.N., Sharma, K., Buyuktanir, O. & Pal, U. A *Borrelia burgdorferi* surface-exposed transmembrane protein lacking detectable immune responses supports pathogen persistence and constitutes a vaccine target. *J. Infect. Dis.* **213**, 1786-1795 (2016).

112. Shrestha, B., Kenedy, M.R. & Akins, D.R. Outer membrane proteins BB0405 and BB0406 are immunogenic, but only BB0405 is required for *Borrelia burgdorferi* infection. *Infect. Immun.* **85**, e00803-00816 (2017).

113. Thein, M., Bonde, M., Bunikis, I., Denker, K., Sickmann, A., Bergström, S. & Benz, R. DipA, a pore-forming protein in the outer membrane of Lyme disease spirochetes exhibits specificity for the permeation of dicarboxylates. *PLoS One* **7**, e36523 (2012).

114. Bárcena-Uribarri, I., Thein, M., Maier, E., Bonde, M., Bergström, S. & Benz, R. Use of nonelectrolytes reveals the channel size and oligomeric constitution of the *Borrelia burgdorferi* P66 porin. *PLoS One* **8**, e78272 (2013).

115. Kenedy, M.R., Luthra, A., Anand, A., Dunn, J.P., Radolf, J.D. & Akins, D.R. Structural modeling and physicochemical characterization provide evidence that P66 forms a β-barrel in the *Borrelia burgdorferi* outer membrane. *J. Bacteriol.* **196**, 859-872 (2014).

116. Bunikis, J., Noppa, L. & Bergstrom, S. Molecular analysis of a 66-kDa protein associated with the outer membrane of Lyme disease Borrelia. *FEMS Microbiol. Lett.* **131**, 139-145 (1995).

117. Probert, W.S., Allsup, K.M. & LeFebvre, R.B. Identification and characterization of a surface-exposed, 66-kilodalton protein from *Borrelia burgdorferi*. *Infect. Immun.* **63**, 1933-1939 (1995).

118. Coburn, J., Chege, W., Magoun, L., Bodary, S.C. & Leong, J.M. Characterization of a candidate *Borrelia burgdorferi* β_3-chain integrin ligand identified using a phage display library. *Mol. Microbiol.* **34**, 926-940 (1999).

119. Coburn, J. & Cugini, C. Targeted mutation of the outer membrane protein P66 disrupts attachment of the Lyme disease agent, *Borrelia burgdorferi*, to integrin $\alpha_V\beta_3$. *Proc. Natl. Acad. Sci. U. S. A.* **100**, 7301-7306 (2003).

120. Behera, A.K., Durand, E., Cugini, C., Antonara, S., Bourassa, L., Hildebrand, E., Hu, L.T. & Coburn, J. *Borrelia burgdorferi* BBB07 interaction with integrin $\alpha_3\beta_1$ stimulates production of pro-inflammatory mediators in primary human chondrocytes. *Cell. Microbiol.* **10**, 320-331 (2008).

121. Ristow, L.C., Miller, H.E., Padmore, L.J., Chettri, R., Salzman, N., Caimano, M.J., Rosa, P.A. & Coburn, J. The β_3-integrin ligand of *Borrelia burgdorferi* is critical for infection of mice but not ticks. *Mol. Microbiol.* **85**, 1105-1118 (2012).

122. Ristow, L.C., Bonde, M., Lin, Y.P., Sato, H., Curtis, M., Wesley, E., Hahn, B.L., Fang, J., Wilcox, D.A., Leong, J.M., Bergström, S. & Coburn, J. Integrin binding by *Borrelia burgdorferi* P66 facilitates dissemination but is not required for infectivity. *Cell. Microbiol.* **17**, 1021-1036 (2015).

123. Curtis, M.W., Hahn, B.L., Zhang, K., Li, C., Robinson, R.T. & Coburn, J. Characterization of stress and innate immunity resistance of wild-type and Δp66 *Borrelia burgdorferi*. *Infect. Immun.* **86**, e00186-00117 (2018).

124. Caine, J.A. & Coburn, J. A short-term *Borrelia burgdorferi* infection model identifies tissue tropisms and bloodstream survival conferred by adhesion proteins. *Infect. Immun.* **83**, 3184-3194 (2015).

125. Cheung, C.S., Anderson, K.W., Benitez, K.Y., Soloski, M.J., Aucott, J.N., Phinney, K.W. & Turko, I.V. Quantification of *Borrelia burgdorferi* membrane proteins in human serum: a new concept for detection of bacterial infection. *Anal. Chem.* **87**, 11383-11388 (2015).

126. Cugini, C., Medrano, M., Schwan, T.G. & Coburn, J. Regulation of expression of the *Borrelia burgdorferi* β_3-chain integrin ligand, P66, in ticks and in culture. *Infect. Immun.* **71**, 1001-1007 (2003).

127. Brooks, C.S., Hefty, P.S., Jolliff, S.E. & Akins, D.R. Global analysis of *Borrelia burgdorferi* genes regulated by mammalian host-specific signals. *Infect. Immun.* **71**, 3371-3383 (2003).

128. Ntchobo, H., Rothermel, H., Chege, W., Steere, A.C. & Coburn, J. Recognition of multiple antibody epitopes throughout *Borrelia burgdorferi* p66, a candidate adhesin, in patients with early or late manifestations of Lyme disease. *Infect. Immun.* **69**, 1953-1956 (2001).

129. Exner, M.M., Wu, X., Blanco, D.R., Miller, J.N. & Lovett, M.A. Protection elicited by native outer membrane protein Oms66 (p66) against host-adapted *Borrelia burgdorferi*: conformational nature of bactericidal epitopes. *Infect. Immun.* **68**, 2647-2654 (2000).

130. Arnaboldi, P.M. & Dattwyler, R.J. Cross-reactive epitopes in *Borrelia burgdorferi* p66. *Clin. Vaccine Immunol.* **22**, 840-843 (2015).

131. Skare, J.T., Mirzabekov, T.A., Shang, E.S., Blanco, D.R., Erdjument-Bromage, H., Bunikis, J., Bergström, S., Tempst, P., Kagan, B.L., Miller, J.N. & Lovett, M.A. The Oms66 (p66) protein is a *Borrelia burgdorferi* porin. *Infect. Immun.* **65**, 3654-3661 (1997).

132. Skare, J.T., Champion, C.I., Mirzabekov, T.A., Shang, E.S., Blanco, D.R., Erdjument-Bromage, H., Tempst, P., Kagan, B.L., Miller, J.N. & Lovett, M.A. Porin activity of the native and recombinant outer membrane protein Oms28 of *Borrelia burgdorferi*. *J. Bacteriol.* **178**, 4909-4918 (1996).

133. Pinne, M., Thein, M., Denker, K., Benz, R., Coburn, J. & Bergström, S. Elimination of channel-forming activity by insertional inactivation of the *p66* gene in *Borrelia burgdorferi*. *FEMS Microbiol. Lett.* **266**, 241-249 (2007).

134. Bárcena-Uribarri, I., Thein, M., Sacher, A., Bunikis, I., Bonde, M., Bergström, S. & Benz, R. P66 porins are present in both Lyme disease and relapsing fever spirochetes: a comparison of the biophysical properties of P66 porins from six *Borrelia* species. *Biochim. Biophys. Acta* **1798**, 1197-1203 (2010).

135. Petersen, T.N., Brunak, S., von Heijne, G. & Nielsen, H. SignalP 4.0: discriminating signal peptides from transmembrane regions. *Nature methods* **8**, 785-786 (2011).

136. Rahman, O., Cummings, S.P., Harrington, D.J. & Sutcliffe, I.C. Methods for the bioinformatic identification of bacterial lipoproteins encoded in the genomes of Gram-positive bacteria. *World J. Microbiol. Biotechnol.* **24**, 2377–2382 (2008).

137. Sonnhammer, E.L., von Heijne, G. & Krogh, A. A hidden Markov model for predicting transmembrane helices in protein sequences. *Proc. Int. Conf. Intell. Syst. Mol. Biol.* **6**, 175-182 (1998).

138. Hofmann, K. & Stoffel, W. TMbase - a database of membrane spanning proteins segments. *Biol. Chem. Hoppe Seyler* **347**, 166 (1993).

139. Tusnády, G.E. & Simon, I. The HMMTOP transmembrane topology prediction server. *Bioinformatics* **17**, 849-850 (2001).

140. Dong, C., Beis, K., Nesper, J., Brunkan-Lamontagne, A.L., Clarke, B.R., Whitfield, C. & Naismith, J.H. Wza the translocon for *E. coli* capsular polysaccharides defines a new class of membrane protein. *Nature* **444**, 226-229 (2006).

141. Abellón-Ruiz, J., Kaptan, S.S., Baslé, A., Claudi, B., Bumann, D., Kleinekathöfer, U. & van den Berg, B. Structural basis for maintenance of bacterial outer membrane lipid asymmetry. *Nat Microbiol* **2**, 1616-1623 (2017).

142. Seiler, C.Y., Park, J.G., Sharma, A., Hunter, P., Surapaneni, P., Sedillo, C., Field, J., Algar, R., Price, A., Steel, J., Throop, A., Fiacco, M. & LaBaer, J. DNASU plasmid and PSI:Biology-Materials repositories: resources to accelerate biological research. *Nucleic Acids Res.* **42**, D1253-1260 (2014).
